# Supplementary material for: Estimating the effects of non-pharmaceutical interventions on the number of new infections with COVID-19 during the first epidemic wave
Source: PLoS One. 2021 Jun 2;16(6):e0252827. doi: 10.1371/journal.pone.0252827 (PMC8171941; doi:10.1371/journal.pone.0252827)
Supplement: S1 Appendix — The supplementary material contains (1) a detailed description of the method, (2) discussion of methodological aspects in comparison to related work, (3) description and results from a simulation-based study, (4) further descriptives on the timing of non-pharmaceutical interventions, (5) detailed estimation results and model checks, (6) results from the sensitivity analysis, (7) a visual inspection of the model fit, and (8) data on non-pharmaceutical interventions. (PDF) [file pone.0252827.s001.pdf]

Supplementary material:
Estimating the effects of non-pharmaceutical interventions on the number of new infections with COVID-19 during the first epidemic wave

Nicolas Banholzer[1], Eva van Weenen[1], Adrian Lison[1], Alberto Cenedese[1], Arne Seeliger[1], Bernhard Kratzwald[1], Daniel Tschernutter[1], Joan Puig Salles[1], Pierluigi Bottrighi[1], Sonja Lehtinen[2], Stefan Feuerriegel[1], and Werner Vach[3,4,*]

[1]Department of Management, Technology, and Economics, ETH Zurich, Zurich, Switzerland

[2]Department of Environmental Systems Science, ETH Zurich, Zurich, Switzerland

[3]Basel Academy for Quality and Research in Medicine, Basel, Switzerland

[4]Department of Environmental Sciences, University of Basel, Basel, Switzerland

[*]Corresponding author: werner.vach@unibas.ch

Abstract

The supplementary material contains (1) a detailed description of the method, (2) discussion of methodological aspects in comparison to related work, (3) description and results from a simulation-based study, (4) further descriptives on the timing of non-pharmaceutical interventions, (5) detailed estimation results and model checks, (6) results from the sensitivity analysis, (7) a visual inspection of the model fit, and (8) data on non-pharmaceutical interventions.

Contents

1 Method **6**

1.1 Notation . . . 6

1.2 Overall approach . . . 6

1.3 Relating the number of new infections to the number of contagious subjects and the presence of NPIs . . 7

1.4 Relating the number of observed cases to the number of new infections in the previous days . . . 8

1.5 Relating the number of contagious subjects to the number of new infections in the previous days . . . 8

1.6 Relating the probability to be contagious to the generation time distribution . . . 9

1.7 Choice of priors for the parameters of the distribution of the time from infection to reporting . . . 9

1.8 Choice of estimate for the generation time distribution . . . 10

1.9 Choice of the functional form of the time-delayed response function . . . 12

1.10 Modeling and non-modeling phase . . . 12

1.11 Choice of prior distributions for the effect of NPIs . . . 13

1.12 Choice of prior distributions (summary) . . . 14

1.13 Model parameter estimation . . . 15

1.14 Ignoring undetected infections . . . 16

2 Methodological aspects and comparison with related work **17**

3 Simulation-based study **18**

4 Timing of non-pharmaceutical interventions **20**

5 Estimation results **22**

5.1 Estimated model parameters . . . 22

5.2 Checking for correlations between parameters . . . 25

5.3 Checking for influential observations . . . 27

6 Sensitivity analysis **29**

6.1 Modeling phase starting from 10 cumulative cases onward . . . 29

6.2 Modeling phase ending 21 to 35 days after last NPI was implemented . . . 30

6.3 Varying the time-delayed response functions . . . 31

6.4 Varying the prior distribution for the effects of non-pharmaceutical interventions . . . 32

6.5 Varying the prior distribution for the time from infection to reporting of a new case . . . 33

6.6 Varying the generation time distribution . . . 36
6.7 Analyzing the influence of leaving out one country at the time . . . 38
7 Visual inspection of the model fit **41**
8 Data on non-pharmaceutical interventions **52**
8.1 Data collection . . . 52
8.2 Data Sources . . . 53
References **136**

List of Figures

1 Prior choice for the distribution of the time from infection to reporting of a new case . . 11
2 Prior choice for the generation time distribution . . . 11
3 Prior choice for the effects of non-pharmaceutical interventions θ_m. . . . 15
4 Estimated effects based on 50 simulations of fake data for scenario 1 (first row) and 2 (second row), respectively. **(a,c)** Boxplot of the estimated effects (posterior mean with the true effect in red, respectively). **(b,d)** Proportion of simulations where the posterior credible intervals (CrIs) contain the true effect. . . . 19
5 Distribution of the time from infection to reporting of a new case . . . 24
6 Bivariate posterior distributions for the parameters of non-pharmaceutical interventions 26
7 Model diagnostics based on influential observations . . . 28
8 Comparison of the effects of non-pharmaceutical interventions when varying the start of the modeling phase . . . 29
9 Comparison of the effects of non-pharmaceutical interventions when the modeling phase ends 21 or 35 days after the last NPI was implemented . . . 30
10 Comparison of the effects of non-pharmaceutical interventions when varying the time delayed response function . . . 31
11 Comparison of the effects of non-pharmaceutical interventions when varying the prior distribution for the effects of non-pharmaceutical interventions . . . 32
12 Prior choices for the distribution of the time from infection to reporting of a new case in the sensitivity analysis . . . 34

13 Comparison of the effects of non-pharmaceutical interventions when varying the prior choices for distribution of the time from infection to reporting of a new case 35

14 Prior choices for the generation time distribution in the sensitivity analysis 36

15 Comparison of the effects of non-pharmaceutical interventions when varying the generation time distribution 37

16 Comparison of the effects of non-pharmaceutical interventions in a leave-one-country analysis 39

17 Comparison of the effects of non-pharmaceutical interventions when leaving-out Eastern or Western Australia 40

18 Expected number of new infections and new cases and observed number of new cases over time for Australia and Austria 42

18 Expected number of new infections and new cases and observed number of new cases over time for Belgium and Canada 43

18 Expected number of new infections and new cases and observed number of new cases over time for Denmark and Finland 44

18 Estimated number of new infections and new cases and observed number of new cases over time for France and Germany 45

18 Expected number of new infections and new cases and observed number of new cases over time for Greece and Ireland 46

18 Expected number of new infections and new cases and observed number of new cases over time for Italy and Luxembourg 47

18 Expected number of new infections and new cases and observed number of new cases over time for the Netherlands and Norway 48

18 Expected number of new infections and new cases and observed number of new cases over time for Portugal and Spain 49

18 Expected number of new infections and new cases and observed number of new cases over time for Sweden and Switzerland 50

18 Expected number of new infections and new cases and observed number of new cases over time for the United Kingdom and the United States 51

List of Tables

1 Start and end of non-modeling and modeling phase by country. 14

2 Prior choices for model parameters. 15

3 Distance (in days) to the next NPIs in time per contry and NPI. “Average” is the mean distance over all countries. Computation: For each NPI, the distance is measured as the absolute difference (in days) to the NPI that was implemented closest before or after, which is then averaged across countries. For countries where NPIs were implemented at the regional level, the absolute differences were first averaged over all regions. If an NPI was not implemented, then the absolute difference is omitted from computation. 20

4 Pairwise average distance (in days) between the implementation of NPIs across countries. Computation: For each pair of NPIs, the distance is measured as the absolute difference (in days) between the implementation dates of NPIs, which is then averaged across countries. For countries where NPIs were implemented at the regional level, the absolute differences were first averaged over all regions. If an NPI was not implemented, then the absolute difference is omitted from computation. 21

5 Estimation results 23

6 Sources for policies in Switzerland, Austria, Belgium, Denmark, Finland, France, United Kingdom, Greece, Ireland, Luxembourg, Netherlands, Norway, Portugal, Sweden, United States of America, Germany, Italy, Spain, Canada, and Australia 63

7 Sources for policies implemented across different US States 87

8 Sources for policies implemented across different German regions 93

9 Sources for policies implemented across different Spanish regions 104

10 Sources for policies implemented across different Canadian regions 110

11 Sources for policies implemented across different Australian regions 114

12 Sources for policies implemented across different Italian regions 124

13 Sources for policies implemented across different Swiss regions 135

1 Method

1.1 Notation

j	country
t	days since start of observation period
N_{jt}	number of reported new cases in country j at day t
I_{jt}	number of new infections (transmissions) in country j at day t (unobserved)
C_{jt}	number of contagious subjects in country j at day t (unobserved)

To model the impact of a non-pharmaceutical intervention (NPI) in a specific country, we have to take into account that some countries introduced the NPIs only in specific subregions or at different timepoints in different subregions. Hence, with respect to the NPIs, we introduce the following notation:

m	NPI (numbered from 1 to M)
r	subregion r of country j $(r = 1, ..., R_j)$
T_{mrjt}	number of days since NPI m took effect in region r of country j at day t (counting the first day at which the NPI could affect the number of new cases as $t = 1$)
p_{rj}	the share of region's r population of the total population of country j

Note that, also in countries with several regions, only the overall number of new cases is analyzed, not region specific counts.

1.2 Overall approach

The overall aim is to assess the impact of NPI m on the number of new infections in the days after the NPI becomes active. We approach this by modeling the number of new infections as a function of the number of contagious subjects and the presence of active measures. That is, we consider a model linking two unobserved quantities. To obtain a model in the observed quantities N_{jt}, we link this number to the number of new infections in the previous days. Similarly, we link the number of contagious subjects to the number of new infections in the previous days. The overall model is then

fitted using a fully Bayesian approach, which requires the specification of prior distributions for all model parameters.

In the following, we present the three submodels and describe to which degree external knowledge is incorporated to justify choices.

1.3 Relating the number of new infections to the number of contagious subjects and the presence of NPIs

Let θ_m denote the relative reduction of new infections when NPI m is fully implemented, i.e. the fraction of avoided new infections compared to the situation without this NPI. Let $f(t)$ denote a function taking values between 0 and 1 describing the degree of implementation as a function of the time since start of the NPI. In a country j with only one region, $\theta_m f(T_{m1jt})$ describes the fraction of new infections that are avoided in this country at day t. If regions are exposed to NPIs to a varying degree, the corresponding fraction is $\theta_m \sum_{r=1}^{R_j} p_{rj}\, f(T_{mrjt})$.

The overall fraction of *un*-avoided new infections is then given by

$$\prod_{m=1}^{M} \left(1 - \theta_m \sum_{r=1}^{R_j} p_{rj}\, f(T_{mrjt}) \right) . \tag{1}$$

In the absence of any measure, the expected value of the number of new infections $\mu^{I_{jt}}$ would be determined by the number of contagious subjects C_{jt} and the country-specific daily transmission rate δ_j, i.e., $\mu^{I_{jt}} = C_{jt}\ \delta_j$. In the presence of NPIs, we have to multiply this with the fraction of un-avoided infections, resulting in

$$\mu^{I_{jt}} = C_{jt}\ \delta_j \prod_{m=1}^{M} \left(1 - \theta_m \sum_{r=1}^{R_j} p_{rj}\, f(T_{mrjt}) \right) . \tag{2}$$

It would be natural to model the number of new infections as a negative binomial distribution. However, the software used in this work does not allow integer values for unobserved variables. Therefore, a normal distribution was used instead, with the mean and standard deviation analogous to a negative binomial distribution:

$$I_{jt} \sim \text{Lognormal}\left(\mu^{I_{jt}}, \sigma^{I_{jt}} \right) \ , \tag{3}$$

with $\sigma^{I_{jt}} = \sqrt{\mu^{I_{jt}}\left(1+\frac{\mu^{I_{jt}}}{\phi^I}\right)}$, and an overdispersion parameter ϕ^I.

1.4 Relating the number of observed cases to the number of new infections in the previous days

The expected number $\mu^{N_{jt}}$ of new cases N_{jt} in country j at day t can be derived from the number of new infections in the previous days as

$$\mu^{N_{jt}} = \sum_{s<t} I_{js} \cdot p_{\mathrm{IN}}(t-s) \ , \tag{4}$$

where $p_{\mathrm{IN}}(t)$ denotes the probability that a new infected subject is reported at day t after the infection, i.e., becomes a new case. It is assumed that $p_{\mathrm{IN}}(t)$ is a discretized version of a lognormal distribution. This distribution is estimated from our data as part of fitting the overall model using a weakly informative prior, as described later.

The observed number of new cases are modeled to follow a negative binomial distribution (NB), i.e.,

$$N_{jt} \sim \mathrm{NB}\left(\mu^{N_{jt}}, \sigma^{N_{jt}}\right) \tag{5}$$

with mean $\mu^{N_{jt}}$, standard deviation $\sigma^{N_{jt}} = \sqrt{\mu^{N_{jt}}\left(1+\frac{\mu^{N_{jt}}}{\phi^N}\right)}$, and an overdispersion parameter ϕ^N.

1.5 Relating the number of contagious subjects to the number of new infections in the previous days

The expected number $\mu^{C_{jt}}$ of contagious subjects in country j at day t can be derived from the number of new infections in the previous days as

$$\mu^{C_{jt}} = \sum_{s<t} I_{js} p_{\mathrm{IC}}(t-s) \ , \tag{6}$$

where $p_{\mathrm{IC}}(t)$ denotes the probability that a new infected subject is contagious at day t after the infection. Since our data does not include information about these probabilities, we make a choice based on external information and considerations presented in the next section. Moreover, we regard

the relation between I_{jt} and C_{jt} as a deterministic one, i.e., $C_{jt} = \mu^{C_{jt}}$.

1.6 Relating the probability to be contagious to the generation time distribution

Let γ denote the probability that a randomly chosen contagious subject infects another randomly chosen subject within one day. Considering the probabilities $q(t)$ that an infected subject infects another randomly chosen subject at day t after his/her own infection, there is the simple relation

$$q(t) = p_{\mathrm{IC}}(t)\,\gamma\ . \tag{7}$$

Now, let $p_{\mathrm{G}}(t)$ denote the density of the generation time distribution. That is, given a subject has infected another subject, $p_{\mathrm{G}}(t)$ is the probability that this has happened t days after his/her infection. It follows that there is a simple relation

$$p_{\mathrm{G}}(t) = \frac{q(t)}{\sum_t q(t)} = \frac{p_{\mathrm{IC}}(t)\,\gamma}{\sum_t p_{\mathrm{IC}}(t)\,\gamma} = \frac{p_{\mathrm{IC}}(t)}{\sum_t p_{\mathrm{IC}}(t)} \tag{8}$$

showing that $p_{IC}(t)$ is proportional to the generation time distribution. Consequently, an estimate for the generation time distribution is needed. The proportionality factor can be omitted because $\mu^{I_{jt}}$ is multiplicative in C_{jt}, and thus the omitted factor can be subsumed into δ_j.

Approximating p_{IC} by the generation time distribution has been also done by Cori et al[1]. A more rigorous discussion about the validity of this approximation is given by Fraser et al[2]. Since we model the ratio between the expected number of new infections and the number of contagious subjects, our approach can be also interpreted as modeling the course of the reproduction number over time.

1.7 Choice of priors for the parameters of the distribution of the time from infection to reporting

The time from infection to reporting of a new case can be written as a sum of the incubation period and the time from symptom onset to reporting (i.e., the reporting delay). Estimates for these individual distributions can be found in the literature. A rapid systematic review and meta

analysis of observational research on the incubation period[3] arrived at a lognormal distribution for the incubation period and reported estimates of $\mu = 1.63$ for the mean and of $\sigma = 0.50$ for the standard deviation of the natural logarithm. This translates into an average incubation period of about six days. A study on the COVID-19 outbreak in Italy[4] arrived at a Gamma distribution for the reporting delay and reported estimates of $\alpha = 1.88$ for the shape and $\beta = 0.26$ for the inverse scale. This translates into an average reporting delay of about seven days, which is in line with estimates from other countries[5–8].

It is reasonable to assume that the incubation period and the reporting delay are independent. Following this assumption, the distribution for the time from infection to reporting of a new case is the sum of i.i.d. random variables and thus we can compute the mean and variance as the sum of the individual means and variances. Assuming for the sum again a lognormal distribution hence leads to the choice of a Lognormal($\mu = 2.47, \sigma = 0.45$). This translates into an average delay for the time from infection to reporting of a new case of about thirteen days with a standard deviation of six days. However, a fixed choice for p_{IN} would neglect the uncertainty about the shape of the distribution and thus the parameters of the lognormal distribution were instead estimated from our data as part of fitting the overall model. To do so, weakly informative priors are chosen for the mean $\mu \sim \mathrm{Normal}(2.47, 0.50)$ and standard deviation $\sigma \sim \mathrm{Gamma}(2.00, 4.48)$ (since $\frac{2.00}{4.448} \approx 0.45$) of the natural logarithm. Our choices reflect prior knowledge about the delay's mean and standard deviation, while taking uncertainty about their estimates into account.

The prior distributions for μ and σ as well as the resulting prior distributions for the discrete probabilities $p_{\mathrm{IN}}(t)$ are shown in Fig 1. Note that p_{IN} is discretized via $p_{\mathrm{IN}}(s) = \int_0^{0.5} p_{\mathrm{IN}}(\tau)\, \mathrm{d}\tau$ for $s = 0$ and $p_{\mathrm{IN}}(s) = \int_{s-0.5}^{s+0.5} p_{\mathrm{IN}}(\tau)\, \mathrm{d}\tau$ for $s = 1, 2, \ldots$, where $p_{\mathrm{IN}}(\tau) \sim \mathrm{Lognormal}(\mu, \sigma)$ is the density of the lognormal distribution with mean μ and standard deviation σ.

1.8 Choice of estimate for the generation time distribution

The generation time distribution is often approximated by the serial interval distribution (e.g.,[9]). However, it was recently pointed out that this approximation is based on the assumption that the incubation period and the infectiousness profile are independent[10], which is questionable. Other studies estimated the generation time distribution from data on transmission pairs[11,12], thereby making the implicit assumption that the generation time is independent of the incubation period[10],

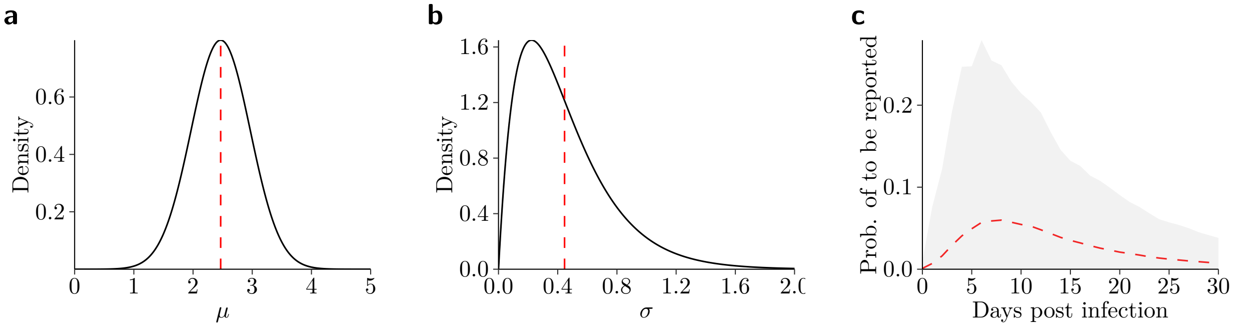

Fig 1. Prior choice for the distribution of the time from infection to reporting of a new case. **(a)** Log mean μ (prior mean as dashed red line). **(b)** Log standard deviation σ (prior mean as dashed red line). **(c)** Distribution of $p_{\mathrm{IN}}(t)$ for $t = 0, 1, \ldots, 30$ (prior mean as dashed red line with 95 % range as shaded area, based on 4,000 independent draws from the distributions for μ and σ).

which is also questionable.

By leveraging data on the exposure for both index and secondary cases, a recent study inferred the generation distribution more accurately[13] without having to make the above assumptions. This study suggests to use a Weibull(3.28, 6.12) distribution, which has a mean of 5.49 days and a standard deviation of 1.84 days.

The discrete form of this distribution is depicted in Fig 2. Again, the distribution is discretized via $p_{\mathrm{G}}(s) = \int_0^{1.5} p_{\mathrm{G}}(\tau)\,\mathrm{d}\tau$ for $s = 1$ and $p_{\mathrm{G}}(s) = \int_{s-0.5}^{s+0.5} p_{\mathrm{G}}(\tau)\,\mathrm{d}\tau$ for $s = 2, 3, \ldots,$ where $p_{\mathrm{G}}(\tau) \sim$ Weibull(3.28, 6.12) is the density of the Weibull distribution with shape α and scale κ. Note that we explicitly set $p_{\mathrm{IC}}(0)$ to 0, avoiding the challenge to include new infections at one day in the number of contagious subjects the same day.

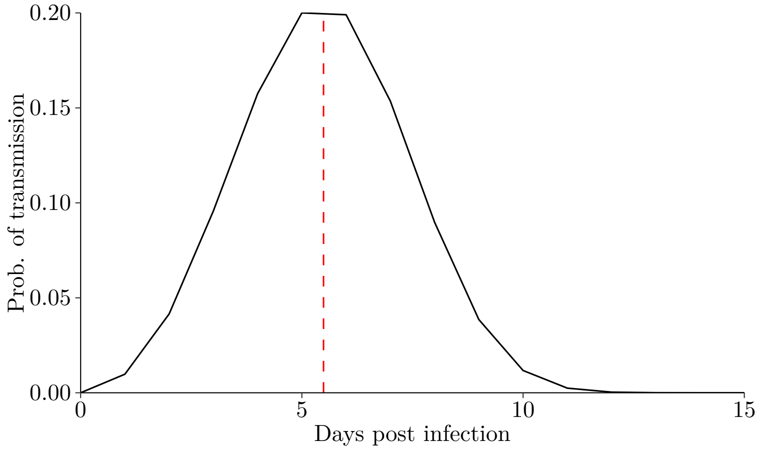

Fig 2. Prior choice for the generation time distribution $p_{\mathrm{G}}(t)$.

1.9 Choice of the functional form of the time-delayed response function

We model the time-delayed response function with a first-order spline

$$f(t)=\begin{cases}0, & \text{if } t\le t_0,\\ \frac{t-t_0}{t_1-t_0}, & \text{if } t_0<t<t_1,\\ 1, & \text{if } t\ge t_1.\end{cases} \tag{9}$$

This means that the effect of an NPI increases linearly after its implementation until t_1. We set $t_0=0$ days and $t_1=3$ days, reflecting a three day period until people fully respond to the implemented NPI.

1.10 Modeling and non-modeling phase

It took some time for countries to set up reporting practices so that case numbers at the very beginning of the epidemic are often missing or irregular. That is why, in each country, modeling of the number of new cases starts after 100 cumulative cases were reported, i.e., the first day at which the number of cumulative cases exceeds 100 is set as $t=1$. Before that, the number of new cases is seeded similar to Ref.[9]. That is, in the non-modeling phase, the modeling of N_{jt} is ignored, but the other components are still used and, in particular, $\mu^{I_{jt}}$ and $\mu^{C_{jt}}$ are computed. The start of the non-modeling phase is determined as the day at which we would expect the first case when reaching 100 cumulative cases at day $t=1$, given the reproduction number, mean generation time and reporting delay as specified. That is,

$$t=1-\frac{\log 100}{\log 3.28}\cdot 5.49+13.01\approx -33, \tag{10}$$

where $R_0=3.28$ is the basic reproductive number taken from a meta analysis across countries[14], 5.49 days is the mean generation time and 13.01 days is the mean time from infection to reporting. For $I_{j\,-33}$, we replace the recursive formula of our model with

$$I_{j\,-33}\sim \text{Exponential}\left(\frac{1}{\lambda}\right) \tag{11}$$

and choose $\lambda \sim \text{Exponential}(1)$ as a prior distribution. Thereby, it is expected that one person is infected at day $t=-33$, but the estimated number can vary substantially by country. Using the recursive formula, the number of contagious subjects at the start of the non-modeling phase are computed from the initially infected seven days before the non-modeling phase. The initially infected are computed by assuming an exponential growth rate of $\frac{\log 3.28}{5.49}$, i.e.,

$$I_{jt} = I_{j\,-33} \cdot \frac{1}{\exp\left(\frac{\log 3.28}{5.49} \cdot (-33-t)\right)},\ t=-40,-39,\ldots,-34.$$

Note that the initially infected are only computed to smooth the number of infected and contagious subjects at the start of the non-modeling phase.

The end of the modeling phase is, in each country, set to 28 days after the last NPI was implemented in any of the country's regions. This provides sufficient time for the effects of NPIs to show up in the number of new cases, thereby concluding the first wave of the epidemic, as most countries saw their case numbers reverting back after all NPIs were in place. The start of the non-modeling phase as well as the start and end of the modeling phase are shown in Table 1. We made only two adjustments. In Australia, Queensland closed schools on April 20, while all other regions implemented their NPIs before April 2, thus we set April 2 plus 28 days for the end of the modeling phase in Australia. Similarly, in Canada, Northwest Territories canceled gatherings on April 11 while all other territories implemented their NPIs before April 1, thus we set April 1 plus 28 days for the end of the modeling phase in Canada.

1.11 Choice of prior distributions for the effect of NPIs

Our primary goal is to infer the effect of NPIs. These policy measures were implemented to reduce new infections via social distancing. We wanted to construct a prior for the effect of NPIs that (1) has support $\theta_m \in (-\infty, 1)$, thereby not precluding a negative effect corresponding to an increase in new infections from NPIs but also allowing a single NPI to account for a complete elimination of transmissions, (2) gives higher probability to positive effects than to negative effects , and (3) is little informative with respect to the magnitude of a positive effect.

The desired properties were accomplished with a mixture of a half normal distribution for

Country	Non-modeling phase Start	Modeling phase Start	End
Australia	Feb 11	Mar 10	Apr 30
Austria	Feb 09	Mar 08	Apr 16
Belgium	Feb 07	Mar 06	Apr 17
Canada	Feb 12	Mar 11	Apr 29
Denmark	Feb 11	Mar 10	Apr 15
Finland	Feb 14	Mar 13	Apr 16
France	Feb 01	Feb 29	Apr 14
Germany	Feb 02	Mar 01	Apr 20
Greece	Feb 14	Mar 13	Apr 20
Ireland	Feb 15	Mar 14	Apr 25
Italy	Jan 26	Feb 23	Apr 23
Luxembourg	Feb 18	Mar 17	Apr 15
Netherlands	Feb 07	Mar 06	Apr 20
Norway	Feb 07	Mar 06	Apr 13
Portugal	Feb 14	Mar 13	Apr 19
Spain	Feb 03	Mar 02	Apr 27
Sweden	Feb 07	Mar 06	Apr 24
Switzerland	Feb 06	Mar 05	Apr 22
United Kingdom	Feb 03	Mar 02	Apr 20
United States	Feb 05	Mar 04	May 05

Table 1. Start and end of non-modeling and modeling phase by country.

negative effects and a uniform distribution for positive effects. The formal specification

$$\theta_m \sim \text{Mixture}(w) = \begin{cases} \text{Normal}^{-}(0,\sigma) & \text{with probability } w, \\ \text{Uniform}(0,1) & \text{with probability } (1-w). \end{cases} \tag{12}$$

where $w \in [0,1]$ is the mixing ratio. For efficient sampling, the prior density should rather be continuous and thus σ is chosen based on w such that this is the case, i. e.,

$$\sigma = \frac{w}{(1-w)\sqrt{2\pi}} \,. \tag{13}$$

As a default, a mixing ratio of $w = 0.1$ is chosen, which implies a 10% probability that NPIs can lead to an increase in new infections, resulting in $\sigma = 0.04$. The density of this prior is shown in Fig 3.

1.12 Choice of prior distributions (summary)

Table 2 provides an overview of the model parameters together with the choice of priors. If not tailored to the specifics of our model, the choice of priors are informed by recommendations on the choice of priors from the Stan Development Team[15].

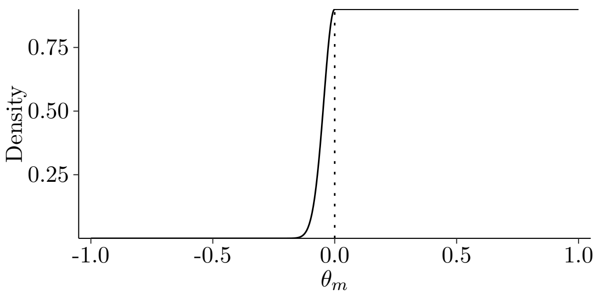

Fig 3. Prior choice for the effects of non-pharmaceutical interventions θ_m.

Parameter	Notation	(Hyper-)Prior
NPIs	θ_m	$\text{Mixture}(0.1) = \begin{cases} \text{Normal}^{-}(0, 0.04) & \text{with prob. } 0.1 \\ \text{Uniform}(0,1) & \text{with prob. } 0.9 \end{cases}$
Country-specific daily transmission rate	δ_j	$\delta_j = \exp(\alpha + \alpha_j)$
	α	$\text{Student}-\text{t}(\nu = 7, \mu = 0, \sigma = 10)$
	α_j	$\text{Normal}(0, \tau)$
	τ	$\text{Student}-\text{t}^{+}(\nu = 4, \mu = 0, \sigma = 1)$
Overdispersion	ϕ^N	$\phi^N = \left(\frac{1}{\xi^N}\right)^2$
	ξ^N	$\text{Normal}^{+}(\mu = 0, \sigma = 1)$
	ϕ^I	$\phi^I = \left(\frac{1}{\xi^I}\right)^2$
	ξ^I	$\text{Normal}^{+}(\mu = 0, \sigma = 1)$
Time from infection to new case	p_{IN}	$\text{Lognormal}(\mu, \sigma)$
	μ	$\text{Normal}(\mu = 2.47, \sigma = 0.45)$
	σ	$\text{Gamma}(\alpha = 2.00, \beta = 4.48)$
Generation time	p_{G}	$\text{Weibull}(\alpha = 3.28, \kappa = 6.12)$
Initially infected subjects	I_{j-33}	$\text{Exponential}\left(\frac{1}{\lambda}\right)$
	λ	$\text{Exponential}(1)$

Table 2. Prior choices for model parameters.

1.13 Model parameter estimation

Model parameters are estimated with a Bayesian approach. Specifically, Markov chain Monte Carlo (MCMC) sampling is used as implemented by the Hamiltonian Monte Carlo algorithm with the No-U-Turn Sampler (NUTS) from Stan 2.19.2[16]. If not stated otherwise, we report posterior means and credible intervals (CrIs) based on the 2.5% and 97.5% quantile of the posterior samples.

Each model is estimated with 4 Markov chains and 2,000 iterations of which the first 1,000 iterations are discarded as part of the warm-up. Estimation power is evaluated via the ratio of the effective sample size ($\hat{n}_{\text{eff}}/N$), and convergence of the Markov chains is assessed with the Gelman-Rubin convergence diagnostic ($\hat{R}$). Further checks pertain to the detection of influential observations and correlations between the parameters of interest.

1.14 Ignoring undetected infections

In our considerations, we ignore that probably many infected subjects remain undetected as we implicitly assume that all infected case are detected ($p_{\mathrm{IN}}(t)$ sums up to 1). Formally, we could try to take this into account by introducing the probability of an infected case to be observed. However, this would act mainly on the global intercept α' (except that the shape of a negative binomial distribution depends slightly on the actual sample size). Hence it seems to be safe to ignore this.

2 Methodological aspects and comparison with related work

Flaxman et al.[9] were the first who attempted to link NPIs to observed cases or deaths using a semi-mechanistic Bayesian hierarchical model. Both the study by Brauner et al.[17] and our study can be seen as extensions of this approach, thereby making use in particular of data from more countries. This also implies the possibility of refined modeling.

The above studies have in common that they model the effect of the NPIs on the number of new infections. Whereas the other studies approached this by modeling explicitly the effect on the reproduction number, we directly modelled the number of new infections in relation to the number of contagious subjects. Thereby, the generation time distribution was used in a way to approximate the time from infection to becoming contagious[1].

A further common property of the above studies is the use of prior information on the distribution of certain quantities that play a central role in the spread of infectious diseases. Flaxman et al. equal the generation time distribution with the serial distribution, whereas Brauner et al. and our study avoid this, considering that there can be a substantial difference between these distributions[10]. Brauner et al. estimated the generation time distribution from their data on cases and deaths using an informative prior, whereas we made use of an explicit assumption based on prior knowledge. The second quantity used in both studies is the time from infection to reporting, which is assumed to be known by Flaxman et al. and estimated by Brauner et al. and our study. Similar to the other studies, we investigated the sensitivity of the estimated NPI effects with respect to the assumptions made or the priors chosen.

A specific property of our approach is to take the regional variation in the implementation of NPIs explicitly into account. We approached this by incorporating the share of the country's total population that is affected by active NPIs in our model. The other studies ignored this variation or restricted the analysis to countries with no or very little regional variation. A further specific property was to allow for a gradual increase in the response to NPIs over the first few days, whereas the other studies assumed a full response on the first day NPIs were implemented.

3 Simulation-based study

The close succession in which NPIs were implemented and the complexity of our model may raise concerns regarding the identifiability of the effects of NPIs. To alleviate such concerns, we conduct a simulation-based study in order to show that our model can recover the true effects.

The simulation-based study is conducted as follows. First, we set our model parameters to fixed values. For the effects of the NPIs θ_m, we consider two scenarios as described below. All nuisance parameters are set to their prior mean (e.g., $\mu^{p_{IN}}$ and $\sigma^{p_{IN}}$) or close to their posterior mean from our default model (e.g., in the case of δ_j). Second, we simulate fake data for the number of new cases from our model based on the NPIs that were implemented in each country over time (but with hypothetical effects θ_m assigned to them). Third, the simulated number of new cases are used as model input for estimating the effects of NPIs.

Two scenarios are considered to check whether our model can recover the true effects θ_m. In the first scenario, we assign a large effect to one NPI ($\theta_{\text{School closure}} = 0.5$), a modest effect to a second NPI ($\theta_{\text{Bans on small gatherings}} = 0.25$), and small effects to all other NPIS ($\theta_{m \backslash \text{School closure, Bans on large gatherings}} = 0.05$). This scenario is intended to show that our model can identify large effects and distinguish them from small ones. In the second scenario, we assign a modest effect to all NPIs ($\theta_m = 0.15$). This scenario is intended to show that the timing of NPIs does not influence our estimation, which is something we were concerned about after seeing that the most effective measures in our study were the ones implemented earlier.

The results from our simulation-based study are shown in Fig 4 As expected, there is considerable uncertainty in the estimated effects in both scenarios, but the estimated effects are reasonably close to the true effects. More importantly, the credible intervals contain the true effect in most simulations. In fact, we would expect the credible interval to contain the true effect 95% of the time. Note that our estimated proportions may approach the 95% as we increase the number of simulations. Overall, our simulation-based study suggests that our model can identify the effects of NPIs.

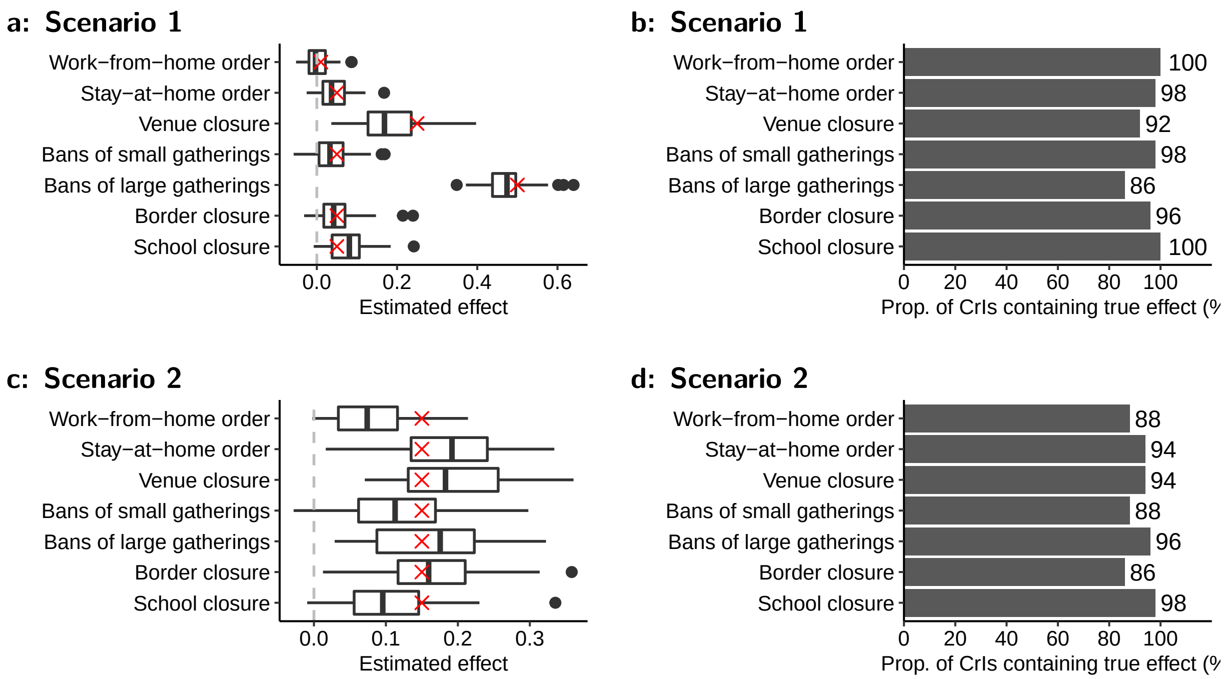

Fig 4. Estimated effects based on 50 simulations of fake data for scenario 1 (first row) and 2 (second row), respectively. **(a,c)** Boxplot of the estimated effects (posterior mean with the true effect in red, respectively). **(b,d)** Proportion of simulations where the posterior credible intervals (CrIs) contain the true effect.

4 Timing of non-pharmaceutical interventions

	Ban of small gatherings	School closure	Venue closure	Ban of large gatherings	Border closure	Stay-at-home order	Work-from-home order
Australia	4.0	7.0	3.0	4.1	3.0	3.2	
Austria	5.0	3.0	3.0	3.0	3.0	3.0	
Belgium	4.0	2.0	2.0	2.0	2.0	2.0	
Canada	2.9	2.5	2.5	5.0	2.5	5.0	6.0
Denmark	2.0	2.0	2.0	2.0	2.0		
Finland	3.0	3.0	3.0	3.0	3.0		
France	1.0	1.0	1.0	1.0	1.0	1.0	1.0
Germany	3.9	3.1	3.0	6.1		4.0	
Greece	1.0	2.0	1.0	4.0		5.0	
Ireland	3.0	3.0	3.0	13.0		13.0	13.0
Italy	4.0	4.0	4.9	4.0	4.0	4.9	4.0
Luxembourg	5.0	1.0	1.0	1.0		1.0	1.0
Netherlands	4.0	4.0	4.0	7.0			
Norway	1.0	1.0	1.0		3.0		
Portugal	1.0	1.0	6.0	6.0		6.0	
Spain	1.7	1.2	1.4	1.3	1.4	1.3	13.0
Sweden	16.0			16.0			
Switzerland	16.9	1.5	1.5	3.3	5.2		2.0
United Kingdom	4.0	3.0	3.0	3.0		3.0	
United States	1.8	2.0	3.0	2.0	2.3	8.2	7.1
Average	4.3	2.5	2.6	4.6	2.7	4.3	5.9

Table 3. Distance (in days) to the next NPIs in time per contry and NPI. "Average" is the mean distance over all countries. Computation: For each NPI, the distance is measured as the absolute difference (in days) to the NPI that was implemented closest before or after, which is then averaged across countries. For countries where NPIs were implemented at the regional level, the absolute differences were first averaged over all regions. If an NPI was not implemented, then the absolute difference is omitted from computation.

	Ban of large gatherings	School closure	Venue closure	Ban of small gatherings	Border closure	Stay-at-home order	Work-from-home order
Ban of large gatherings	0.0	4.1	4.3	7.4	7.6	9.1	12.4
School closure	4.1	0.0	2.5	4.4	5.4	6.4	8.9
Venue closure	4.3	2.5	0.0	3.4	4.8	4.9	7.1
Ban of small gatherings	7.4	4.4	3.4	0.0	5.2	2.7	6.9
Border closure	7.6	5.4	4.8	5.2	0.0	6.8	7.8
Stay-at-home order	9.1	6.4	4.9	2.7	6.8	0.0	4.7
Work-from-home order	12.4	8.9	7.1	6.9	7.8	4.7	0.0

Table 4. Pairwise average distance (in days) between the implementation of NPIs across countries. Computation: For each pair of NPIs, the distance is measured as the absolute difference (in days) between the implementation dates of NPIs, which is then averaged across countries. For countries where NPIs were implemented at the regional level, the absolute differences were first averaged over all regions. If an NPI was not implemented, then the absolute difference is omitted from computation.

5 Estimation results

5.1 Estimated model parameters

Table 5 presents posterior means and credible intervals for all model parameters. See the main paper for a discussion of the effects of NPIs. Here we briefly discuss some of the additional model parameters.

The ratio of the effective sample size ($\hat{n}_{\text{eff}}/N$) and the Gelman-Rubin convergence diagnostic ($\hat{R}$) indicates good estimation power. It further suggests that the Markov chains converged.

There is a country-specific variation in the intercept parameter (α_j), reflecting differences in the rate of new cases – i.e., spread of the disease – in the absence of any NPI. Australia, Norway and Sweden are among the countries with lowest estimated rate and Canada, Ireland, and the US are among the countries with highest estimated rate.

The expected number of new infections at start of the non-modeling phase is around 4, but the exact number varies between countries, with around 11 infected in Australia and 1 infected in the US.

The overdispersion parameter (ϕ) can be rather precisely estimated to be in the magnitude of 4 for the number of new cases and 8 for the number of new infections, i.e., we are far away from the case of no overdispersion ($\phi = \infty$). This coincides with the empirical observation of rather unsmooth trajectories in each country (Section 7).

The posterior distribution of the parameters of the log normal distribution describing the time from infection to reporting suggest that we can estimate them with a rather high precision (Fig 5a-b). The posterior mean of $\mu^{p_{\text{IN}}} = 2.62$ and $\sigma^{p_{\text{IN}}} = 0.18$ corresponds to a mean delay of about 15 days, which is slightly higher when compared to the prior. The posterior distribution of sigma is placed distinctly below the prior mean value of 0.4. Both together implies a rather precise posterior knowledge about the distribution of the time from infection to reporting with a range from roughly 8 to 25 days.

	Mean	Lower CrI	Upper CrI	$\hat{n}_{\text{eff}}/N$	$\hat{R}$
α	0.83	0.73	0.94	0.24	1.00
τ	0.20	0.13	0.29	0.67	1.00
α_1	−0.32	−0.48	−0.17	0.35	1.00
α_2	−0.11	−0.27	0.04	0.36	1.00
α_3	0.18	0.04	0.33	0.32	1.00
α_4	0.24	0.10	0.38	0.27	1.00
α_5	−0.12	−0.28	0.04	0.33	1.00
α_6	−0.05	−0.21	0.12	0.26	1.00
α_7	0.16	0.02	0.32	0.30	1.00
α_8	0.02	−0.12	0.15	0.35	1.00
α_9	−0.13	−0.29	0.02	0.34	1.00
α_{10}	0.20	0.05	0.36	0.38	1.00
α_{11}	0.06	−0.06	0.19	0.32	1.00
α_{12}	−0.18	−0.35	−0.01	0.32	1.00
α_{13}	0.01	−0.13	0.16	0.33	1.00
α_{14}	−0.25	−0.41	−0.10	0.35	1.00
α_{15}	0.01	−0.14	0.16	0.33	1.00
α_{16}	0.12	−0.02	0.26	0.31	1.00
α_{17}	−0.18	−0.35	0.00	0.19	1.01
α_{18}	0.00	−0.15	0.16	0.21	1.01
α_{19}	0.04	−0.10	0.17	0.37	1.00
α_{20}	0.33	0.20	0.47	0.30	1.00
θ_1	0.17	−0.02	0.36	0.45	1.00
θ_2	0.10	−0.02	0.21	0.30	1.00
θ_3	0.37	0.21	0.50	0.09	1.02
θ_4	0.09	−0.04	0.23	0.36	1.00
θ_5	0.18	−0.04	0.40	0.16	1.01
θ_6	0.04	−0.06	0.17	0.43	1.00
θ_7	0.01	−0.08	0.12	0.64	1.00
λ	4.41	2.39	7.32	0.14	1.00
I_{1-33}	11.02	3.08	24.93	0.18	1.01
I_{2-33}	7.20	1.84	17.59	0.22	1.00
I_{3-33}	1.22	0.14	3.59	0.34	1.00
I_{4-33}	1.04	0.10	3.28	0.27	1.00
I_{5-33}	7.94	2.07	18.97	0.19	1.01
I_{6-33}	2.83	0.42	8.02	0.21	1.00
I_{7-33}	1.90	0.30	5.45	0.25	1.00
I_{8-33}	5.07	1.30	12.67	0.20	1.01
I_{9-33}	3.99	0.80	10.17	0.23	1.00
I_{10-33}	1.28	0.15	3.99	0.41	1.00
I_{11-33}	5.29	1.38	12.73	0.18	1.01
I_{12-33}	10.50	2.73	25.32	0.21	1.00
I_{13-33}	3.62	0.76	9.27	0.29	1.00
I_{14-33}	10.46	3.01	23.95	0.17	1.00
I_{15-33}	4.78	1.06	12.39	0.22	1.01
I_{16-33}	3.78	0.78	9.49	0.23	1.00
I_{17-33}	7.19	1.85	17.26	0.36	1.00
I_{18-33}	6.31	1.61	15.45	0.26	1.00
I_{19-33}	3.36	0.71	8.49	0.25	1.00
I_{20-33}	1.28	0.16	3.82	0.18	1.01
ϕ^N	4.24	3.80	4.71	0.72	1.00
ϕ^I	8.66	6.70	11.44	0.05	1.02
$\mu^{p_{IN}}$	2.62	2.51	2.73	0.05	1.03
$\sigma^{p_{IN}}$	0.18	0.11	0.27	0.29	1.00

Table 5. Estimation results for the main analysis. The ratio $\hat{n}_{\text{eff}}$ / N is the effective sample size ($\hat{n}_{\text{eff}}$) divided by the total sample size (N). Generally, ratios above 0.5 correspond to high, between 0.1 and 0.5 to medium and below 0.1 to low estimation power[18]. $\hat{R}$ is the Gelman-Rubin convergence diagnostic[19]. A $\hat{R} \approx 1.00$ indicates good convergence, while a $\hat{R} > 1.10$ indicates bad convergence[20].

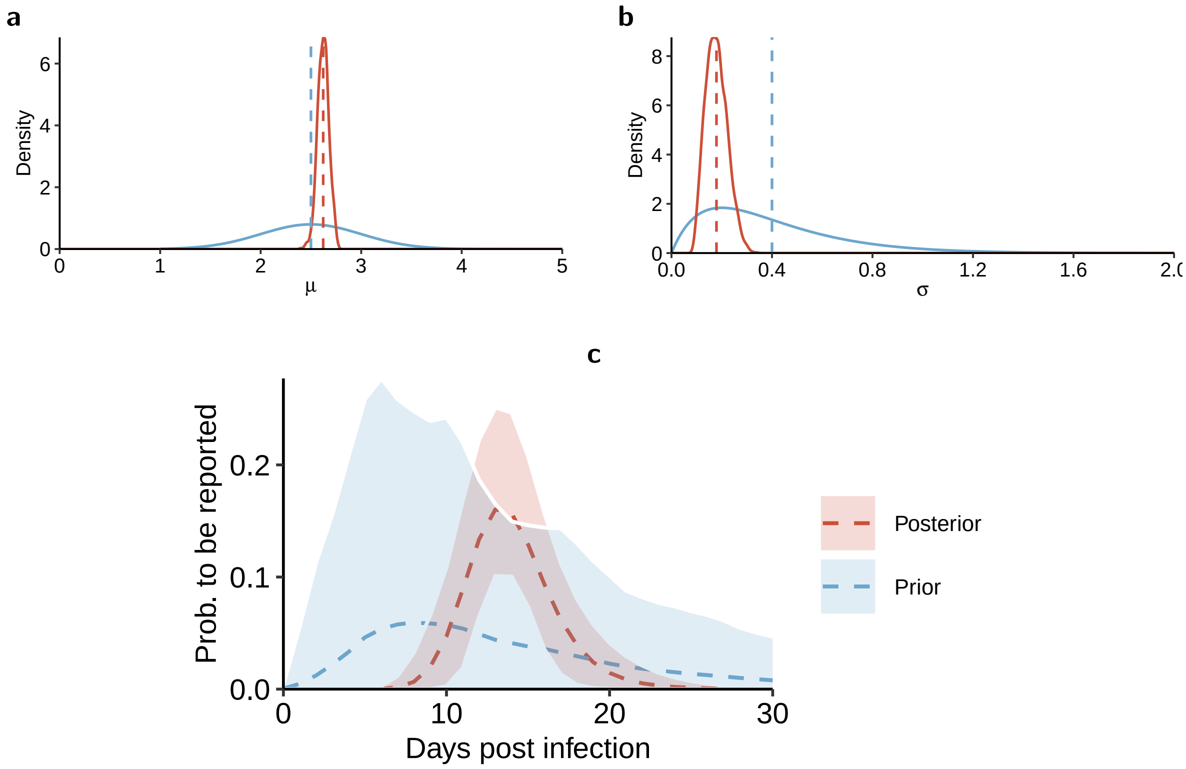

Fig 5. Distribution of the time from infection to reporting of a new case. **(a)** Log mean μ (prior and posterior mean as dashed lines) **(b)** Log standard deviation σ (prior and posterior mean as dashed lines). **(c)** Posterior distribution of $p_{\text{IN}}(t)$ for $t = 0, 1, \ldots, 30$ (prior and posterior mean as dashed lines with 95 % range and 95 % credible interval as shaded area, based on 4,000 draws from the prior and posterior distributions for μ and σ, respectively).

5.2 Checking for correlations between parameters

A similar timing could make it difficult to distinguish the individual effects of NPIs. To investigate this issue, Fig 6 depicts the pairwise bivariate posterior distributions of the parameters of the NPIs. We observe a tendency towards negative correlations, reflecting the difficulty to distinguish the effects of NPIs, which were often introduced close in time. That is, highest negative correlations were observed for the pair of NPIs with the smallest average distance in implementation (Fig 4).

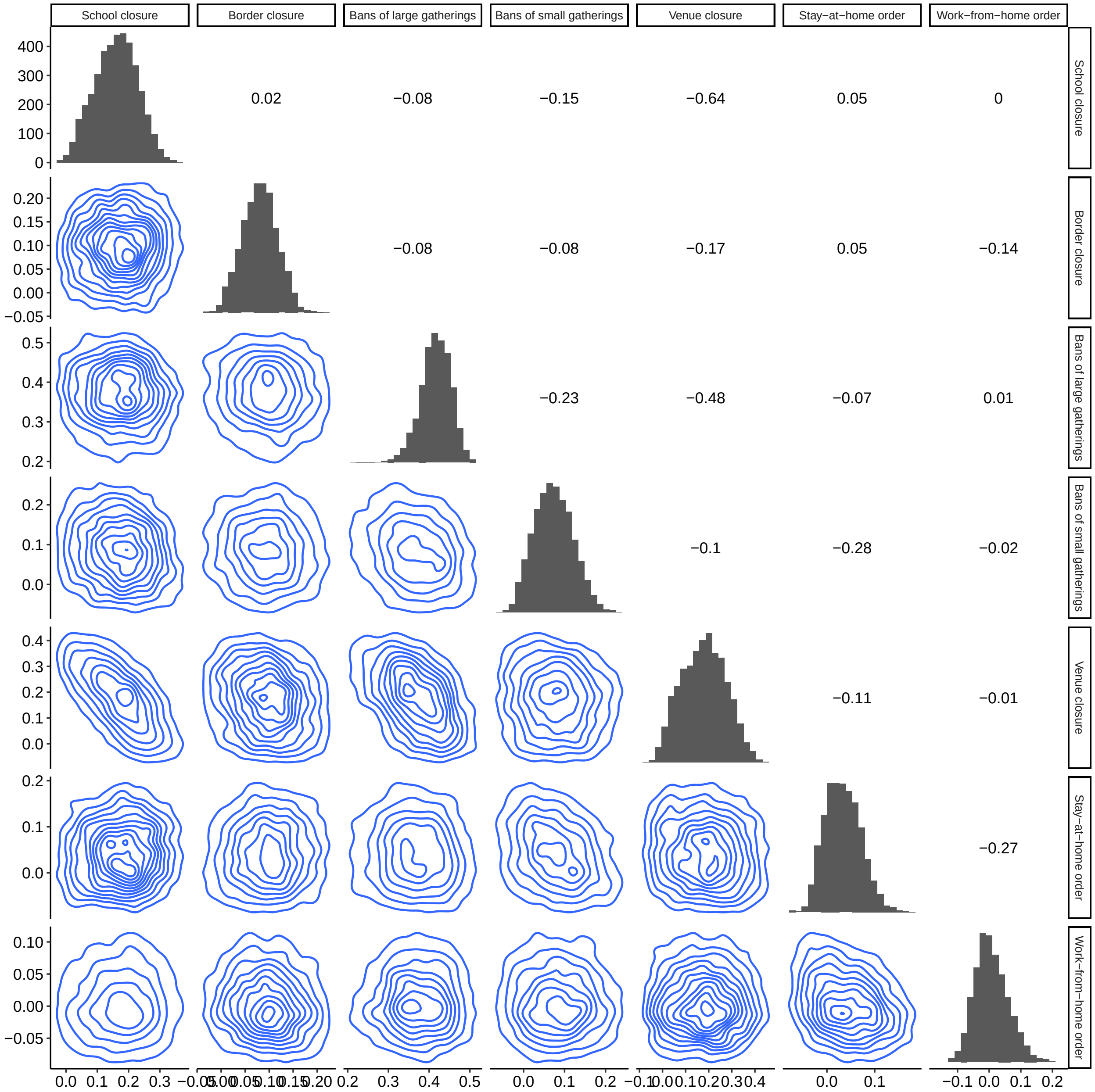

Fig 6. Bivariate posterior distributions visualized by contour plots (lower diagonal matrix) from the MCMC sample for the parameters of non-pharmaceutical interventions (NPIs). Pearson's r is shown in the upper diagonal matrix, and the marginal distribution is depicted as a histogram on the diagonal of the matrix.

5.3 Checking for influential observations

Influential observations can affect parameter estimates. To check for influential observations, Fig 7 shows the tail shape parameter k from approximate leave-one-out cross-validation using Pareto smoothed importance sampling[21]. Only few observations seem to be highly influential.

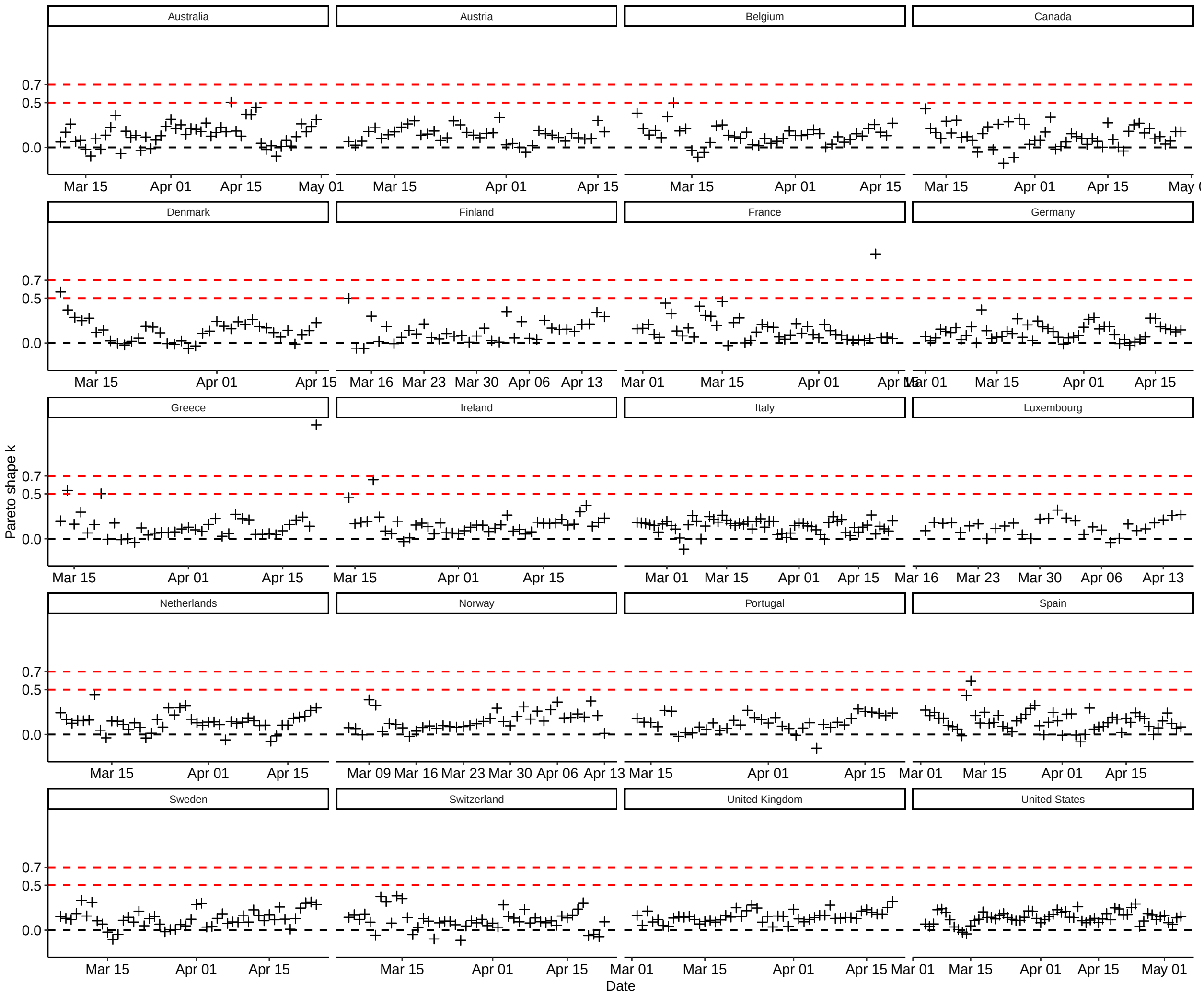

Fig 7. Model diagnostics based on influential observations. Shown is the tail shape parameter k of the generalized Pareto distribution from approximate leave-one-out cross-validation using Pareto smoothed importance sampling for each observation by country and time. Values below 0.5 indicate that the observation is not influential; values between 0.5 and 0.7 indicate that the observation might be influential but the model is usually still robust; values above 0.7 indicate the observation is influential and that the model may not be robust[21].

6 Sensitivity analysis

6.1 Modeling phase starting from 10 cumulative cases onward

In the main model, the modeling phase starts after 100 cumulative cases were reported by a country. This is because countries needed time to set up documentation practices and thus the early reported cases numbers were very variable. Fig 8 shows the estimated NPI effects when modeling starts after 10 cumulative cases were reported. Overall, NPI effects are not sensitive to a start of the modeling phase after 50 or 100 cumulative cases were observed. The effects of ban of large gatherings, venue closure, and ban of small gatherings are sensitive to a very early start of the modeling phase after 10 cumulative cases, but it should be noted that case numbers were highly variable in the very beginning.

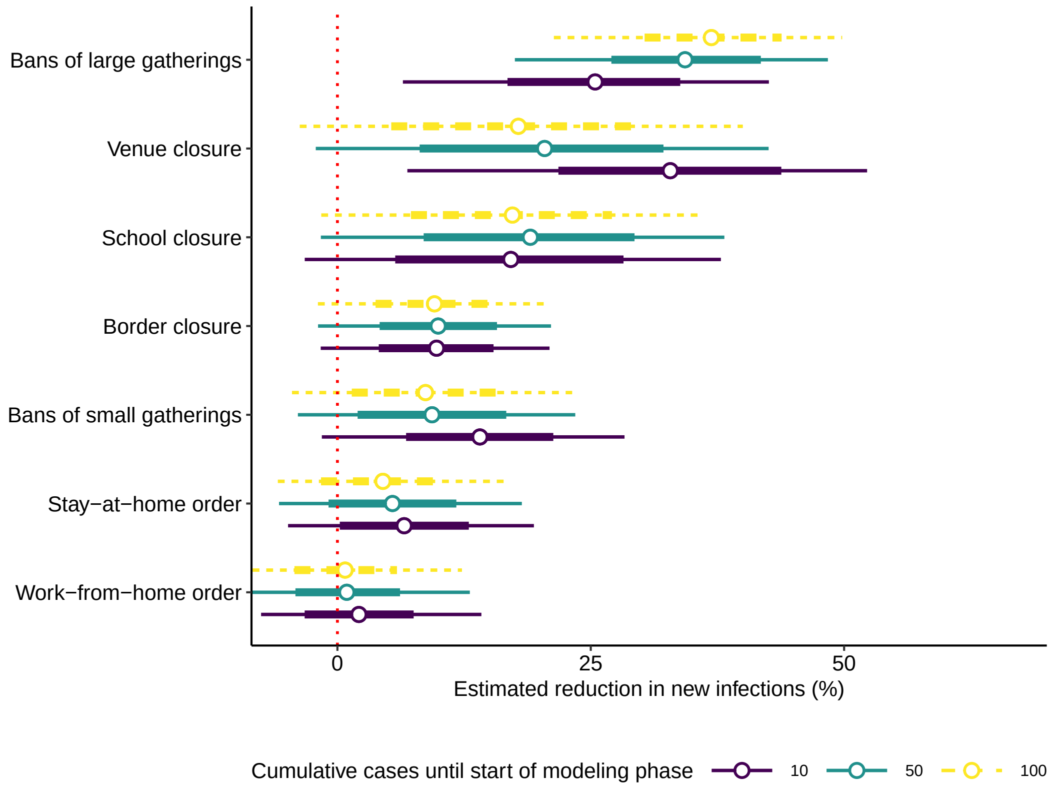

Fig 8. Reduction (posterior mean as dots with 80% and 95% credible interval as thick and thin lines, respectively) in the number of new infections (in %) for each non-pharmaceutical intervention (NPI) when varying the start of the modeling phase (default in main model as dashed yellow line).

6.2 Modeling phase ending 21 to 35 days after last NPI was implemented

In the main model, the modeling phase ends 28 days after the last NPI was implemented. This should provide enough time for the effect of NPIs to show up in the number of reported cases, thereby concluding the first wave of the epidemic. Fig 9 shows the estimated NPI effects when the end of the modeling phase is varied from 21 to 25 days after the last NPI was implemented within a country. NPI effects are a little sensitive to an earlier end of the modeling phase. The wider credible intervals for border closure, ban of small gatherings, stay-at-home order and work-from-home order could indicate that ending the modeling phase 21 days after the last NPI was implemented is a bit too early.

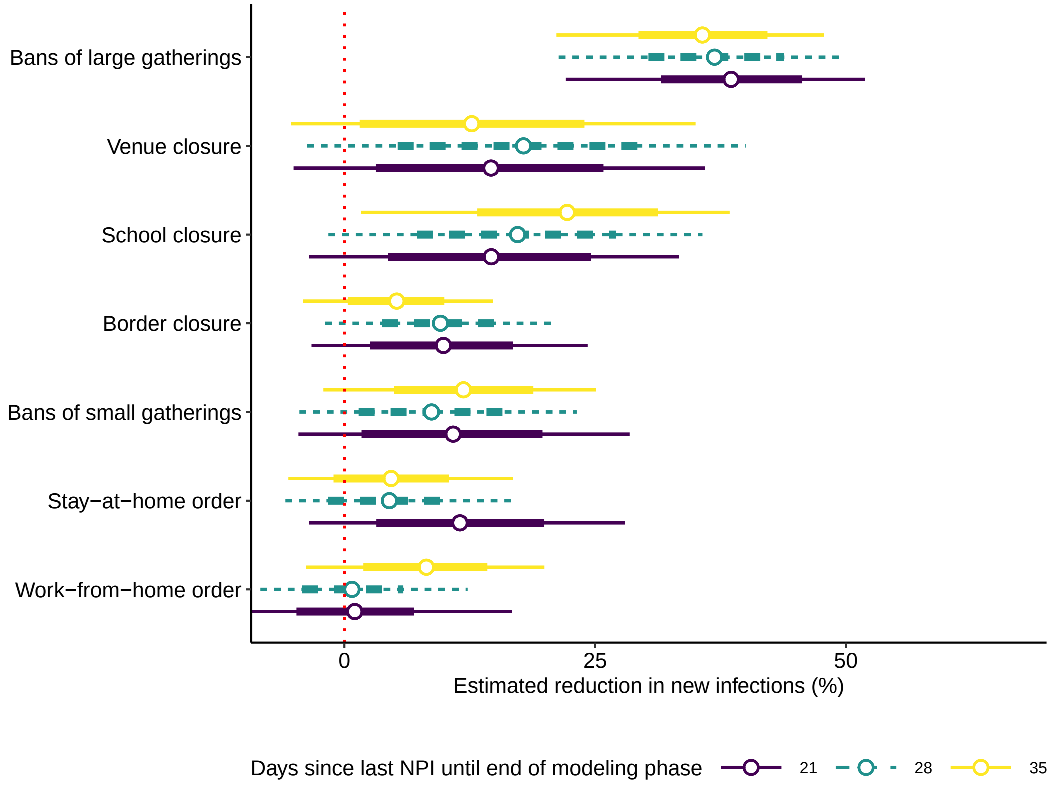

Fig 9. Reduction (posterior mean as dots with 80% and 95% credible interval as thick and thin lines, respectively) in the number of new infections (in %) for each non-pharmaceutical intervention (NPI) when varying the end of the modeling phase (default in main model as dashed turquoise line).

6.3 Varying the time-delayed response functions

In the main model, a time delayed response function was considered where the effect of an NPI increases linearly between $t_0 = 0$ days and $t_1 = 3$ days after their implementation. Fig 10 shows the estimated NPI effects when varying t_0 and t_1 considering the following alternatives for the first-order spline FOS(t_0,t_1): a proactive response FOS(−1,0), a quicker response FOS(0,1), and a more delayed response FOS(0,5) as compared to the main model. Overall, the results are not sensitive to the choice of t_0 and t_1 in the FOS.

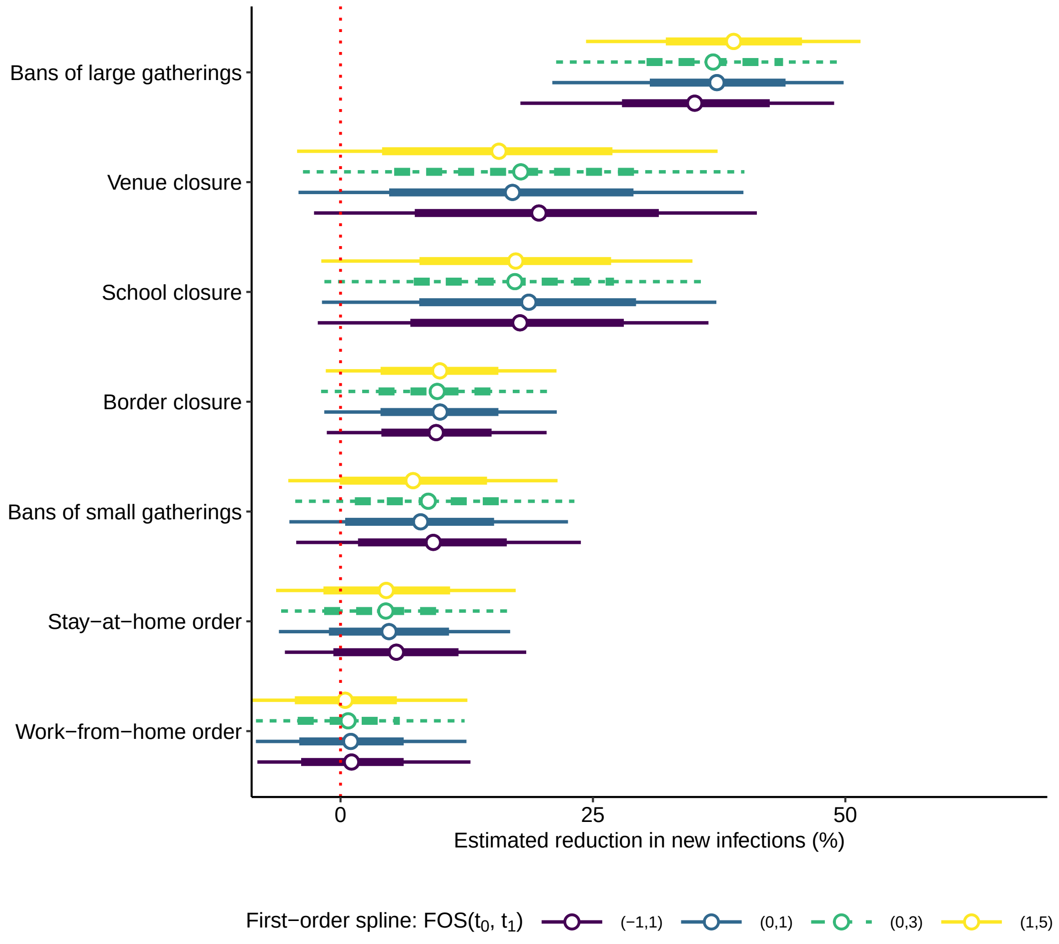

Fig 10. Reduction (posterior mean as dots with 80% and 95% credible interval as thick and thin lines, respectively) in the number of new infections (in %) for each non-pharmaceutical intervention (NPI) when varying time delayed response function (default in main model as dashed turquoise line).

6.4 Varying the prior distribution for the effects of non-pharmaceutical interventions

In the main model, a mixture prior for the NPI effects was constructed where the probability of a negative effect (i.e., NPIs leading to an increase in the number of new cases) is 10 %. Fig 11 shows the estimated NPI effects when the prior probability of a negative effect of NPIs is alternatively 30 % or 50 %. The ranking of the posterior mean effects does not depend on the prior, but the range of effects for venue closure and work-from-home order would include larger negative effects when increasing the prior probability for a negative effect.

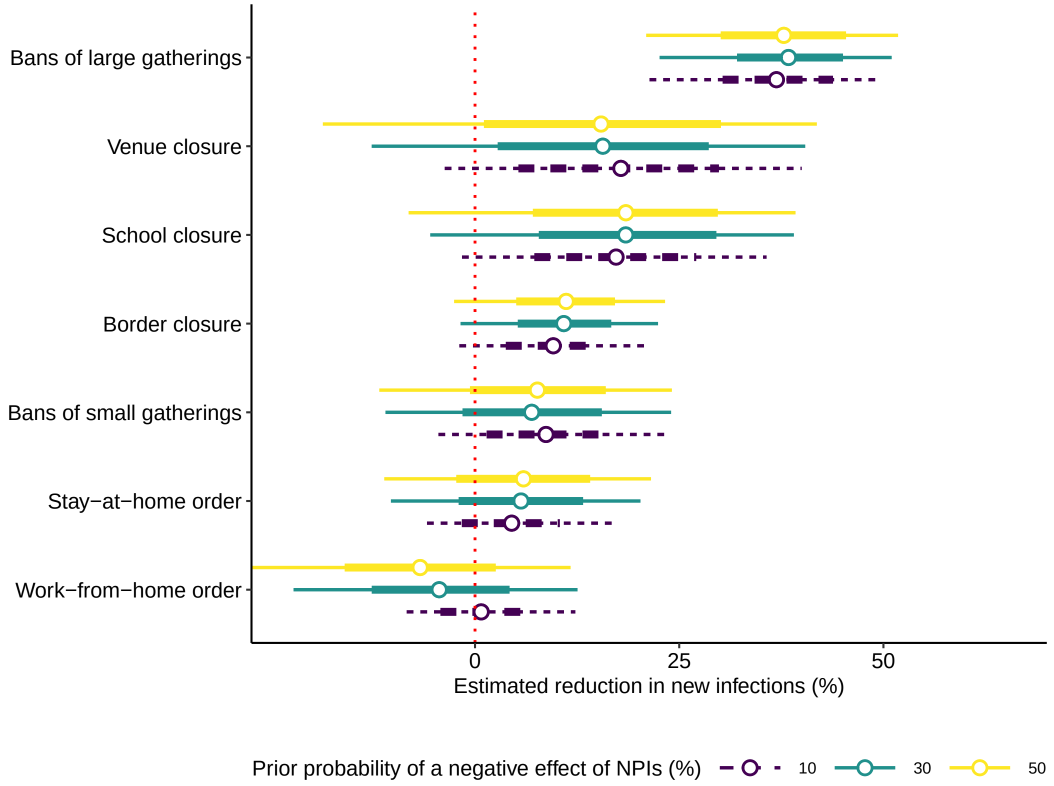

Fig 11. Reduction (posterior mean as dots with 80% and 95% credible interval as thick and thin lines, respectively) in the number of new infections (in %) for each non-pharmaceutical intervention (NPI) when varying the probability of a negative effect (in %) in the prior distribution for the effects of non-pharmaceutical interventions (default in main model as dashed purple line).

6.5 Varying the prior distribution for the time from infection to reporting of a new case

In the main model, the probability distribution for the time from infection to reporting of a new case $p_{\mathrm{IN}}(t)$ was inferred by estimating the log mean μ and the log standard deviation σ of the assumed lognormal distribution. The specified priors in the main model correspond to a Normal($\mu_0 = 2.47, \sigma_0 = 0.45$) for the log mean and Gamma($\alpha_0 = 2, = \beta_0 = 4$) for the log standard deviation, such that $\mu = \mu_0 = 2.47$ and $\sigma = \frac{\alpha_0}{\beta_0} = \frac{2.00}{4.48} \approx 0.45$ correspond to the prior means of a Lognormal($\mu = 2.47, \sigma = 0.45$) that was obtained based on prior knowledge. For this sensitivity check, μ_0 and β_0 were varied in the Normal prior for the log mean μ and the Gamma prior for the log standard deviation σ, resulting in alternative probability distributions for $p_{\mathrm{IN}}(t)$ (Fig 12). Fig 13 shows the estimated NPI effects for this sensitivity check. Overall, NPI effects are not sensitive to the choice of priors for the parameters of $p_{\mathrm{IN}}(t)$.

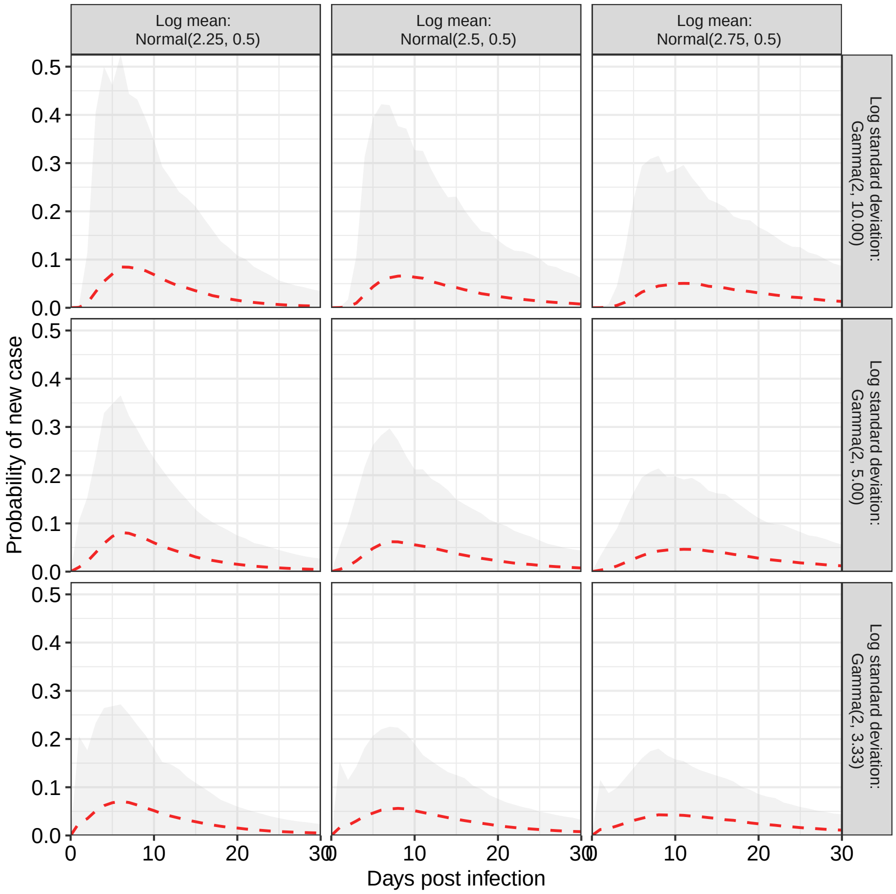

Fig 12. Prior choices for the distribution of the time from infection to reporting of a new case $p_{\mathrm{IN}}(t)$ depending on log mean μ and log standard deviation σ (prior mean as dashed red line with 95 % range as shaded area, based on 4,000 independent draws from the distributions for the log mean μ (column) in the sensitivity analysis (default in main model as middle tile).

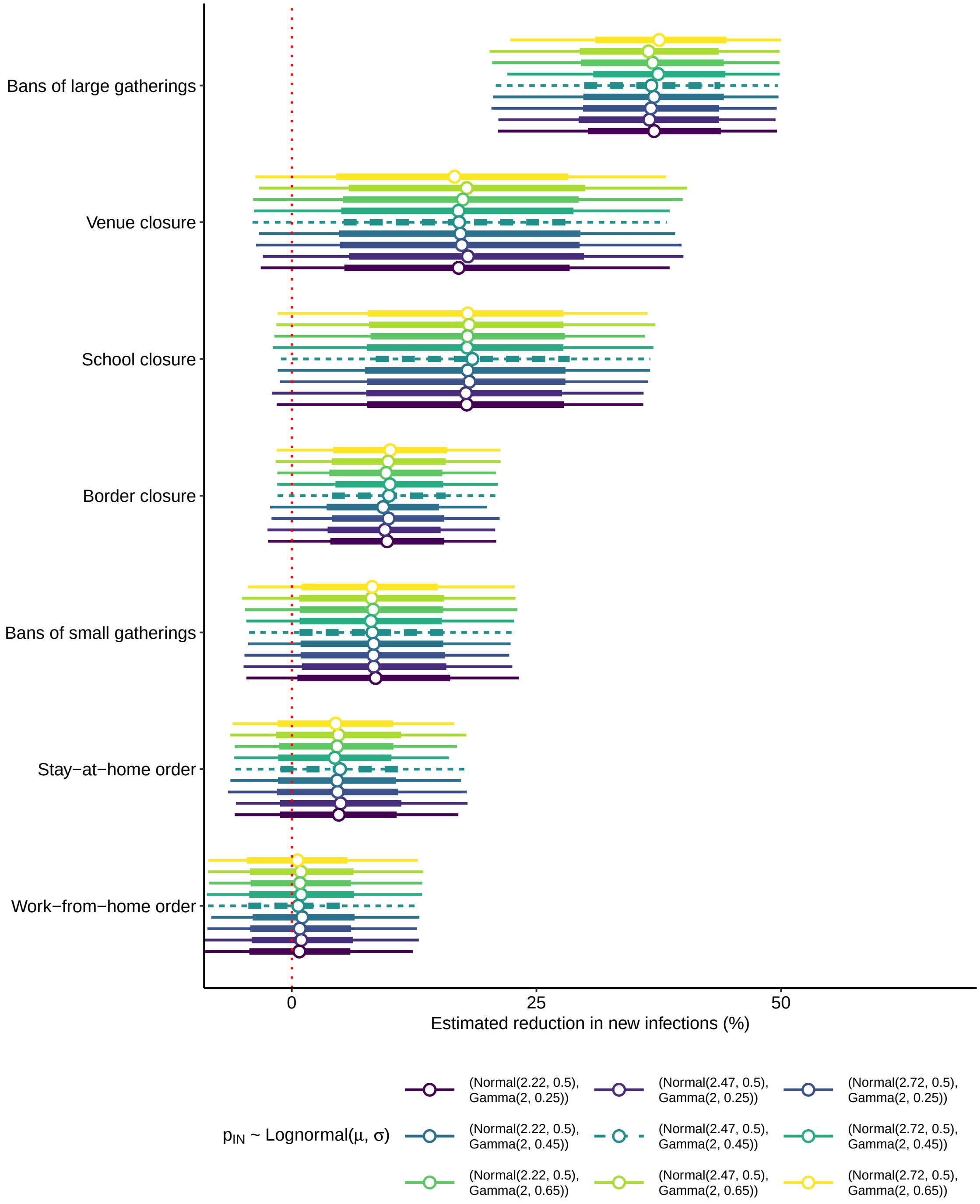

Fig 13. Reduction (posterior mean as dots with 80% and 95% credible interval as thick and thin lines, respectively) in the number of new infections (in %) for each non-pharmaceutical intervention (NPI) when varying the prior choices for distribution of the time from infection to reporting of a new case (default in main model as dashed turquoise line).

6.6 Varying the generation time distribution

In the main model, the generation time distribution $p_G(t)$ was assumed to be a Weibull($\alpha = 3.28, \beta = 6.12$)[13]. For this sensitivity check, the shape parameter α and inverse scale parameter β were varied, resulting in the alternative probability distributions for $p_G(t)$ (Fig 14). Fig 15 shows the estimated NPI effects for this sensitivity check. Overall, NPI effects are not very sensitive to the choice of $p_G(t)$, except that the estimated effects tend to increase for longer generation times.

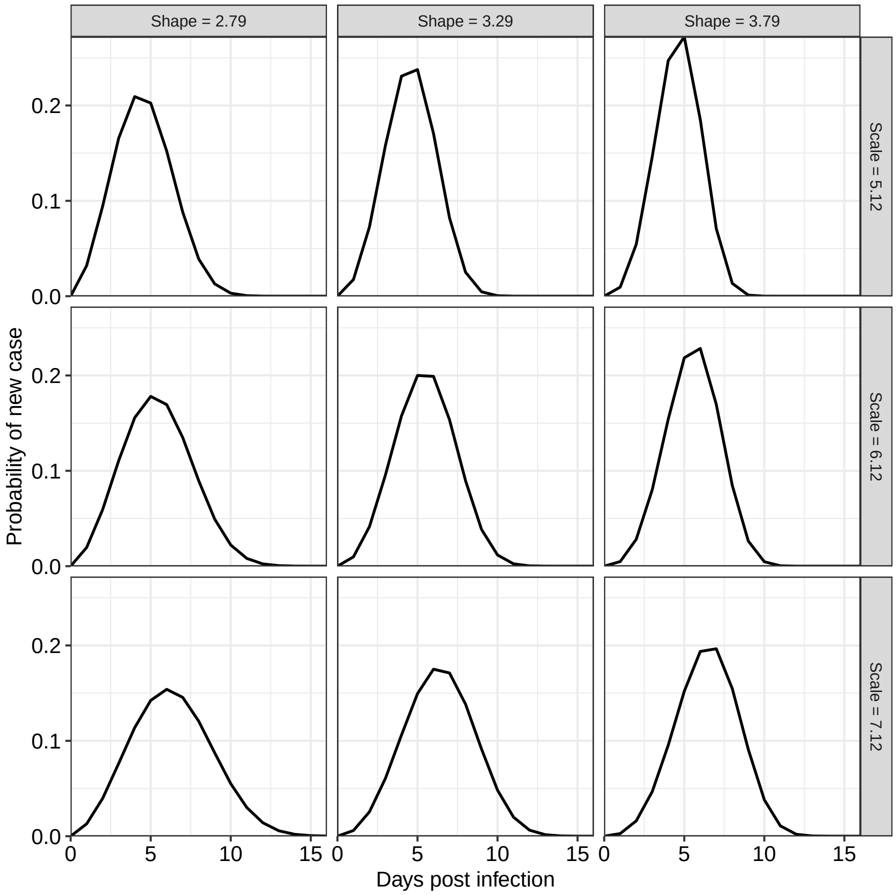

Fig 14. Prior choices for the generation time distribution $p_G(t)$ depending on shape parameter α and rate parameter κ in the sensitivity analysis (default in main model as middle tile).

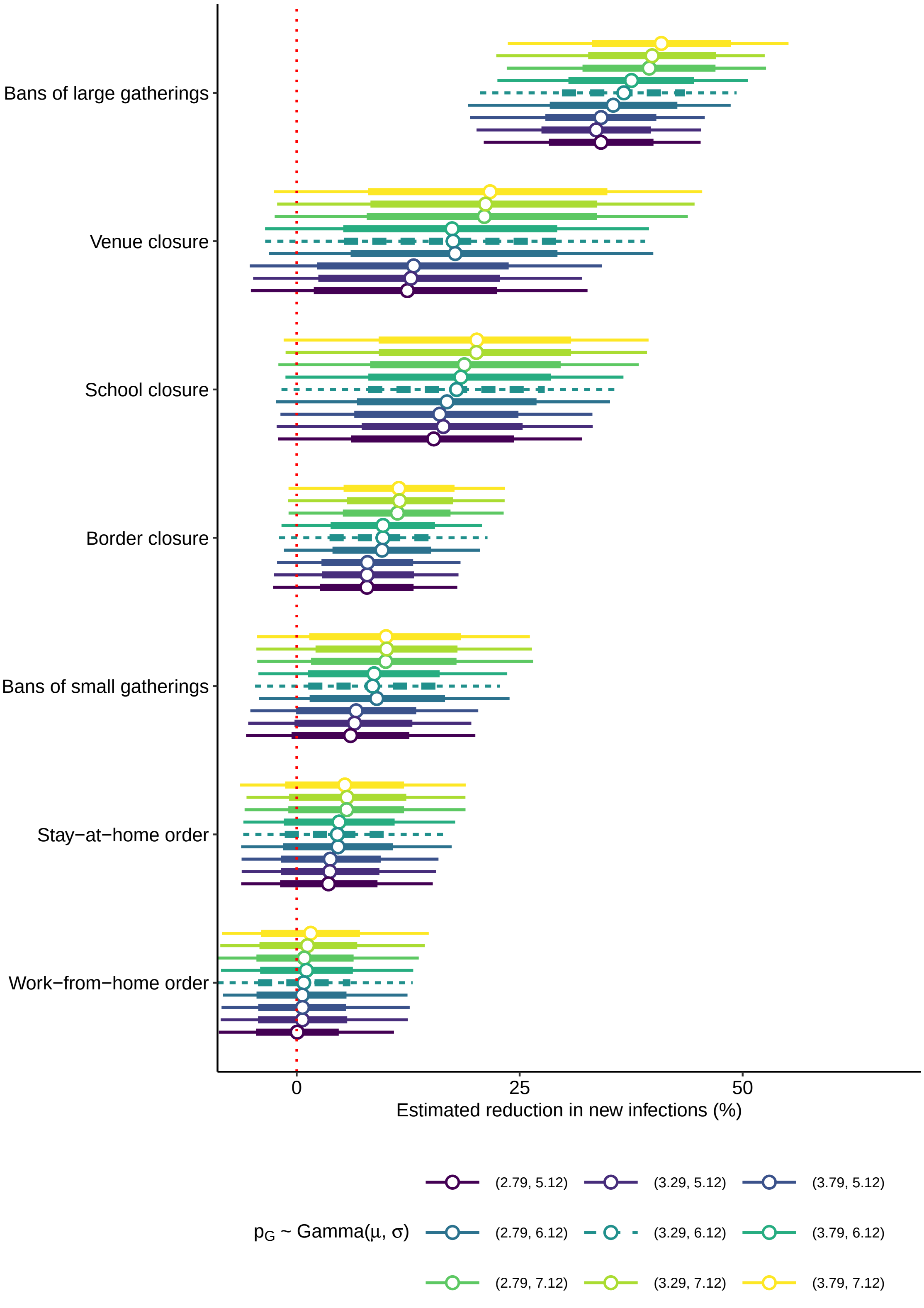

Fig 15. Reduction (posterior mean as dots with 80% and 95% credible interval as thick and thin lines, respectively) in the number of new infections (in %) for each non-pharmaceutical intervention (NPI) when varying the generation time distribution (default in main model turquoise as dashed line).

6.7 Analyzing the influence of leaving out one country at the time

A leave-one-out analysis was conducted to analyze the influence of individual countries for the estimated NPI effects, i.e., the model is re-estimated leaving out one country at a time. Fig 16 shows the results of this analysis for each NPI. Some estimated effects seem sensitive to the exclusion of individual countries.

The effect of school closure would be higher when estimating the model without Australia. This could implicate sensitivity or, instead, it could be that the particular country is informative for the estimated effect of a particular NPI. Australia is a good example to check for this, as here, school closures where implemented in Queensland, New South Wales, Victoria and Australian Capital Territory (Eastern Australia) but not in Western Australia, Northern Territory and South Australia (Western Australia). As a result, Australia was split into Eastern and Western Australia and the model was estimated leaving out one region at a time. This time, the effect of school closure is lower without Eastern Australia and higher without Western Australia (Fig 17). A reason for this could be that both regions successfully reduced the number of cases, but Western Australia did so with similar measures as Eastern Australia except for closing schools, thereby providing substantial evidence against the particular effectiveness of school closures.

The effect of bans of large gatherings would be higher without Switzerland and Sweden. Note that both countries implemented bans of large gatherings comparably early into the epidemic. Despite that, the number of new infections was still increasing in Switzerland for a couple of weeks and continuously in Sweden, thereby indicating that bans of large gatherings were potentially not as effective as in other countries.

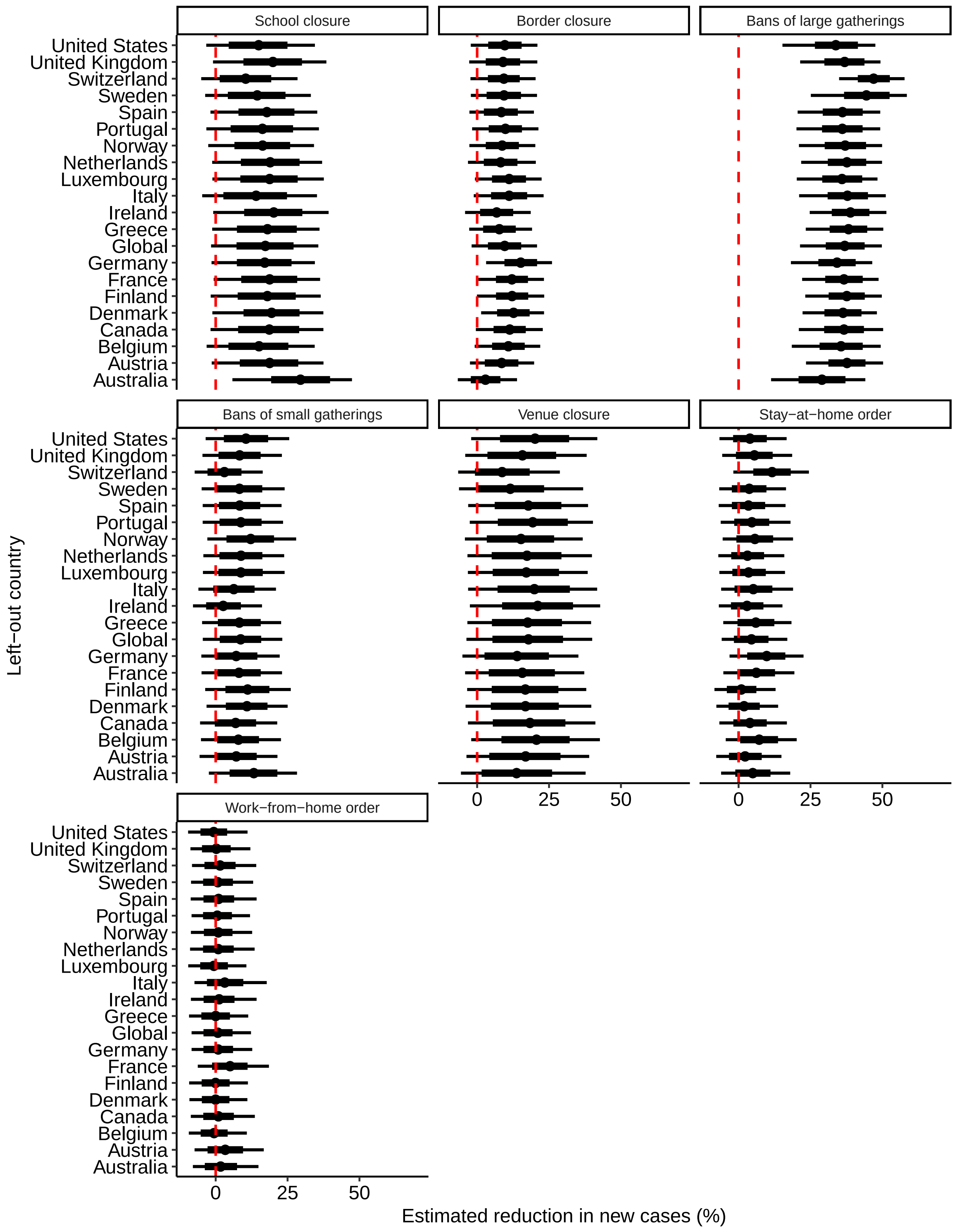

Fig 16. Reduction (posterior mean as dots with 80% and 95% credible interval as thick and thin lines, respectively) in the number of new infections (in %) for each non-pharmaceutical intervention (NPI) when leaving out one country at a time.

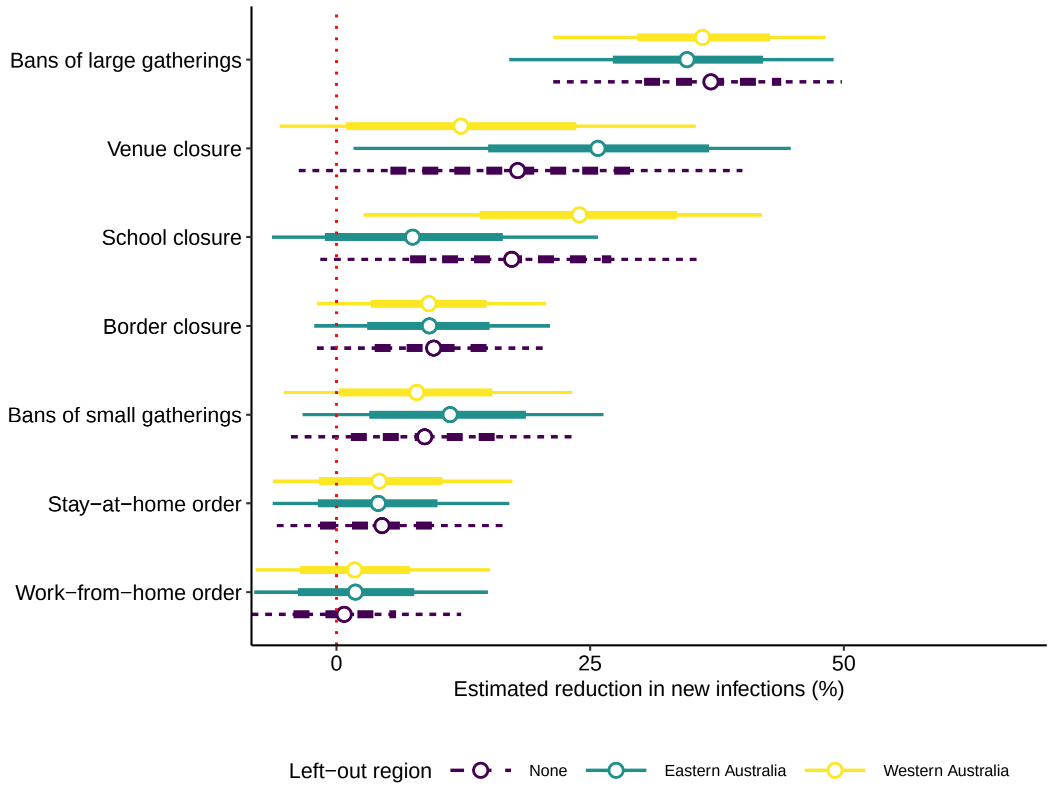

Fig 17. Reduction (posterior mean as dots with 80% and 95% credible interval as thick and thin lines, respectively) in the number of new infections (in %) for each non-pharmaceutical intervention (NPI) when dividing Australia into two sub-regions and leaving-out Eastern or Western Australia (default in main model as dashed line).

7 Visual inspection of the model fit

Fig 18 shows the expected number of new infections (μ^I) and new cases (μ^N) over time for each country. The estimated numbers are compared to the observed number of new cases in order to assess the model fit. Overall, our model provides a reasonable fit in the sense that the expected number of new cases follow the development of the observed number of new cases in each country. Furthermore, changes in the expected number of new infections clearly follow the implementation of NPIs. The size of the credible intervals reflect varying uncertainty in the expected number of new infections and cases in each country (e.g., compare Germany (small) to France (large)). For a couple of countries (e.g., Ireland, Sweden, United Kingdom, United States), the credible intervals are particularly large at the very end of the epidemic. This corresponds to a rather high level of new infections at that time, indicating that the interventions were not yet enough to strongly reduce the number of new infections. The large credible intervals are a reminder that this implies a risk for a new exponential increase in the number of new infections.

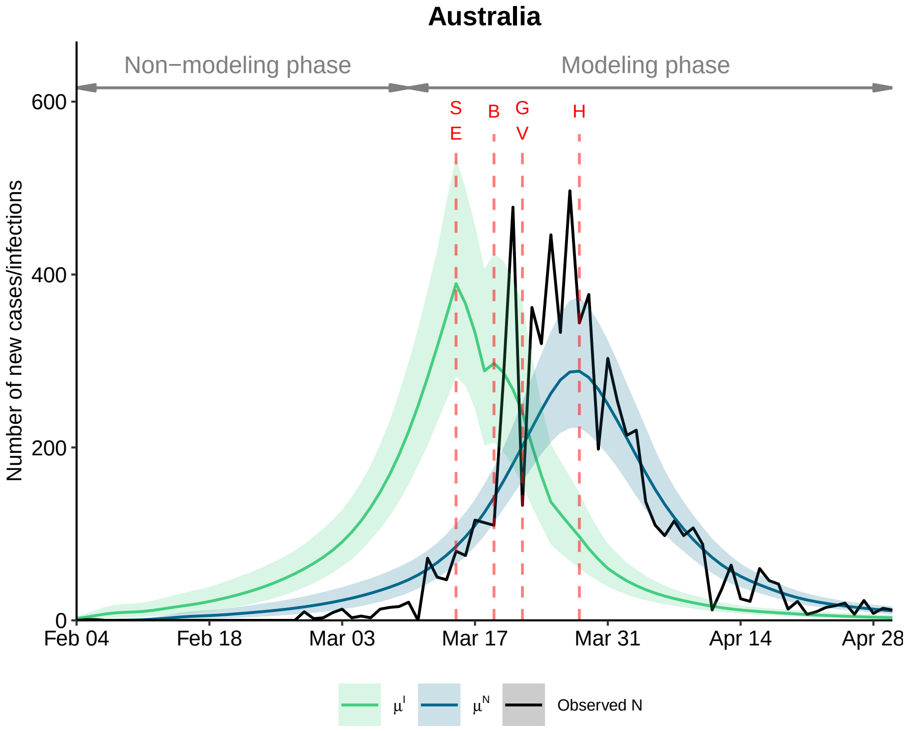

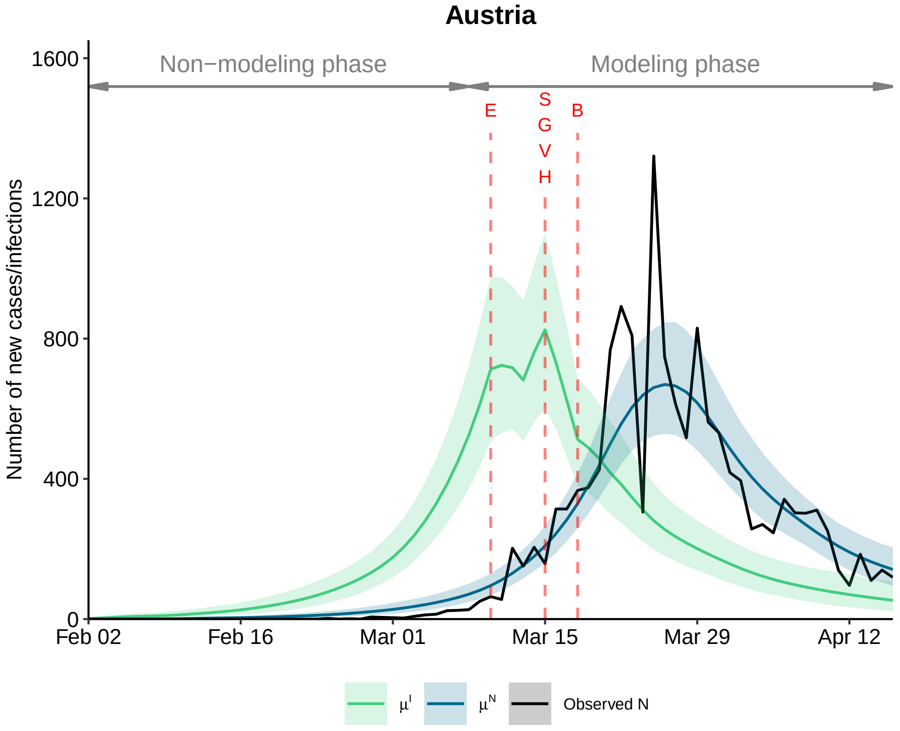

Fig 18. Expected number of new infections μ^I and new cases μ^N (posterior mean as colored lines with 95% credible interval as shaded area) and the observed number of new cases by country over time. Red letters and lines indicate the first day an NPI was implemented within a country (S: School closures, B: Border closure, E: Ban of large gatherings, G: Ban of small gatherings, V: Venue closure, H: Stay-at-home order, W: Work-from-home order).

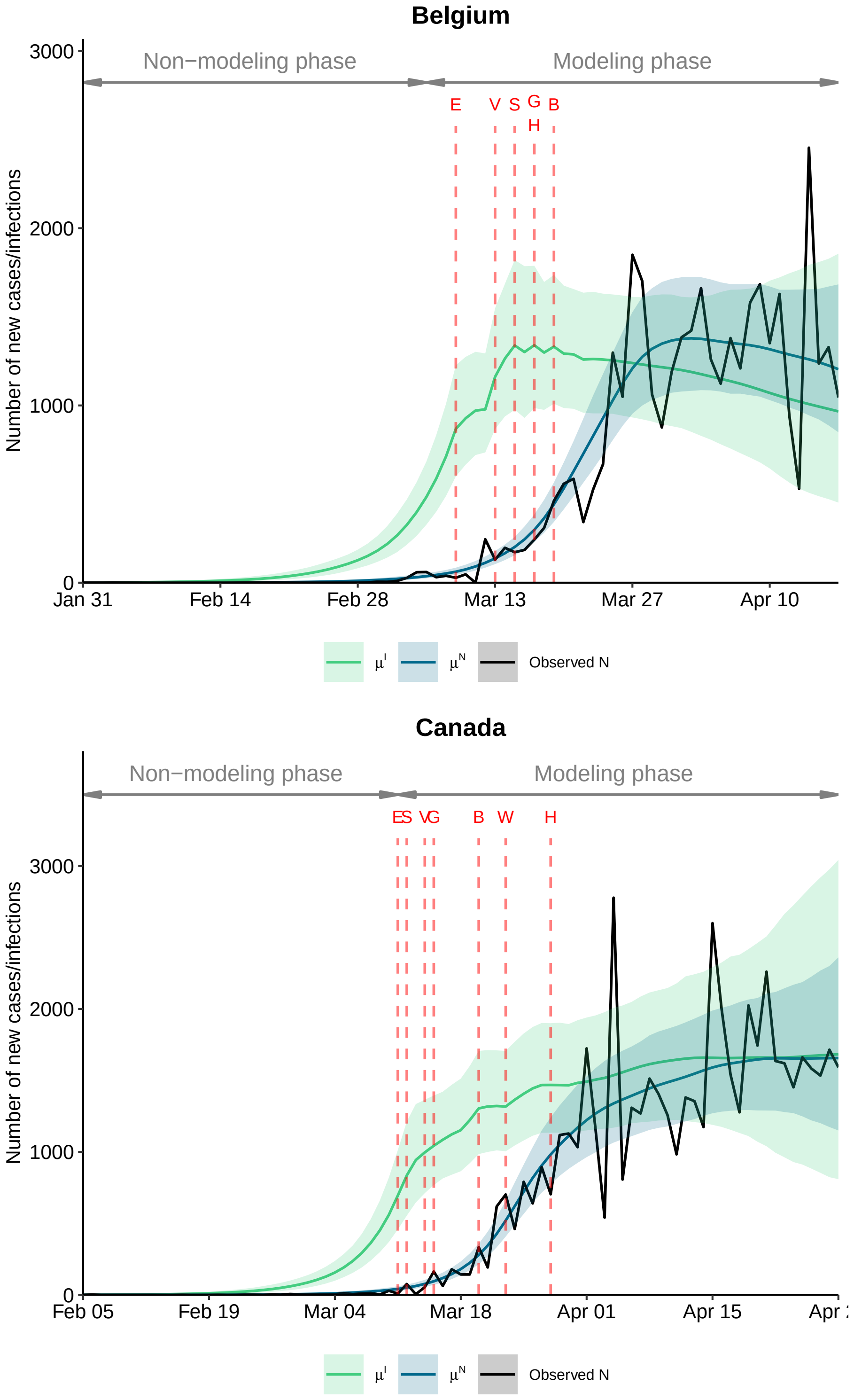

Fig 18. Expected number of new infections μ^I and new cases μ^N (posterior mean as colored lines with 95% credible interval as shaded area) and the observed number of new cases by country over time. Red letters and lines indicate the first day an NPI was implemented within a country (S: School closures, B: Border closure, E: Ban of large gatherings, G: Ban of small gatherings, V: Venue closure, H: Stay-at-home order, W: Work-from-home order).

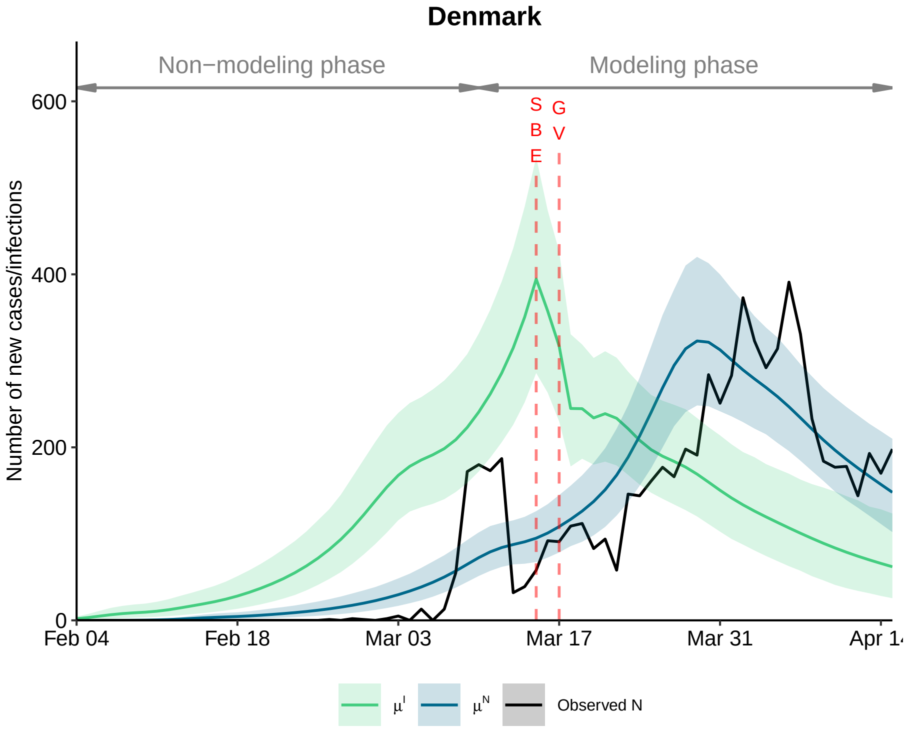

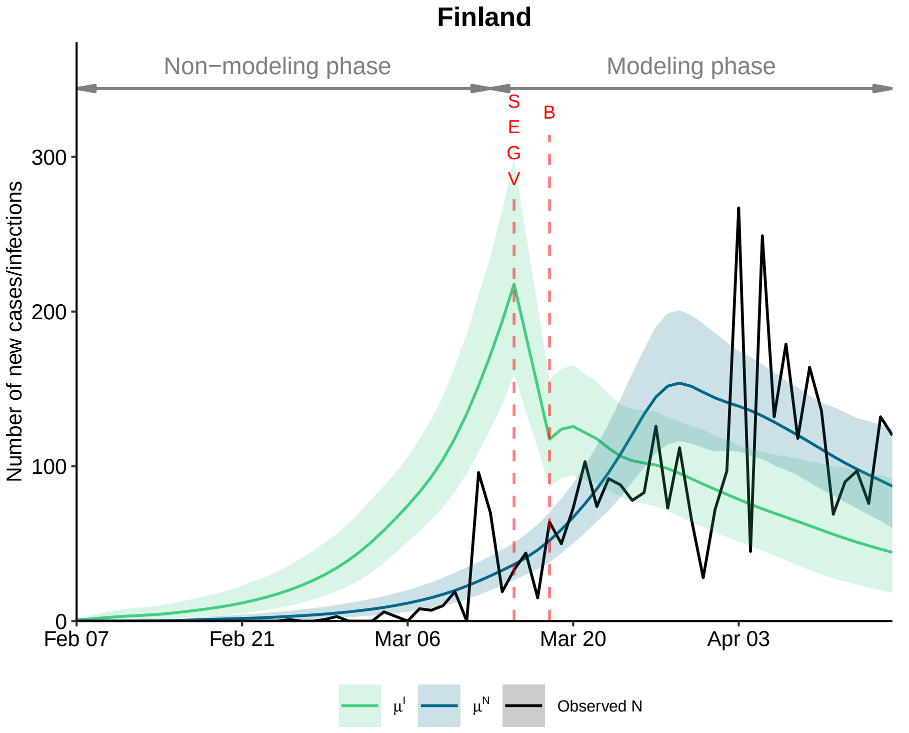

Fig 18. Expected number of new infections μ^I and new cases μ^N (posterior mean as colored lines with 95% credible interval as shaded area) and the observed number of new cases by country over time. Red letters and lines indicate the first day an NPI was implemented within a country (S: School closures, B: Border closure, E: Ban of large gatherings, G: Ban of small gatherings, V: Venue closure, H: Stay-at-home order, W: Work-from-home order).

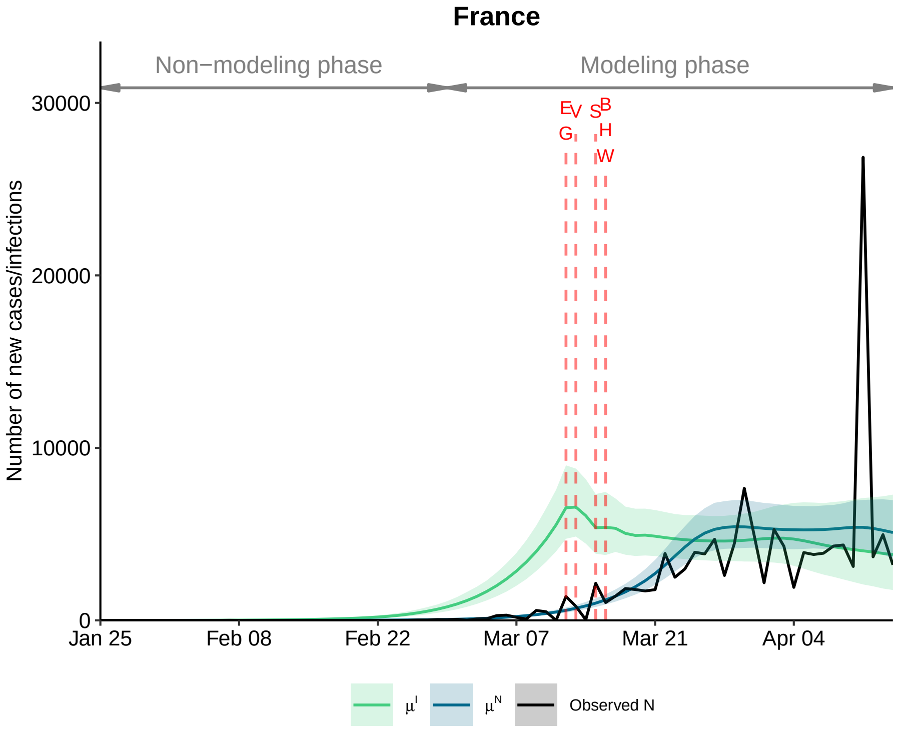

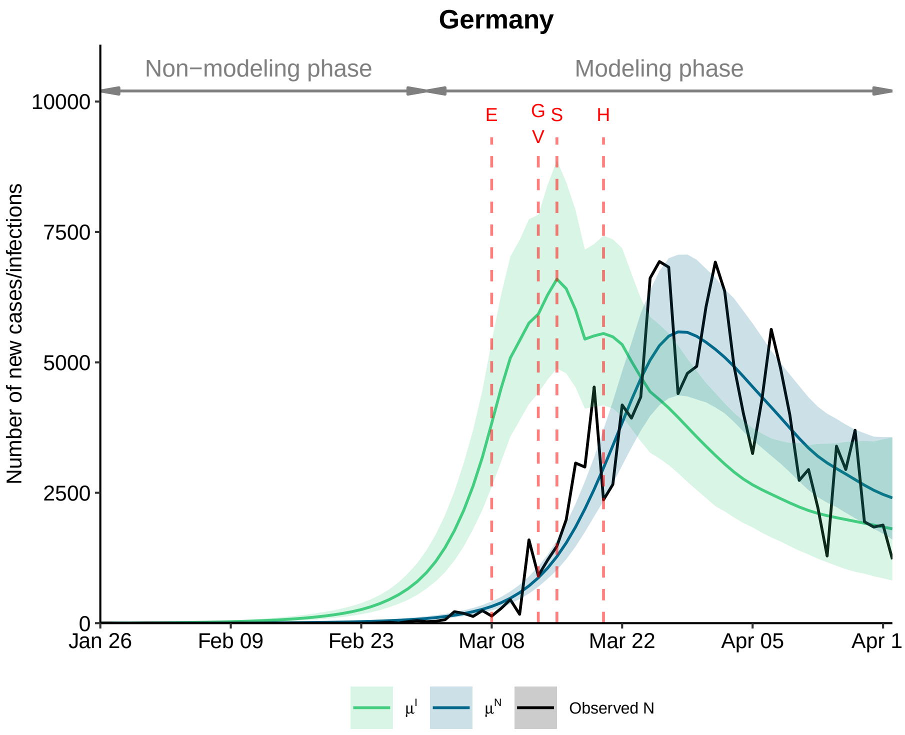

Fig 18. Expected number of new infections μ^I and new cases μ^N (posterior mean as colored lines with 95% credible interval as shaded area) and the observed number of new cases by country over time. Red letters and lines indicate the first day an NPI was implemented within a country (S: School closures, B: Border closure, E: Ban of large gatherings, G: Ban of small gatherings, V: Venue closure, H: Stay-at-home order, W: Work-from-home order).

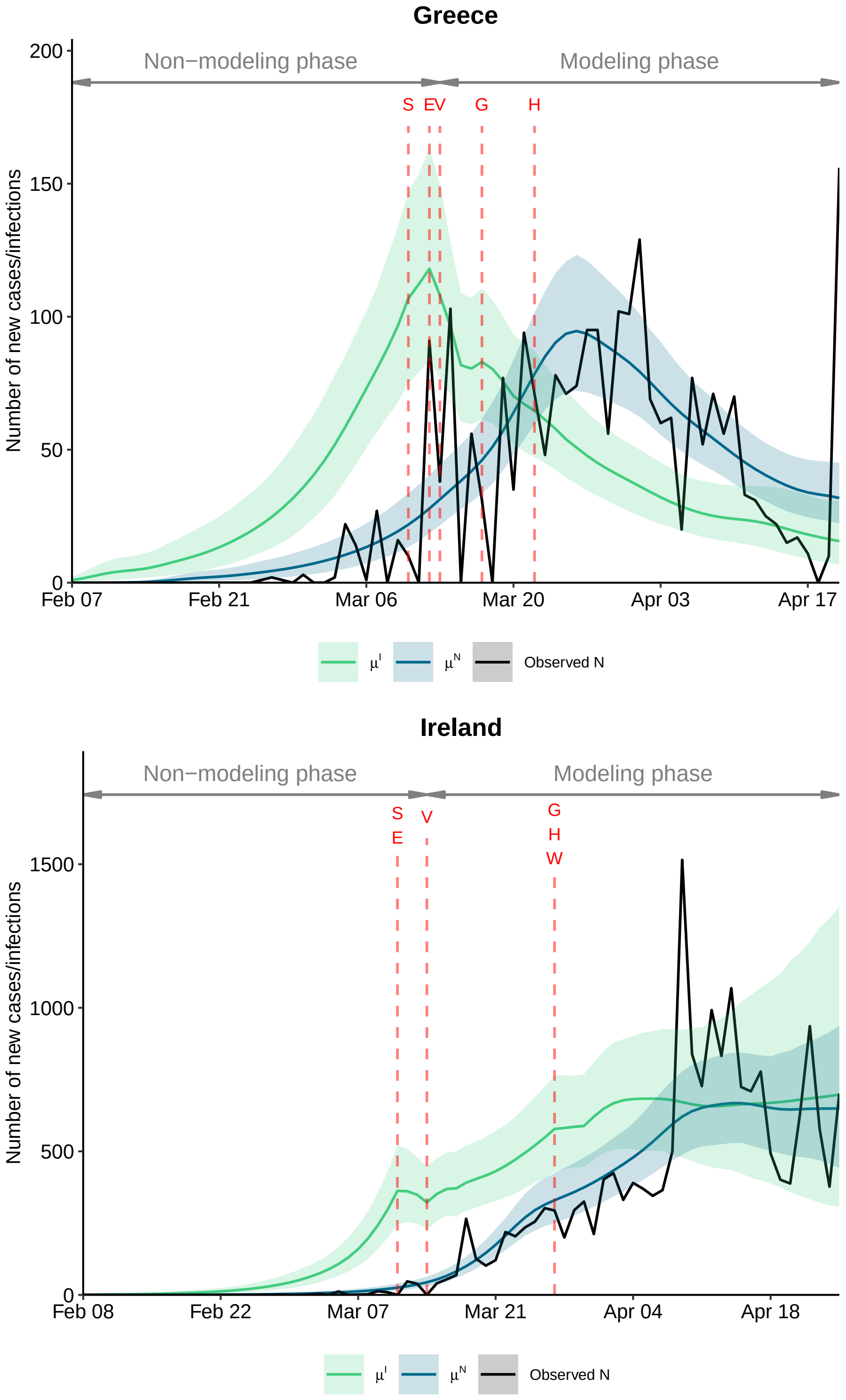

Fig 18. Expected number of new infections μ^I and new cases μ^N (posterior mean as colored lines with 95% credible interval as shaded area) and the observed number of new cases by country over time. Red letters and lines indicate the first day an NPI was implemented within a country (S: School closures, B: Border closure, E: Ban of large gatherings, G: Ban of small gatherings, V: Venue closure, H: Stay-at-home order, W: Work-from-home order).

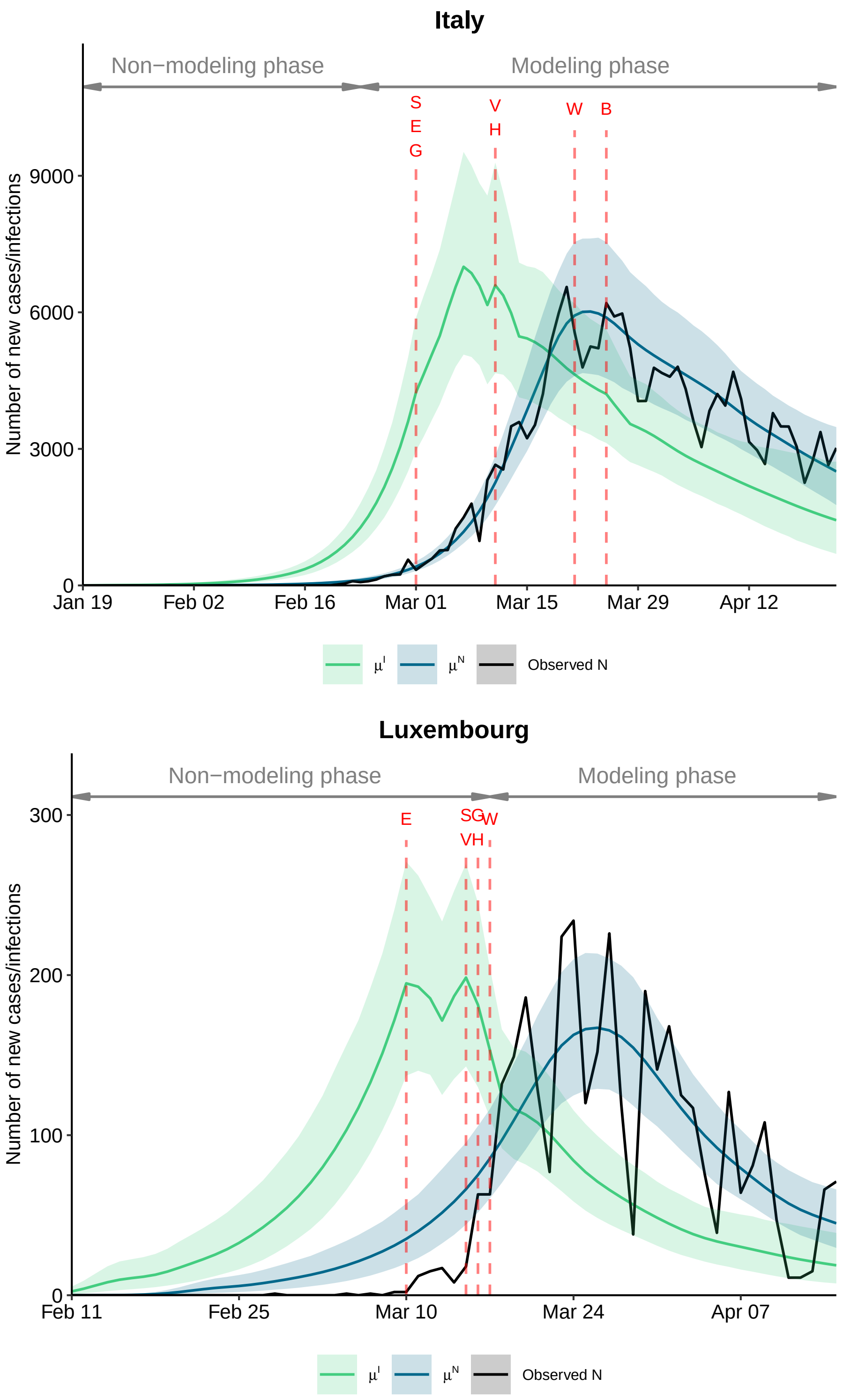

Fig 18. Expected number of new infections μ^I and new cases μ^N (posterior mean as colored lines with 95% credible interval as shaded area) and the observed number of new cases by country over time. Red letters and lines indicate the first day an NPI was implemented within a country (S: School closures, B: Border closure, E: Ban of large gatherings, G: Ban of small gatherings, V: Venue closure, H: Stay-at-home order, W: Work-from-home order).

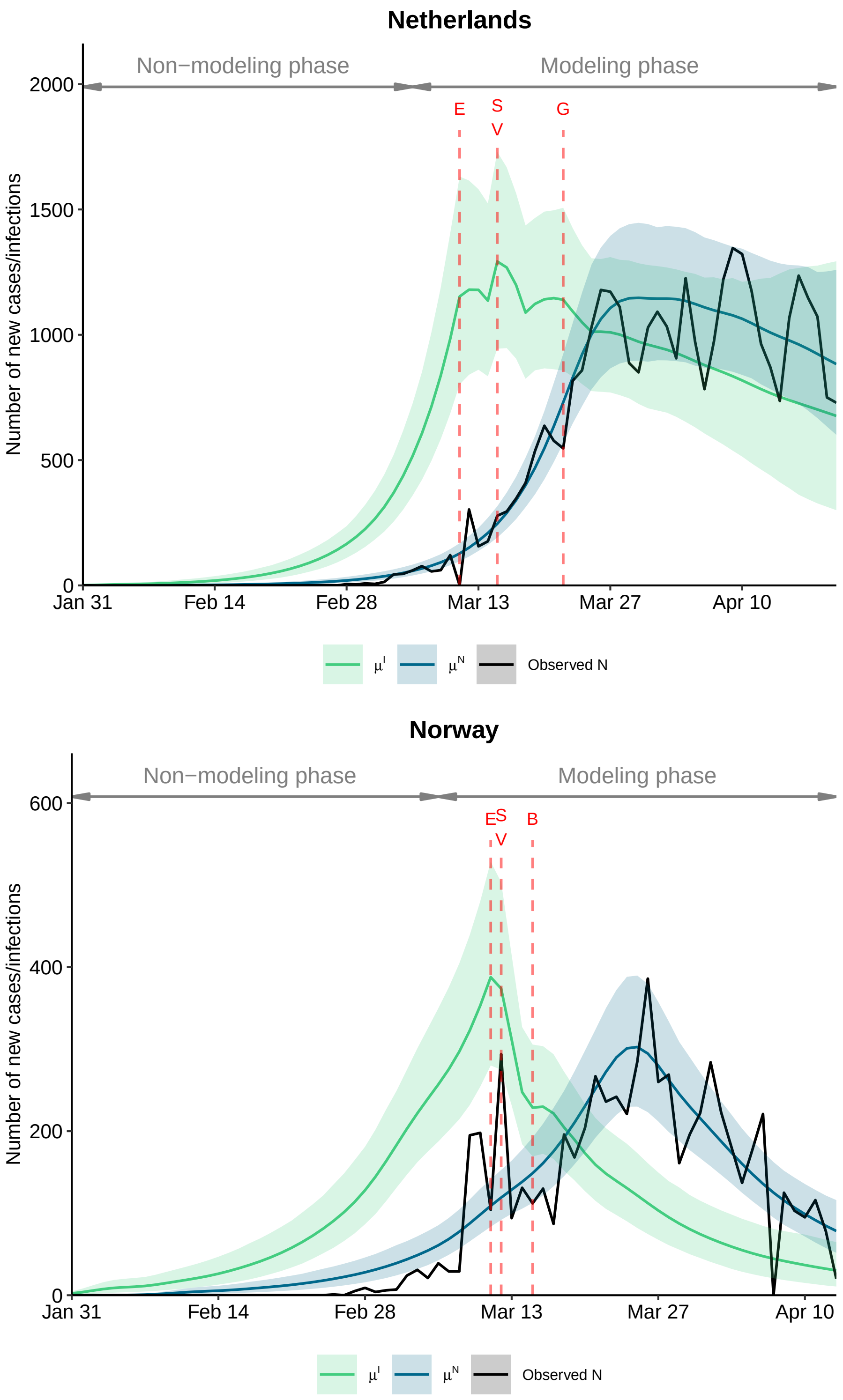

Fig 18. Expected number of new infections μ^I and new cases μ^N (posterior mean as colored lines with 95% credible interval as shaded area) and the observed number of new cases by country over time. Red letters and lines indicate the first day an NPI was implemented within a country (S: School closures, B: Border closure, E: Ban of large gatherings, G: Ban of small gatherings, V: Venue closure, H: Stay-at-home order, W: Work-from-home order).

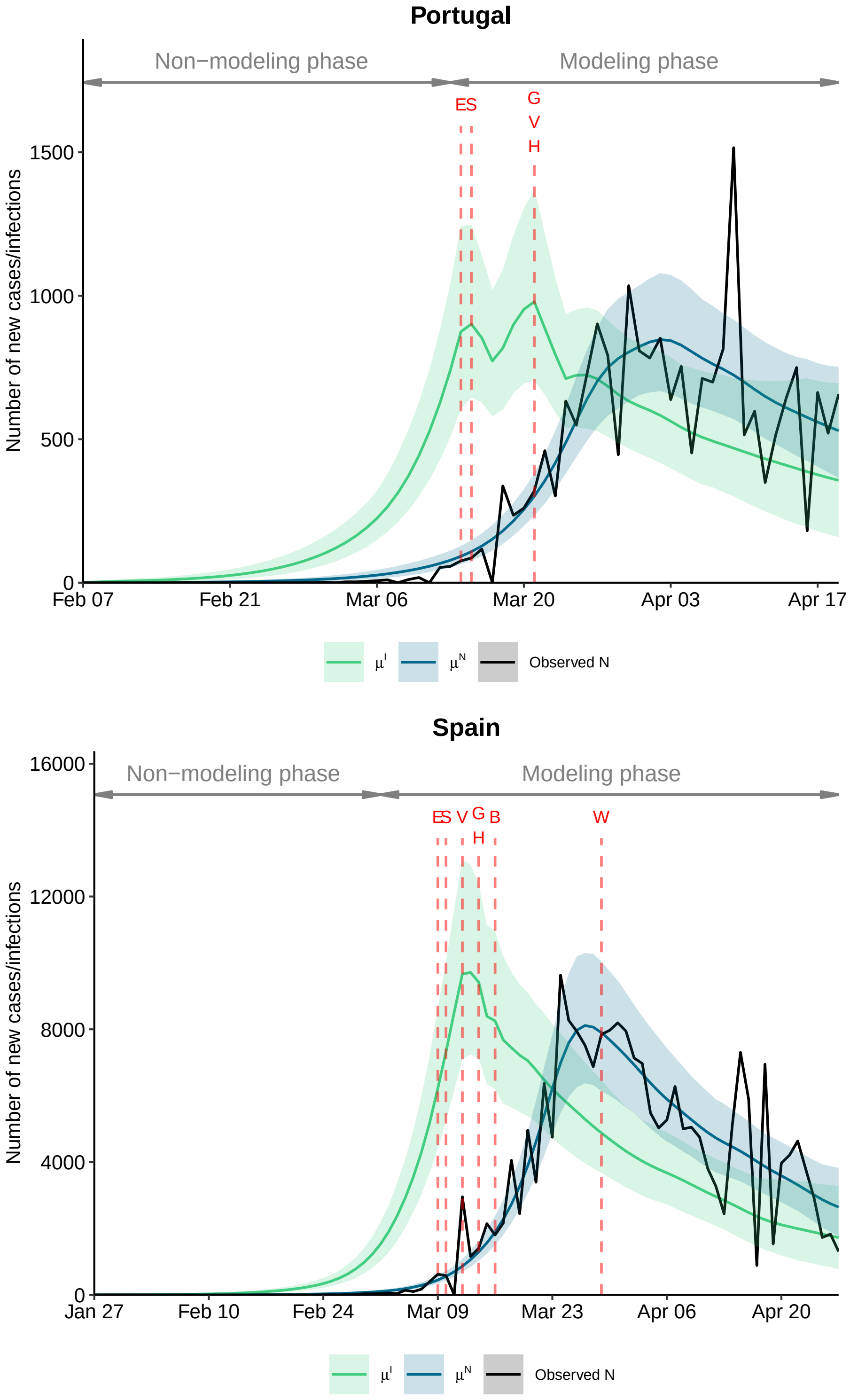

Fig 18. Expected number of new infections μ^I and new cases μ^N (posterior mean as colored lines with 95% credible interval as shaded area) and the observed number of new cases by country over time. Red letters and lines indicate the first day an NPI was implemented within a country (S: School closures, B: Border closure, E: Ban of large gatherings, G: Ban of small gatherings, V: Venue closure, H: Stay-at-home order, W: Work-from-home order).

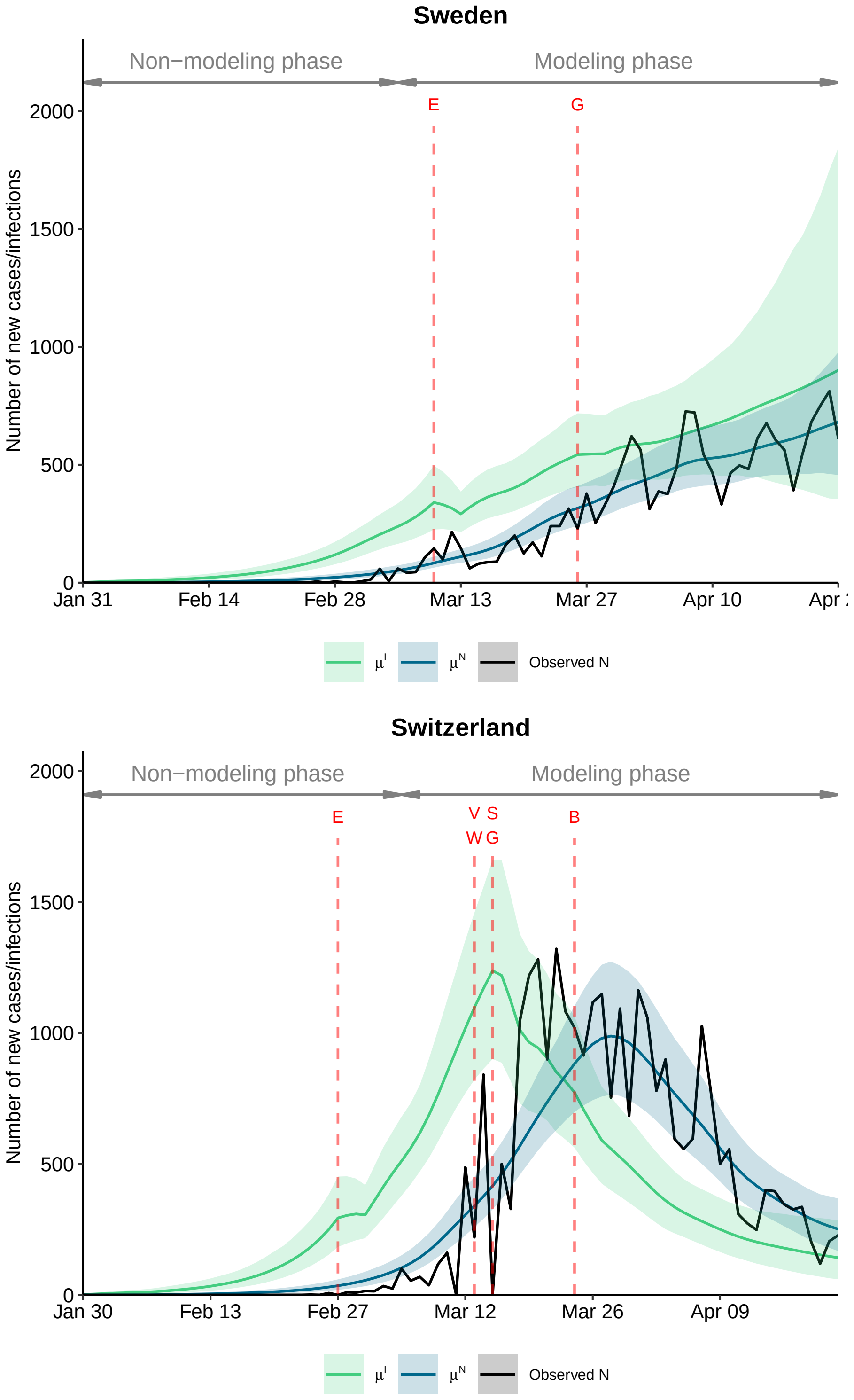

Fig 18. Expected number of new infections μ^I and new cases μ^N (posterior mean as colored lines with 95% credible interval as shaded area) and the observed number of new cases by country over time. Red letters and lines indicate the first day an NPI was implemented within a country (S: School closures, B: Border closure, E: Ban of large gatherings, G: Ban of small gatherings, V: Venue closure, H: Stay-at-home order, W: Work-from-home order).

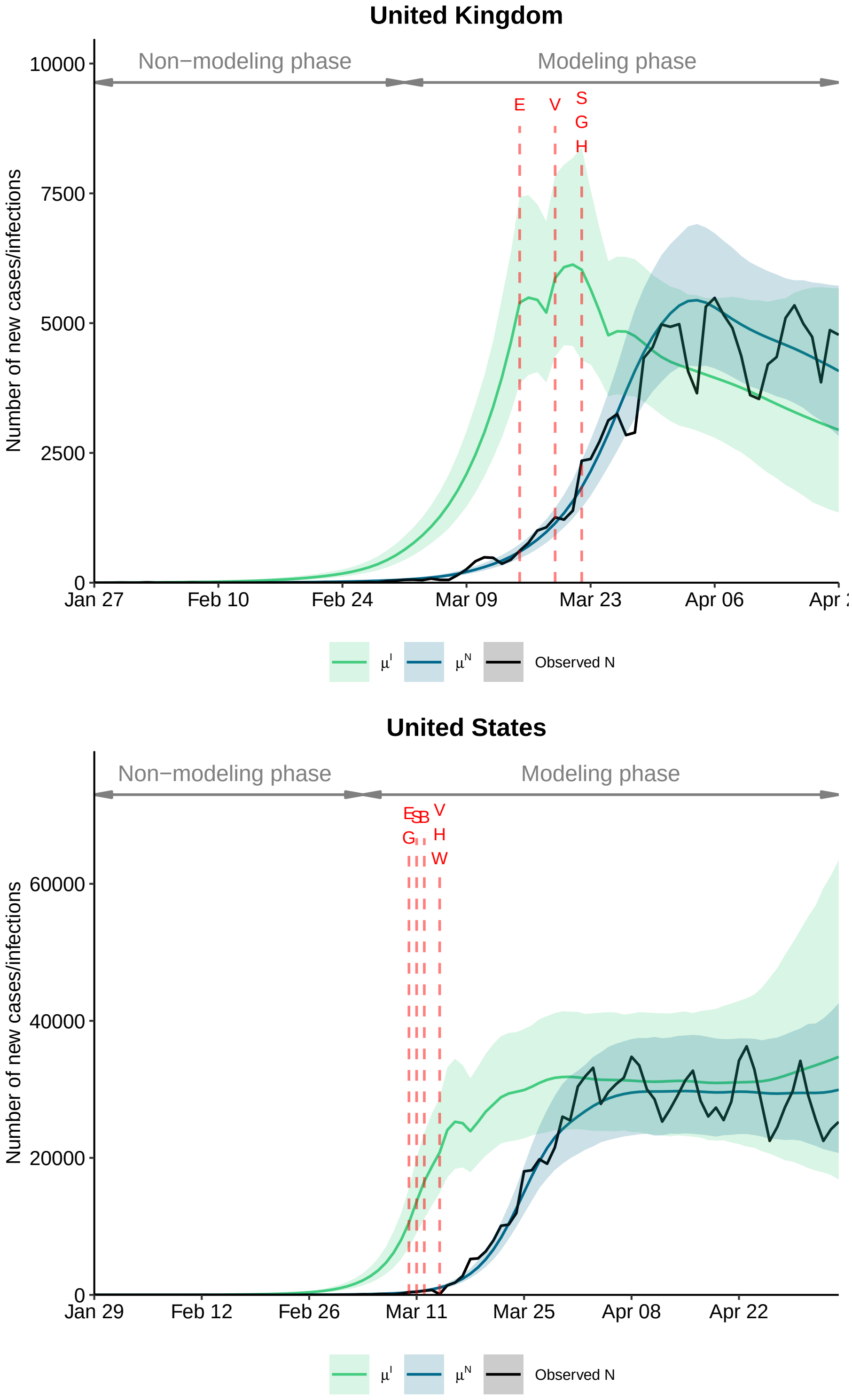

Fig 18. Expected number of new infections μ^I and new cases μ^N (posterior mean as colored lines with 95% credible interval as shaded area) and the observed number of new cases by country over time. Red letters and lines indicate the first day an NPI was implemented within a country (S: School closures, B: Border closure, E: Ban of large gatherings, G: Ban of small gatherings, V: Venue closure, H: Stay-at-home order, W: Work-from-home order).

8 Data on non-pharmaceutical interventions

8.1 Data collection

Data on non-pharmaceutical interventions (NPIs) have been collected systematically in six steps.

1. Granular information on NPIs have been gathered from government resources and news outlets by two authors (AC, PB).

2. After collecting data for the first few countries, NPIs have been classified into seven categories by seven authors (NB, EvW, AL, AS, DT, AC, PB): (1) school closures, (2) border closures, (3) bans of large gatherings, (4) bans of small gatherings, (5) venue closures (e.g., shops, bars, restaurants, and other recreational activities), (6) stay-at-home orders prohibiting public movements without valid reason, and (7) work-from-home orders. Note that stay-at-home orders always implied bans of gatherings and venue closures, and bans of large gatherings implied bans of small gatherings. That is, for instance, if a country implemented a ban of small gatherings without yet having implemented a ban of large gatherings, then the implementation date for the ban of small and the ban of large gatherings is the same. In contrast to this, a ban of small gatherings alone does not necessarily imply a venue closure (see, for example, Sweden where recreational facilities, bars, and restaurants were allowed to stay open, despite a ban of small gatherings). Similarly, a work-from-home order does not necessarily imply a venue closure, although this happened to be the case for all countries in our data during the first epidemic wave. Our classification has later been cross-checked against the encoding from the Imperial College COVID-19 Response Team[9] and "Coronavirus Government Response Tracker" from the University of Oxford[22].

3. The date of NPIs has been referred to as the first day a measure went into action. For instance, if a country banned large gatherings with more than 5,000 people on March 1 and banned large gatherings with more than 1,000 people on March 5, then March 1 has been chosen as the date of bans of large gatherings.

4. NPIs for countries that subsequently followed in the data collection have been encoded accordingly.

5. The date of NPIs has been collected for each country or region.

6. A fifth author (BK) checked and verified the collected data. Part of this was also to recruit local residents and/or native speakers from each country in order to check our encoding. The reason for this is that countries have often used different legal terms to refer to the same NPI. Also, checking with local residents has helped to determine, for instance, whether the NPI was actually enforced or just recommended.

8.2 Data Sources

In Table 6, we provide sources of our data on NPIs at a national level. For less centrally managed countries, the aggregated (national level) date of the NPI is shown and the regional sources are displayed in Table 7 (United States), Table 8 (Germany), Table 9 (Spain), Table 10 (Canada), Table 11 (Australia), and Table 12 (Italy). Note that border closures are defined as a national closure of borders and is thus only considered at a national level.

In the following, we want to highlight some encoding decisions that were subject to internal discussions.

- School closure in New South Wales (Australia): Schools have remained technically open but attendance dropped below 5%. Therefore, schools have been encoded as closed.

- Border closure in the US: The US has not stopped flights to all countries but closed their land borders and has suspended travel from a huge number of Asian and European countries. Hence, US borders have been encoded as closed.

- Border closure in Germany: Germany has not closed its borders with Belgium and the Netherlands, therefore not all land borders have been closed and we have decided to encode them as open.

- School closure in Sweden: Only upper secondary schools (16+ y/o) and universities are closed, while other schools are still open. Thus, Swedish schools have been encoded as open.

- Venue closure in Belgium: In Belgium, bars and restaurants were ordered to close on April 14[*], while other non-essential businesses followed only a few days later[†]. In Austria, shops

[*] see https://www.vrt.be/vrtnws/en/2020/03/14/tensions-as-belgium-closes-bars-and-restaurants/
[†] see https://www.reuters.com/article/health-coronavirus-belgium-lockdown-idUSS8N2K307D

were ordered to close on March 15, while bars and restaurants were allowed to stay open until 3pm[‡]. In both cases, the earlier dates (April 14 and March 15) were considered as the dates when venues were closed.

[‡] see https://www.bundeskanzleramt.gv.at/bundeskanzleramt/nachrichten-der-bundesregierung/2020/bundesregierung-praesentiert-aktuelle-beschluesse-zum-coronavirus.html

Country	NPI	Date effective	Source
Australia	Ban of small gatherings		Date derived by cumulative share, see Tab. 11
	Border closure	2020-03-20	https://www.pm.gov.au/media/border-restrictions
	Venue closure		Date derived by cumulative share, see Tab. 11
Austria	Ban of large gatherings	2020-03-11	https://www.bundeskanzleramt.gv.at/bundeskanzleramt/nachrichten-der-bundesregierung/2020/weitere-massnahmen-gegen-ausbreitung-des-coronavirus.html
	Ban of small gatherings	2020-03-16	https://www.reuters.com/article/us-health-coronavirus-austria/austria-imposes-major-restrictions-on-movement-over-coronavirus-idUSKBN2120D8
	School closure	2020-03-16	https://www.reuters.com/article/us-health-coronavirus-austria/austria-closing-schools-over-coronavirus-as-border-checks-take-effect-idUSKBN20Y2YC
	Border closure	2020-03-19	https://www.reuters.com/article/us-health-coronavirus-austria/coronavirus-infections-top-2000-in-austria-more-border-controls-imposed-idUSKBN2160WK
	Venue closure	2020-03-16	https://www.bundeskanzleramt.gv.at/bundeskanzleramt/nachrichten-der-bundesregierung/2020/bundesregierung-praesentiert-aktuelle-beschluesse-zum-coronavirus.html
	Lockdown	2020-03-16	https://www.sozialministerium.at/Informationen-zum-Coronavirus/Coronavirus---Aktuelle-Ma%C3%9Fnahmen.html
Belgium	Ban of large gatherings	2020-03-10	https://www.info-coronavirus.be/en/news/protect-yourself-and-protect-the-others/
	Ban of small gatherings	2020-03-18	https://de.reuters.com/article/health-coronavirus-belgium-lockdown-idUSB5N28S003

	School closure	2020-03-16	https://www.info-coronavirus.be/en/2020/03/12/phase-2-maintained-transition-to-the-federal-phase-and-additional-measures/
	Border closure	2020-03-20	https://www.politico.eu/article/belgium-closes-borders-for-non-essential-travel/
	Venue closure	2020-03-14	https://www.info-coronavirus.be/en/2020/03/12/phase-2-maintained-transition-to-the-federal-phase-and-additional-measures/
	Lockdown	2020-03-18	https://de.reuters.com/article/health-coronavirus-belgium-lockdown-idUSB5N28S003
Canada	Ban of large gatherings		Date derived by cumulative share, see Tab. 10
	Ban of small gatherings		Date derived by cumulative share, see Tab. 10
	School closure		Date derived by cumulative share, see Tab. 10
	Border closure	2020-03-21	https://www.canada.ca/en/public-health/services/diseases/2019-novel-coronavirus-infection/canadas-reponse.html?topic=tilelink
	Venue closure		Date derived by cumulative share, see Tab. 10
Denmark	Ban of large gatherings	2020-03-16	https://www.regeringen.dk/nyheder/pressemoede-11-marts-i-spejlsalen/
	Ban of small gatherings	2020-03-18	https://www.reuters.com/article/us-health-coronavirus-denmark/denmark-bans-crowds-of-over-10-people-to-curb-coronavirus-idUSKBN2143KG
	School closure	2020-03-16	https://www.regeringen.dk/nyheder/pressemoede-11-marts-i-spejlsalen/
	Border closure	2020-03-16	https://www.oresunddirekt.dk/en/news/danish-borders-closed-due-to-coronavirus-covid-19
	Venue closure	2020-03-18	https://www.reuters.com/article/us-health-coronavirus-denmark/denmark-bans-crowds-of-over-10-people-to-curb-coronavirus-idUSKBN2143KG

Finland	Ban of large gatherings	2020-03-16	https://valtioneuvosto.fi/en/article/-/asset_publisher/10616/hallitus-totesi-suomen-olevan-poikkeusoloissa-koronavirustilanteen-vuoksi
	Ban of small gatherings	2020-03-16	https://valtioneuvosto.fi/en/article/-/asset_publisher/10616/hallitus-totesi-suomen-olevan-poikkeusoloissa-koronavirustilanteen-vuoksi
	School closure	2020-03-16	https://valtioneuvosto.fi/en/article/-/asset_publisher/10616/hallitus-totesi-suomen-olevan-poikkeusoloissa-koronavirustilanteen-vuoksi
	Border closure	2020-03-19	https://valtioneuvosto.fi/en/article/-/asset_publisher/1410869/suomen-rajaliikennetta-aletaan-rajoittaa-elakkeella-olevia-rajavartijoita-ja-poliiseja-voidaan-kutsua-toihin
	Venue closure	2020-03-16	https://valtioneuvosto.fi/en/article/-/asset_publisher/10616/hallitus-totesi-suomen-olevan-poikkeusoloissa-koronavirustilanteen-vuoksi
France	Ban of large gatherings	2020-03-13	https://www.bbc.com/news/world-europe-51892477
	Ban of small gatherings	2020-03-13	https://www.bbc.com/news/world-europe-51892477
	School closure	2020-03-16	https://www.bbc.com/news/world-europe-51892477
	Border closure	2020-03-17	https://www.gouvernement.fr/info-coronavirus
	Venue closure	2020-03-14	https://www.gouvernement.fr/partage/11444-declaration-de-m-edouard-philippe-premier-ministre-sur-le-covid-19
	Lockdown	2020-03-17	https://www.gouvernement.fr/info-coronavirus
	Work-from-home order	2020-03-17	https://www.gouvernement.fr/info-coronavirus
Germany	Ban of large gatherings		Date derived by cumulative share, see Tab. 8

	Ban of small gatherings		Date derived by cumulative share, see Tab. 8
	School closure		Date derived by cumulative share, see Tab. 8
	Venue closure		Date derived by cumulative share, see Tab. 8
Greece	Ban of large gatherings	2020-03-13	https://www.reuters.com/article/us-health-coronavirus-greece-measures/greece-to-shut-shops-quarantine-all-arrivals-from-abroad-idUSKBN2131SF
	Ban of small gatherings	2020-03-18	https://www.reuters.com/article/us-health-coronavirus-greece-curfew/greece-imposes-lockdown-after-coronavirus-infections-jump-idUSKBN2190Z1
	School closure	2020-03-11	https://www.reuters.com/article/us-health-coronavirus-greece-education/greece-shuts-schools-universities-to-halt-coronavirus-spread-idUSKBN20X28V
	Venue closure	2020-03-14	https://www.cnn.gr/news/ellada/story/211153/koronoios-poia-katastimata-kleinoyn-poia-menoyn-anoikta-lista
	Lockdown	2020-03-23	https://www.reuters.com/article/us-health-coronavirus-greece-curfew/greece-imposes-lockdown-after-coronavirus-infections-jump-idUSKBN2190Z1
Ireland	Ban of large gatherings	2020-03-12	https://www.gov.ie/en/press-release/96eb4c-statement-from-the-national-public-health-emergency-team/
	Ban of small gatherings	2020-03-28	https://www.gov.ie/en/publication/539d23-stay-at-home-the-latest-public-health-measures-to-prevent-the-spread/
	School closure	2020-03-12	https://www.gov.ie/en/press-release/96eb4c-statement-from-the-national-public-health-emergency-team/
	Venue closure	2020-03-15	https://www.gov.ie/en/press-release/20fc58-all-pubs-advised-to-close-until-march-29/
	Lockdown	2020-03-28	https://www.gov.ie/en/publication/539d23-stay-at-home-the-latest-public-health-measures-to-prevent-the-spread/

	Work-from-home order	2020-03-28	https://www.gov.ie/en/publication/539d23-stay-at-home-the-latest-public-health-measures-to-prevent-the-spread/
Italy	Ban of large gatherings		Date derived by cumulative share, see Tab. 12
	Ban of small gatherings		Date derived by cumulative share, see Tab. 12
	School closure		Date derived by cumulative share, see Tab. 12
	Border closure	2020-03-26	https://www.gazzettaufficiale.it/showNewsDetail?id=2553&backTo=archivio&anno=2020&provenienza=archivio
	Venue closure		Date derived by cumulative share, see Tab. 12
	Lockdown		Date derived by cumulative share, see Tab. 12
	Work-from-home order		Date derived by cumulative share, see Tab. 12
Luxembourg	Ban of large gatherings	2020-03-11	https://gouvernement.lu/fr/actualites/toutes_actualites/communiques/2020/03-mars/11-conseil-gouvernement.html
	Ban of small gatherings	2020-03-17	https://coronavirus.gouvernement.lu/en/communications-officielles.gouvernement%2Ben%2Bactualites%2Btoutes_actualites%2Bcommuniques%2B2020%2B03-mars%2B17-declaration-premier-chd.html
	School closure	2020-03-16	https://coronavirus.gouvernement.lu/en/communications-officielles.gouvernement%2Ben%2Bactualites%2Btoutes_actualites%2Bcommuniques%2B2020%2B03-mars%2B12-cdg-extraordinaire-coronavirus.html
	Venue closure	2020-03-16	https://coronavirus.gouvernement.lu/en/communications-officielles.gouvernement%2Ben%2Bactualites%2Btoutes_actualites%2Bcommuniques%2B2020%2B03-mars%2B15-nouvelles-mesures-coronavirus.html

	Lockdown	2020-03-17	https://coronavirus.gouvernement.lu/en/communications-officielles.gouvernement%2Ben%2Bactualites%2Btoutes_actualites%2Bcommuniques%2B2020%2B03-mars%2B17-declaration-premier-chd.html
	Work-from-home order	2020-03-18	http://www.legilux.lu/eli/etat/leg/rgd/2020/03/18/a165/jo
Netherlands	Ban of large gatherings	2020-03-12	https://www.government.nl/latest/news/2020/03/12/new-measures-to-stop-spread-of-coronavirus-in-the-netherlands
	Ban of small gatherings	2020-03-23	https://www.rijksoverheid.nl/actueel/nieuws/2020/03/24/aanvullende-maatregelen-23-maart
	School closure	2020-03-16	https://www.government.nl/latest/news/2020/03/15/additional-measures-in-schools-the-hospitality-sector-and-sport
	Venue closure	2020-03-16	https://www.government.nl/latest/news/2020/03/15/additional-measures-in-schools-the-hospitality-sector-and-sport
Norway	Ban of large gatherings	2020-03-12	https://www.fhi.no/nettpub/coronavirus/rad-og-informasjon-til-andre-sektorer-og-yrkesgrupper/anbefalinger-ved--store-arrangementer-knyttet-til-koronasmitte-i-norge/
	School closure	2020-03-13	https://www.regjeringen.no/en/aktuelt/coronavirus-measures-to-continue/id2694682/
	Border closure	2020-03-16	https://www.regjeringen.no/no/aktuelt/innforer-strengere-grensekontroll/id2693624/
	Venue closure	2020-03-13	https://www.regjeringen.no/en/aktuelt/coronavirus-measures-to-continue/id2694682/
Portugal	Ban of large gatherings	2020-03-15	https://dre.pt/web/guest/home/-/dre/130277342/details/maximized

	Ban of small gatherings	2020-03-22	https://dre.pt/web/guest/legislacao-consolidada/-/lc/130473378/202004042116/73800717/diploma/indice
	School closure	2020-03-16	https://www.portugal.gov.pt/pt/gc22/comunicacao/comunicado?i=suspensao-de-todas-as-atividades-letivas-e-nao-letivas-com-presenca-de-estudantes-em-todas-as-instituicoes-de-ensino-superior
	Venue closure	2020-03-22	https://dre.pt/web/guest/legislacao-consolidada/-/lc/130473378/202004042116/73800717/diploma/indice
	Lockdown	2020-03-22	https://dre.pt/web/guest/legislacao-consolidada/-/lc/130473378/202004042116/73800717/diploma/indice
Spain	Ban of large gatherings		Date derived by cumulative share, see Tab. 9
	Ban of small gatherings		Date derived by cumulative share, see Tab. 9
	School closure		Date derived by cumulative share, see Tab. 9
	Border closure	2020-03-17	https://english.elpais.com/society/2020-03-16/spain-closes-its-borders-to-contain-coronavirus.html
	Venue closure		Date derived by cumulative share, see Tab. 9
	Lockdown		Date derived by cumulative share, see Tab. 9
	Work-from-home order		Date derived by cumulative share, see Tab. 9
Sweden	Ban of large gatherings	2020-03-11	https://www.government.se/articles/2020/03/ordinance-on-a-prohibition-against-holding-public-gatherings-and-events/
	Ban of small gatherings	2020-03-27	https://www.dailymail.co.uk/news/article-8160653/Sweden-bans-gatherings-50-people-threatens-people-six-month-jail-terms.html
	Ban of large gatherings		Date derived by cumulative share, see Tab. 13
Switzerland			

	Ban of small gatherings		Date derived by cumulative share, see Tab. 13
	School closure		Date derived by cumulative share, see Tab. 13
	Border closure	2020-03-25	https://www.bag.admin.ch/bag/it/home/krankheiten/ausbrueche-epidemien-pandemien/aktuelle-ausbrueche-epidemien/novel-cov/massnahmen-des-bundes.html
	Venue closure		Date derived by cumulative share, see Tab. 13
United Kingdom	Ban of large gatherings	2020-03-16	https://www.gov.uk/guidance/covid-19-guidance-for-mass-gatherings
	Ban of small gatherings	2020-03-23	https://www.gov.uk/government/speeches/pm-address-to-the-nation-on-coronavirus-23-march-2020
	School closure	2020-03-23	https://www.gov.uk/government/speeches/pm-statement-on-coronavirus-22-march-2020
	Venue closure	2020-03-20	https://www.wsj.com/articles/u-k-escalates-measures-to-fight-coronavirus-11584741690
	Lockdown	2020-03-23	https://www.gov.uk/government/speeches/pm-address-to-the-nation-on-coronavirus-23-march-2020
United States of America	Ban of large gatherings		Date derived by cumulative share, see Tab. 7
	Ban of small gatherings		Date derived by cumulative share, see Tab. 7
	School closure		Date derived by cumulative share, see Tab. 7
	Border closure	2020-03-13	https://www.theguardian.com/world/2020/mar/11/coronavirus-outbreak-us-trump-latest#maincontent
	Venue closure		Date derived by cumulative share, see Tab. 7
	Lockdown		Date derived by cumulative share, see Tab. 7

	Work-from-home order	Date derived by cumulative share, see Tab. 7

Table 6. Sources for policies in Switzerland, Austria, Belgium, Denmark, Finland, France, United Kingdom, Greece, Ireland, Luxembourg, Netherlands, Norway, Portugal, Sweden, United States of America, Germany, Italy, Spain, Canada, and Australia

NPI	Date	Cumulative share	Region	Source
Ban of large gatherings	2020-03-11	0.02	Rhode Island	https://www.ri.gov/press/view/37892
Ban of large gatherings	2020-03-11	0.02	Kentucky	https://governor.ky.gov/covid19
Ban of large gatherings	2020-03-11	0.02	District of Columbia	https://wjla.com/news/coronavirus/dc-health-postponing-cancelling-events-1000-people-through-march
Ban of large gatherings	2020-03-12	0.48	Maryland	https://governor.maryland.gov/2020/03/12/governor-hogan-announces-major-actions-to-protect-public-health-limit-spread-of-covid-19-pandemic/
Ban of large gatherings	2020-03-12	0.48	Pennsylvania	https://lancasteronline.com/news/health/gov-tom-wolf-cancel-large-events-with-more-than-250-people/article_1a6f027c-648e-11ea-aa2f-c787d0825889.html
Ban of large gatherings	2020-03-12	0.48	New York	https://www.governor.ny.gov/news/no-2021-continuing-temporary-suspension-and-modification-laws-relating-disaster-emergency
Ban of large gatherings	2020-03-12	0.48	Oregon	https://www.oregon.gov/osp/programs/sfm/Pages/Event_Cancellations.aspx
Ban of large gatherings	2020-03-12	0.48	Indiana	https://www.cdc.gov/coronavirus/2019-ncov/community/large-events/mass-gatherings-ready-for-covid-19.html
Ban of large gatherings	2020-03-12	0.48	Ohio	https://coronavirus.ohio.gov/wps/wcm/connect/gov/b815ab52-a571-4e65-9077-32468779671a/ODH+Order+to+Limit+and+Prohibit+Mass+Gatherings%2C+3.12.20.pdf?MOD=AJPERES&CONVERT_TO=url&CACHEID=ROOTWORKSPACE.Z18_M1HGGIK0N0JO00QO9DDDDM3000-b815ab52-a571-4e65-9077-32468779671a-n5828iN
Ban of large gatherings	2020-03-12	0.48	New Jersey	https://www.nj.gov/governor/news/news/562020/approved/20200312a.shtml

Ban of large gatherings	2020-03-12	0.48	Mississippi	https://www.coronavirus.ms.gov/2019-11/msdh-has-issued-enhanced-protective-recommendations
Ban of large gatherings	2020-03-12	0.48	Florida	https://www.miamiherald.com/news/local/community/miami-dade/article241133076.html
Ban of large gatherings	2020-03-12	0.48	New Mexico	https://www.governor.state.nm.us/2020/03/12/health-secretary-issues-public-health-order-suspending-mass-gatherings-in-new-mexico/
Ban of large gatherings	2020-03-12	0.48	Connecticut	https://portal.ct.gov/-/media/Office-of-the-Governor/Executive-Orders/Lamont-Executive-Orders/Executive-Order-No-7.pdf?la=en
Ban of large gatherings	2020-03-12	0.48	Virginia	https://www.governor.virginia.gov/newsroom/all-releases/2020/march/headline-853537-en.html
Ban of large gatherings	2020-03-12	0.48	California	https://thehill.com/policy/healthcare/487179-california-governor-calls-for-cancellations-of-large-events
Ban of large gatherings	2020-03-12	0.48	West Virginia	https://governor.wv.gov/News/press-releases/2020/Pages/COVID-UPDATE-Gov.-Justice-announces-State-employee-travel-ban,-basketball-tournament-cancellation-among-latest-precautions.aspx
Ban of large gatherings	2020-03-12	0.48	Utah	https://kutv.com/news/local/live-blog-closures-event-cancellations-more-in-utah-due-to-coronavirus
Ban of large gatherings	2020-03-13	0.63	Minnesota	https://apnews.com/24d6d0c93ded9e8a5c2712df0a045854
Ban of large gatherings	2020-03-13	0.63	Washington	https://www.governor.wa.gov/news-media/inslee-announces-statewide-school-closures-expansion-limits-large-gatherings
Ban of large gatherings	2020-03-13	0.63	Louisiana	https://gov.louisiana.gov/index.cfm/communication/viewcampaign/2548?&uid=hgdtfgl%3Dn7&nowrap=1
Ban of large gatherings	2020-03-13	0.63	Arizona	https://www.fox10phoenix.com/news/arizona-governor-says-schools-mass-gatherings-events-of-50-or-more-canceled-amid-covid-19-spread

Ban of large gatherings	2020-03-13	0.63	Massachusetts	https://www.mass.gov/news/governor-baker-issues-order-limiting-large-gatherings-in-the-commonwealth
Ban of large gatherings	2020-03-13	0.63	Michigan	https://www.michigan.gov/whitmer/0,9309,7-387-90499_90705-521595--,00.html
Ban of large gatherings	2020-03-13	0.63	Tennessee	https://www.tn.gov/governor/news/2020/3/13/governor-lee-issues-guidance-for-mass-gatherings--schools-and-state-workforce.html
Ban of large gatherings	2020-03-14	0.66	North Carolina	https://files.nc.gov/governor/documents/files/EO117-COVID-19-Prohibiting-Mass-Gathering-and-K12-School-Closure.pdf
Ban of large gatherings	2020-03-15	0.67	Puerto Rico	https://www.estado.pr.gov/es/ordenes-ejecutivas/
Ban of large gatherings	2020-03-16	1.00	South Carolina	https://eu.usatoday.com/story/news/health/2020/03/16/coronavirus-live-updates-us-death-toll-rises-cases-testing/5053816002/
Ban of large gatherings	2020-03-16	1.00	Oklahoma	https://eu.usatoday.com/story/news/health/2020/03/16/coronavirus-live-updates-us-death-toll-rises-cases-testing/5053816002/
Ban of large gatherings	2020-03-16	1.00	South Dakota	https://eu.usatoday.com/story/news/health/2020/03/16/coronavirus-live-updates-us-death-toll-rises-cases-testing/5053816002/
Ban of large gatherings	2020-03-16	1.00	North Dakota	https://eu.usatoday.com/story/news/health/2020/03/16/coronavirus-live-updates-us-death-toll-rises-cases-testing/5053816002/
Ban of large gatherings	2020-03-16	1.00	Texas	https://eu.usatoday.com/story/news/health/2020/03/16/coronavirus-live-updates-us-death-toll-rises-cases-testing/5053816002/
Ban of large gatherings	2020-03-16	1.00	Vermont	https://eu.usatoday.com/story/news/health/2020/03/16/coronavirus-live-updates-us-death-toll-rises-cases-testing/5053816002/
Ban of large gatherings	2020-03-16	1.00	Alabama	https://eu.usatoday.com/story/news/health/2020/03/16/coronavirus-live-updates-us-death-toll-rises-cases-testing/5053816002/
Ban of large gatherings	2020-03-16	1.00	Missouri	https://governor.mo.gov/press-releases/archive/governor-parsons-statement-regarding-cdc-recommendations-mass-gatherings-and

Ban of large gatherings	2020-03-16	1.00	Nevada	https://eu.usatoday.com/story/news/health/2020/03/16/coronavirus-live-updates-us-death-toll-rises-cases-testing/5053816002/
Ban of large gatherings	2020-03-16	1.00	Alaska	https://eu.usatoday.com/story/news/health/2020/03/16/coronavirus-live-updates-us-death-toll-rises-cases-testing/5053816002/
Ban of large gatherings	2020-03-16	1.00	Arkansas	https://eu.usatoday.com/story/news/health/2020/03/16/coronavirus-live-updates-us-death-toll-rises-cases-testing/5053816002/
Ban of large gatherings	2020-03-16	1.00	Colorado	https://northglenn-thorntonsentinel.com/stories/colorado-events-of-50-people-no-sit-down-restaurants-bars,296332
Ban of large gatherings	2020-03-16	1.00	Delaware	https://eu.usatoday.com/story/news/health/2020/03/16/coronavirus-live-updates-us-death-toll-rises-cases-testing/5053816002/
Ban of large gatherings	2020-03-16	1.00	Georgia	https://eu.usatoday.com/story/news/health/2020/03/16/coronavirus-live-updates-us-death-toll-rises-cases-testing/5053816002/
Ban of large gatherings	2020-03-16	1.00	Hawaii	https://eu.usatoday.com/story/news/health/2020/03/16/coronavirus-live-updates-us-death-toll-rises-cases-testing/5053816002/
Ban of large gatherings	2020-03-16	1.00	New Hampshire	https://www.governor.nh.gov/news-media/press-2020/20200316-covid-10-businesses.htm
Ban of large gatherings	2020-03-16	1.00	Idaho	https://eu.usatoday.com/story/news/health/2020/03/16/coronavirus-live-updates-us-death-toll-rises-cases-testing/5053816002/
Ban of large gatherings	2020-03-16	1.00	Iowa	https://eu.usatoday.com/story/news/health/2020/03/16/coronavirus-live-updates-us-death-toll-rises-cases-testing/5053816002/
Ban of large gatherings	2020-03-16	1.00	Kansas	https://govstatus.egov.com/coronavirus
Ban of large gatherings	2020-03-16	1.00	Maine	https://eu.usatoday.com/story/news/health/2020/03/16/coronavirus-live-updates-us-death-toll-rises-cases-testing/5053816002/
Ban of large gatherings	2020-03-16	1.00	Wisconsin	https://content.govdelivery.com/accounts/WIGOV/bulletins/2817964

Ban of large gatherings	2020-03-16	1.00	Montana	https://eu.usatoday.com/story/news/health/2020/03/16/coronavirus-live-updates-us-death-toll-rises-cases-testing/5053816002/
Ban of large gatherings	2020-03-16	1.00	Nebraska	https://eu.usatoday.com/story/news/health/2020/03/16/coronavirus-live-updates-us-death-toll-rises-cases-testing/5053816002/
Ban of large gatherings	2020-03-16	1.00	Illinois	https://herald-review.com/news/state-and-regional/govt-and-politics/monday-update-pritzker-bans-gatherings-of-50-or-more-12-new-illinois-cases-announced/article_c5fa6e55-70f4-583b-aea9-09ce2315eb16.html
Ban of large gatherings	2020-03-16	1.00	Wyoming	https://eu.usatoday.com/story/news/health/2020/03/16/coronavirus-live-updates-us-death-toll-rises-cases-testing/5053816002/

**NPI**	**Date**	**Cumulative share**	**Region**	**Source**
Ban of small gatherings	2020-03-11	0.01	Kentucky	https://governor.ky.gov/covid19
Ban of small gatherings	2020-03-13	0.04	Arizona	https://www.fox10phoenix.com/news/arizona-governor-says-schools-mass-gatherings-events-of-50-or-more-canceled-amid-covid-19-spread
Ban of small gatherings	2020-03-15	0.05	Puerto Rico	https://www.estado.pr.gov/es/ordenes-ejecutivas/
Ban of small gatherings	2020-03-16	1.00	New Hampshire	https://www.governor.nh.gov/news-media/press-2020/20200316-covid-10-businesses.htm
Ban of small gatherings	2020-03-16	1.00	New Jersey	https://www.governor.nh.gov/news-media/press-2020/20200315-emergency-order-1.html
Ban of small gatherings	2020-03-16	1.00	New Mexico	https://eu.usatoday.com/story/news/health/2020/03/16/coronavirus-live-updates-us-death-toll-rises-cases-testing/5053816002/
Ban of small gatherings	2020-03-16	1.00	New York	https://eu.usatoday.com/story/news/health/2020/03/16/coronavirus-live-updates-us-death-toll-rises-cases-testing/5053816002/

Ban of small gatherings	2020-03-16	1.00	North Carolina	https://eu.usatoday.com/story/news/health/2020/03/16/coronavirus-live-updates-us-death-toll-rises-cases-testing/5053816002/
Ban of small gatherings	2020-03-16	1.00	North Dakota	https://eu.usatoday.com/story/news/health/2020/03/16/coronavirus-live-updates-us-death-toll-rises-cases-testing/5053816002/
Ban of small gatherings	2020-03-16	1.00	Ohio	https://eu.usatoday.com/story/news/health/2020/03/16/coronavirus-live-updates-us-death-toll-rises-cases-testing/5053816002/
Ban of small gatherings	2020-03-16	1.00	Oklahoma	https://eu.usatoday.com/story/news/health/2020/03/16/coronavirus-live-updates-us-death-toll-rises-cases-testing/5053816002/
Ban of small gatherings	2020-03-16	1.00	Oregon	https://www.kptv.com/news/covid--in-oregon-governor-cancels-gatherings-of-more-than/article_8e9aafec-67d2-11ea-af7f-2bad23d52443.html
Ban of small gatherings	2020-03-16	1.00	Pennsylvania	https://www.governor.pa.gov/newsroom/gov-wolf-puts-statewide-covid-19-mitigation-efforts-in-effect-stresses-need-for-every-pennsylvanian-to-take-action-to-stop-the-spread/
Ban of small gatherings	2020-03-16	1.00	Alabama	https://governor.alabama.gov/assets/2020/03/Amended-Statewide-Social-Distancing-SHO-Order-3.27.2020-FINAL.pdf
Ban of small gatherings	2020-03-16	1.00	Rhode Island	https://eu.usatoday.com/story/news/health/2020/03/16/coronavirus-live-updates-us-death-toll-rises-cases-testing/5053816002/
Ban of small gatherings	2020-03-16	1.00	South Carolina	https://eu.usatoday.com/story/news/health/2020/03/16/coronavirus-live-updates-us-death-toll-rises-cases-testing/5053816002/
Ban of small gatherings	2020-03-16	1.00	South Dakota	https://eu.usatoday.com/story/news/health/2020/03/16/coronavirus-live-updates-us-death-toll-rises-cases-testing/5053816002/
Ban of small gatherings	2020-03-16	1.00	Tennessee	https://eu.usatoday.com/story/news/health/2020/03/16/coronavirus-live-updates-us-death-toll-rises-cases-testing/5053816002/
Ban of small gatherings	2020-03-16	1.00	Texas	https://eu.usatoday.com/story/news/health/2020/03/16/coronavirus-live-updates-us-death-toll-rises-cases-testing/5053816002/

Ban of small gatherings	2020-03-16	1.00	Utah	https://eu.usatoday.com/story/news/health/2020/03/16/coronavirus-live-updates-us-death-toll-rises-cases-testing/5053816002/
Ban of small gatherings	2020-03-16	1.00	Vermont	https://eu.usatoday.com/story/news/health/2020/03/16/coronavirus-live-updates-us-death-toll-rises-cases-testing/5053816002/
Ban of small gatherings	2020-03-16	1.00	Virginia	https://eu.usatoday.com/story/news/health/2020/03/16/coronavirus-live-updates-us-death-toll-rises-cases-testing/5053816002/
Ban of small gatherings	2020-03-16	1.00	Washington	https://eu.usatoday.com/story/news/health/2020/03/16/coronavirus-live-updates-us-death-toll-rises-cases-testing/5053816002/
Ban of small gatherings	2020-03-16	1.00	West Virginia	https://eu.usatoday.com/story/news/health/2020/03/16/coronavirus-live-updates-us-death-toll-rises-cases-testing/5053816002/
Ban of small gatherings	2020-03-16	1.00	Nevada	https://eu.usatoday.com/story/news/health/2020/03/16/coronavirus-live-updates-us-death-toll-rises-cases-testing/5053816002/
Ban of small gatherings	2020-03-16	1.00	Nebraska	https://eu.usatoday.com/story/news/health/2020/03/16/coronavirus-live-updates-us-death-toll-rises-cases-testing/5053816002/
Ban of small gatherings	2020-03-16	1.00	Missouri	https://eu.usatoday.com/story/news/health/2020/03/16/coronavirus-live-updates-us-death-toll-rises-cases-testing/5053816002/
Ban of small gatherings	2020-03-16	1.00	Wisconsin	https://content.govdelivery.com/accounts/WIGOV/bulletins/2817964
Ban of small gatherings	2020-03-16	1.00	Alaska	https://eu.usatoday.com/story/news/health/2020/03/16/coronavirus-live-updates-us-death-toll-rises-cases-testing/5053816002/
Ban of small gatherings	2020-03-16	1.00	Arkansas	https://eu.usatoday.com/story/news/health/2020/03/16/coronavirus-live-updates-us-death-toll-rises-cases-testing/5053816002/
Ban of small gatherings	2020-03-16	1.00	California	https://eu.usatoday.com/story/news/health/2020/03/16/coronavirus-live-updates-us-death-toll-rises-cases-testing/5053816002/
Ban of small gatherings	2020-03-16	1.00	Colorado	https://northglenn-thorntonsentinel.com/stories/colorado-events-of-50-people-no-sit-down-restaurants-bars,296332

Ban of small gatherings	2020-03-16	1.00	Connecticut	https://eu.usatoday.com/story/news/health/2020/03/16/coronavirus-live-updates-us-death-toll-rises-cases-testing/5053816002/
Ban of small gatherings	2020-03-16	1.00	Delaware	https://eu.usatoday.com/story/news/health/2020/03/16/coronavirus-live-updates-us-death-toll-rises-cases-testing/5053816002/
Ban of small gatherings	2020-03-16	1.00	District of Columbia	https://dc.gov/release/mayor%E2%80%99s-order-2020-048-prohibition-mass-gatherings-during-public-health-emergency
Ban of small gatherings	2020-03-16	1.00	Florida	https://eu.usatoday.com/story/news/health/2020/03/16/coronavirus-live-updates-us-death-toll-rises-cases-testing/5053816002/
Ban of small gatherings	2020-03-16	1.00	Georgia	https://eu.usatoday.com/story/news/health/2020/03/16/coronavirus-live-updates-us-death-toll-rises-cases-testing/5053816002/
Ban of small gatherings	2020-03-16	1.00	Hawaii	https://eu.usatoday.com/story/news/health/2020/03/16/coronavirus-live-updates-us-death-toll-rises-cases-testing/5053816002/
Ban of small gatherings	2020-03-16	1.00	Montana	https://eu.usatoday.com/story/news/health/2020/03/16/coronavirus-live-updates-us-death-toll-rises-cases-testing/5053816002/
Ban of small gatherings	2020-03-16	1.00	Idaho	https://eu.usatoday.com/story/news/health/2020/03/16/coronavirus-live-updates-us-death-toll-rises-cases-testing/5053816002/
Ban of small gatherings	2020-03-16	1.00	Indiana	https://eu.usatoday.com/story/news/health/2020/03/16/coronavirus-live-updates-us-death-toll-rises-cases-testing/5053816002/
Ban of small gatherings	2020-03-16	1.00	Iowa	https://eu.usatoday.com/story/news/health/2020/03/16/coronavirus-live-updates-us-death-toll-rises-cases-testing/5053816002/
Ban of small gatherings	2020-03-16	1.00	Kansas	https://govstatus.egov.com/coronavirus
Ban of small gatherings	2020-03-16	1.00	Louisiana	https://eu.usatoday.com/story/news/health/2020/03/16/coronavirus-live-updates-us-death-toll-rises-cases-testing/5053816002/
Ban of small gatherings	2020-03-16	1.00	Maine	https://eu.usatoday.com/story/news/health/2020/03/16/coronavirus-live-updates-us-death-toll-rises-cases-testing/5053816002/

Ban of small gatherings	2020-03-16	1.00	Maryland	https://eu.usatoday.com/story/news/health/2020/03/16/coronavirus-live-updates-us-death-toll-rises-cases-testing/5053816002/
Ban of small gatherings	2020-03-16	1.00	Massachusetts	https://eu.usatoday.com/story/news/health/2020/03/16/coronavirus-live-updates-us-death-toll-rises-cases-testing/5053816002/
Ban of small gatherings	2020-03-16	1.00	Michigan	https://www.michigan.gov/whitmer/0,9309,7-387-90499_90705-521890--,00.html
Ban of small gatherings	2020-03-16	1.00	Minnesota	https://eu.usatoday.com/story/news/health/2020/03/16/coronavirus-live-updates-us-death-toll-rises-cases-testing/5053816002/
Ban of small gatherings	2020-03-16	1.00	Mississippi	https://eu.usatoday.com/story/news/health/2020/03/16/coronavirus-live-updates-us-death-toll-rises-cases-testing/5053816002/
Ban of small gatherings	2020-03-16	1.00	Illinois	https://eu.usatoday.com/story/news/health/2020/03/16/coronavirus-live-updates-us-death-toll-rises-cases-testing/5053816002/
Ban of small gatherings	2020-03-16	1.00	Wyoming	https://eu.usatoday.com/story/news/health/2020/03/16/coronavirus-live-updates-us-death-toll-rises-cases-testing/5053816002/

**NPI**	**Date**	**Cumulative share**	**Region**	**Source**
School closure	2020-03-12	0.01	New Mexico	https://www.governor.state.nm.us/2020/03/12/new-mexico-schools-to-temporarily-close/
School closure	2020-03-13	0.05	West Virginia	https://governor.wv.gov/News/press-releases/2020/Pages/COVID19-UPDATE-Gov.-Justice-announces-closure-of-West-Virginia-schools.aspx
School closure	2020-03-13	0.05	Washington	https://www.governor.wa.gov/news-media/inslee-announces-statewide-school-closures-expansion-limits-large-gatherings
School closure	2020-03-13	0.05	Wisconsin	https://content.govdelivery.com/accounts/WIGOV/bulletins/281127d
School closure	2020-03-15	0.06	Montana	http://opi.mt.gov/COVID-19-Information

School closure	2020-03-16	0.50	Nevada	http://www.doe.nv.gov
School closure	2020-03-16	0.50	New Hampshire	https://www.governor.nh.gov/news-media/press-2020/20200315-emergency-order-1.html
School closure	2020-03-16	0.50	New York	https://www.governor.ny.gov/news/governor-cuomo-signs-executive-order-closing-schools-statewide-two-weeks
School closure	2020-03-16	0.50	North Carolina	https://www.dpi.nc.gov/news/press-releases/2020/03/16/state-board-issues-guidance-personnel-facility-matters-covid-19-closure
School closure	2020-03-16	0.50	North Dakota	https://www.nd.gov/dpi/school-closure-frequently-asked-questions
School closure	2020-03-16	0.50	Pennsylvania	https://www.governor.pa.gov/newsroom/governor-wolf-announces-closure-of-pennsylvania-schools/
School closure	2020-03-16	0.50	Puerto Rico	https://twitter.com/wandavazquezg?ref_src=twsrc%5Etfw%7Ctwcamp%5Etweetembed%7Ctwterm%5E1238675549372977153%7Ctwgr%5E&ref_url=https%3A%2F%2Fwww.nbcnews.com%2Fhealth%2Fhealth-news%2Flive-blog%2F2020-03-14-coronavirus-news-n1158821%2Fncrd1158961
School closure	2020-03-16	0.50	South Carolina	https://thehill.com/homenews/state-watch/487708-south-carolina-closes-schools-amid-outbreak
School closure	2020-03-16	0.50	South Dakota	https://www.nd.gov/dpi/executive-orders-education
School closure	2020-03-16	0.50	Tennessee	https://www.tn.gov/governor/news/2020/3/16/governor-lee-issues-statement-regarding-statewide-school-closure.html
School closure	2020-03-16	0.50	Utah	https://www.schools.utah.gov/File/b27ab22a-d14f-4e12-b247-f95becea39d3
School closure	2020-03-16	0.50	Virginia	https://www.governor.virginia.gov/newsroom/all-releases/2020/march/headline-854442-en.html

School closure	2020-03-16	0.50	Michigan	https://www.michigan.gov/whitmer/0,9309,7-387-90499_90705-521890--,00.html
School closure	2020-03-16	0.50	Maryland	https://fox8.com/news/list-states-that-have-closed-all-schools-due-to-coronavirus/
School closure	2020-03-16	0.50	Wyoming	https://www.ktvq.com/wyoming-governor-directs-schools-to-close
School closure	2020-03-16	0.50	Louisiana	https://www.fox61.com/article/news/health/coronavirus/connecticut-coronavirus-updates-march-12/520-ddfcc84c-1521-4354-907b-d53e2642bee9
School closure	2020-03-16	0.50	Alaska	https://www.aljazeera.com/news/2020/03/emergencies-closures-states-handling-coronavirus-200317213356419.html
School closure	2020-03-16	0.50	Arizona	https://www.azed.gov/finance/2020/03/16/school-closures-from-march-16-2020-through-march-27-2020/
School closure	2020-03-16	0.50	Kentucky	https://governor.ky.gov/covid19
School closure	2020-03-16	0.50	Delaware	https://www.abc27.com/news/list-states-that-have-closed-all-schools-due-to-coronavirus/
School closure	2020-03-16	0.50	Illinois	https://www.chicagotribune.com/coronavirus/ct-cb-coronavirus-illinois-schools-closed-cps-parents-need-to-know-20200317-zrcim5cpcfcgnerkboyx7esg5y-story.html
School closure	2020-03-16	0.50	District of Columbia	https://dcps.dc.gov/coronavirus
School closure	2020-03-16	0.50	Hawaii	https://www.aljazeera.com/news/2020/03/emergencies-closures-states-handling-coronavirus-200317213356419.html
School closure	2020-03-16	0.50	Florida	http://www.fldoe.org/newsroom/latest-news/florida-department-of-education-announces-additional-guidance-for-the-2019-20-school-year.stml

School closure	2020-03-16	0.50	Maine	https://www.pressherald.com/2020/03/14/scarborough-closes-schools-through-at-least-march-20/
School closure	2020-03-17	0.74	Massachusetts	https://fox8.com/news/list-states-that-have-closed-all-schools-due-to-coronavirus/
School closure	2020-03-17	0.74	Arkansas	https://www.abc27.com/news/list-states-that-have-closed-all-schools-due-to-coronavirus/
School closure	2020-03-17	0.74	California	https://www.abc27.com/news/list-states-that-have-closed-all-schools-due-to-coronavirus/
School closure	2020-03-17	0.74	Connecticut	https://patch.com/connecticut/guilford/coronavirus-ct-gov-closes-all-schools-state-has-now-26-cases
School closure	2020-03-17	0.74	Rhode Island	https://www.ride.ri.gov/InsideRIDE/AdditionalInformation/Covid19.aspx
School closure	2020-03-17	0.74	Oregon	https://www.oregon.gov/newsroom/Pages/NewsDetail.aspx?newsid=36203
School closure	2020-03-17	0.74	Oklahoma	https://sde.ok.gov/newsblog/2020-03-16/emergency-state-board-meeting-expected-close-schools-until-april-6
School closure	2020-03-17	0.74	Missouri	https://www.abc27.com/news/list-states-that-have-closed-all-schools-due-to-coronavirus/
School closure	2020-03-17	0.74	Ohio	https://coronavirus.ohio.gov/wps/wcm/connect/gov/aeadbec1-574d-4a42-9ca4-4487b7a67a4f/Director%27s+Order+-+K-12+Schools+03.14.20.pdf?MOD=AJPERES&CONVERT_TO=url&CACHEID=ROOTWORKSPACE.Z18_M1HGGIK0N0JO00QO9DDDDM3000-aeadbec1-574d-4a42-9ca4-4487b7a67a4f-n5827Z4
School closure	2020-03-18	0.86	Georgia	https://www.gpb.org/blogs/education-matters/2020/03/16/gov-kemp-orders-all-k-12-georgia-schools-close-until-end-of-march
School closure	2020-03-18	0.86	Idaho	https://www.idahostatesman.com/news/coronavirus/article241291676.html

School closure	2020-03-18	0.86	New Jersey	https://www.nj.gov/governor/news/news/562020/approved/20200316c.shtml
School closure	2020-03-18	0.86	Kansas	https://www.washingtonpost.com/education/2020/03/17/kansas-is-first-state-close-schools-rest-school-year-amid-coronavirus-crisis-california-could-be-next/
School closure	2020-03-18	0.86	Nebraska	https://www.education.ne.gov/publichealth/known-school-closures/
School closure	2020-03-18	0.86	Vermont	https://governor.vermont.gov/press-release/gov-scott-orders-orderly-closure-vermont-prek-12-schools-week
School closure	2020-03-18	0.86	Minnesota	https://mn.gov/governor/assets/EO%2020-02%20Final_tcm1055-423084.pdf
School closure	2020-03-18	0.86	Alabama	https://www.aljazeera.com/news/2020/03/emergencies-closures-states-handling-coronavirus-200317213356419.html
School closure	2020-03-19	0.97	Indiana	https://www.abc27.com/news/list-states-that-have-closed-all-schools-due-to-coronavirus/
School closure	2020-03-19	0.97	Texas	https://www.dallasnews.com/news/public-health/2020/03/19/gov-abbott-announces-temporary-statewide-school-restaurant-gym-closures/
School closure	2020-03-19	0.97	Mississippi	https://www.wlox.com/2020/03/19/gov-tate-reeves-give-update-plans-mississippi-schools/
School closure	2020-03-23	0.99	Colorado	https://www.denverpost.com/2020/03/11/colorado-schools-closed-coronavirus/
**NPI**	**Date**	**Cumulative share**	**Region**	**Source**

Venue closure	2020-03-15	0.09	Pennsylvania	https://www.governor.pa.gov/newsroom/wolf-administration-orders-restaurants-and-bars-to-close-dine-in-service-in-mitigation-counties-to-stop-spread-of-covid-19/
Venue closure	2020-03-15	0.09	Puerto Rico	https://www.estado.pr.gov/es/ordenes-ejecutivas/
Venue closure	2020-03-15	0.09	District of Columbia	https://www.washingtonian.com/2020/03/16/mayor-closes-dc-bars-and-restaurants-for-dine-in-service/
Venue closure	2020-03-15	0.09	Ohio	https://governor.ohio.gov/wps/portal/gov/governor/media/news-and-media/dewine-orders-ohio-bars-restaurants-to-close
Venue closure	2020-03-16	0.48	Louisiana	https://eu.thenewsstar.com/story/news/2020/03/16/louisiana-coronavirus-cases-rise-114-legislature-resume-work/5057909002/
Venue closure	2020-03-16	0.48	New York	https://www.politico.com/states/new-york/albany/story/2020/03/16/new-york-new-jersey-connecticut-closing-bars-restaurants-indefinitely-starting-monday-night-1267159
Venue closure	2020-03-16	0.48	New Jersey	https://www.nj.gov/governor/news/news/562020/approved/20200316a.shtml
Venue closure	2020-03-16	0.48	New Hampshire	https://www.governor.nh.gov/news-media/press-2020/20200316-covid-10-businesses.htm
Venue closure	2020-03-16	0.48	Michigan	https://www.usnews.com/news/best-states/michigan/articles/2020-03-16/michigan-governor-closes-restaurants-to-dine-in-customers
Venue closure	2020-03-16	0.48	Maryland	https://governor.maryland.gov/2020/03/19/governor-hogan-announces-further-actions-to-slow-the-spread-of-covid-19-relaunches-maryland-unites-initiative/
Venue closure	2020-03-16	0.48	Kentucky	https://governor.ky.gov/covid19

Venue closure	2020-03-16	0.48	Indiana	https://www.wndu.com/content/news/Indiana-governor-closes-restaurants-bars-to-dine-in-customers-568830011.html
Venue closure	2020-03-16	0.48	Illinois	https://time.com/5803539/united-states-coronavirus-bars-restaurants/
Venue closure	2020-03-16	0.48	Oregon	https://www.oregon.gov/newsroom/Pages/NewsDetail.aspx?newsid=36192
Venue closure	2020-03-16	0.48	Rhode Island	https://www.ri.gov/press/view/37924
Venue closure	2020-03-16	0.48	Washington	https://www.governor.wa.gov/news-media/inslee-announces-statewide-shutdown-restaurants-bars-and-expanded-social-gathering-limits
Venue closure	2020-03-16	0.48	California	https://www.latimes.com/business/story/2020-03-15/coronavirus-close-los-angeles-restaurants
Venue closure	2020-03-16	0.48	Delaware	https://coronavirus.delaware.gov/wp-content/uploads/sites/177/2020/03/coronavirus_govdec_rest_bars-1.pdf
Venue closure	2020-03-16	0.48	Connecticut	https://www.courant.com/news/connecticut/hc-news-coronavirus-update-0316-20200316-ukhf6fmwh5cvtaob3yiavyzm5q-story.html
Venue closure	2020-03-17	0.69	Wisconsin	https://madison.com/ct/news/local/govt-and-politics/gov-tony-evers-orders-bars-restaurants-to-be-closed-across-wisconsin-for-in-house-dining/article_40abef70-b51e-5756-962f-801f0387eed6.html
Venue closure	2020-03-17	0.69	North Carolina	https://www.newsobserver.com/news/coronavirus/article241245211.html
Venue closure	2020-03-17	0.69	Nevada	https://nvhealthresponse.nv.gov/wp-content/uploads/2020/03/NV-Health-Reponse-COVID19-Risk-Management-Initiative.pdf
Venue closure	2020-03-17	0.69	Colorado	https://www.eater.com/2020/3/15/21180761/coronavirus-restaurants-bars-closed-new-york-la-chicago

Venue closure	2020-03-17	0.69	Vermont	https://governor.vermont.gov/press-release/governor-phil-scott-announces-new-guidance-covid-19-community-mitigation-measures
Venue closure	2020-03-17	0.69	Massachusetts	https://www.mass.gov/news/baker-polito-administration-announces-emergency-actions-to-address-covid-19
Venue closure	2020-03-17	0.69	Minnesota	https://www.eater.com/2020/3/15/21180761/coronavirus-restaurants-bars-closed-new-york-la-chicago
Venue closure	2020-03-17	0.69	South Carolina	https://governor.sc.gov/sites/default/files/Documents/Executive-Orders/
Venue closure	2020-03-17	0.69	Florida	https://www.eater.com/2020/3/15/21180761/coronavirus-restaurants-bars-closed-new-york-la-chicago
Venue closure	2020-03-17	0.69	Iowa	https://governor.iowa.gov/press-release/gov-reynolds-issues-a-state-of-public-health-disaster-emergency
Venue closure	2020-03-18	0.71	Maine	https://www.maine.gov/governor/mills/news/governor-mills-takes-further-steps-respond-covid-19-protect-health-and-safety-maine-people
Venue closure	2020-03-18	0.71	Utah	https://governor.utah.gov/2020/03/18/state-orders-restaurants-bars-to-suspend-dine-in-services-to-slow-spread-of-covid-19/
Venue closure	2020-03-18	0.71	West Virginia	https://governor.wv.gov/News/press-releases/2020/Pages/COVID-19-UPDATE-Executive-Order-limiting-restaurants-and-bars,-closing-casinos-statewide.aspx
Venue closure	2020-03-19	0.83	Nebraska	https://www.3newsnow.com/news/coronavirus/directed-health-measures-released-by-governor-ricketts-office
Venue closure	2020-03-19	0.83	New Mexico	https://www.governor.state.nm.us/2020/03/18/new-mexico-to-order-additional-closures-to-limit-spread-of-covid-19/
Venue closure	2020-03-19	0.83	Arizona	https://azgovernor.gov/governor/news/2020/03/governor-ducey-announces-latest-covid-19-actions

Venue closure	2020-03-19	0.83	Texas	https://www.dallasnews.com/news/public-health/2020/03/19/gov-abbott-announces-temporary-statewide-school-restaurant-gym-closures/
Venue closure	2020-03-19	0.83	Hawaii	https://www.hawaiinewsnow.com/2020/03/19/city-orders-restaurants-bars-night-clubs-close-dine-in-services-days/
Venue closure	2020-03-20	0.85	Wyoming	https://health.wyo.gov/governor-and-state-health-officer-issue-public-spaces-closure-order/
Venue closure	2020-03-20	0.85	North Dakota	https://www.governor.nd.gov/news/burgum-orders-bars-restaurants-closed-site-patrons-provides-additional-guidance-k-12-schools
Venue closure	2020-03-20	0.85	Montana	https://news.mt.gov/governor-bullock-announces-closure-of-dine-in-food-service-and-alcoholic-beverage-businesses-and-other-activities-that-pose-enhanced-risks-to-curtail-spread-of-covid-19
Venue closure	2020-03-20	0.85	Arkansas	https://www.nwaonline.com/news/2020/mar/20/governor-orders-gyms-restaurants-bars-c/
Venue closure	2020-03-20	0.85	South Dakota	https://www.governor.nd.gov/news/burgum-orders-bars-restaurants-closed-site-patrons-provides-additional-guidance-k-12-schools
Venue closure	2020-03-23	0.89	Tennessee	https://www.tn.gov/governor/news/2020/3/22/gov--bill-lee-signs-executive-order-mandating-alternative-business-models-for-restaurants-and-gyms--lifts-alcohol-regulations.html
Venue closure	2020-03-23	0.89	Missouri	https://governor.mo.gov/press-releases/archive/governor-parson-signs-executive-order-20-05-allowing-sale-unprepared-foods
Venue closure	2020-03-24	0.96	Mississippi	https://www.jacksonfreepress.com/documents/2020/mar/24/mississippis-covid-19-response/
Venue closure	2020-03-24	0.96	Virginia	https://www.governor.virginia.gov/newsroom/all-releases/2020/march/headline-855292-en.html
Venue closure	2020-03-24	0.96	Georgia	https://www.usnews.com/news/best-states/georgia/articles/2020-03-23/counties-in-georgia-enact-restrictions-as-virus-spreads

Venue closure	2020-03-24	0.96	Alaska	https://gov.alaska.gov/wp-content/uploads/sites/2/03232020-SOA-COVID-19-Health-Mandate-009.pdf
Venue closure	2020-03-25	0.98	Idaho	https://coronavirus.idaho.gov/essential-services/
Venue closure	2020-03-25	0.98	Oklahoma	https://kfor.com/news/local/gov-stitt-orders-all-non-essential-businesses-to-close-in-counties-affected-by-covid-19/
Venue closure	2020-03-27	0.99	Alabama	https://governor.alabama.gov/assets/2020/03/Amended-Statewide-Social-Distancing-SHO-Order-3.27.2020-FINAL.pdf
Venue closure	2020-03-30	1.00	Kansas	https://governor.kansas.gov/governor-kelly-issues-temporary-statewide-stay-home-order-in-ongoing-effort-to-combat-covid-19/

**NPI**	**Date**	**Cumulative share**	**Region**	**Source**
Lockdown	2020-03-15	0.01	Puerto Rico	https://www.estado.pr.gov/es/ordenes-ejecutivas/
Lockdown	2020-03-19	0.13	California	https://www.gov.ca.gov/2020/03/19/governor-gavin-newsom-issues-stay-at-home-order/
Lockdown	2020-03-21	0.19	New Jersey	https://www.nj.gov/governor/news/news/562020/approved/20200320j.shtml
Lockdown	2020-03-21	0.19	Illinois	https://www2.illinois.gov/Pages/news-item.aspx?ReleaseID=21288
Lockdown	2020-03-22	0.25	New York	https://patch.com/new-york/new-york-city/new-yorks-stay-home-order-goes-effect
Lockdown	2020-03-23	0.35	Oregon	https://www.oregon.gov/newsroom/Pages/NewsDetail.aspx?newsid=36240
Lockdown	2020-03-23	0.35	Ohio	https://coronavirus.ohio.gov/static/DirectorsOrderStayAtHome.pdf
Lockdown	2020-03-23	0.35	Connecticut	https://portal.ct.gov/Office-of-the-Governor/News/Press-Releases/2020/03-2020/Governor-Lamont-Releases-Guidance-to-Businesses-on-Order-Asking-Connecticut-to-Stay-Safe-Stay-Home
Lockdown	2020-03-23	0.35	New Mexico	https://www.governor.state.nm.us/2020/03/23/state-enacts-further-restrictions-to-stop-spread-including-stay-at-home-instruction/

Lockdown	2020-03-23	0.35	Washington	https://www.governor.wa.gov/news-media/inslee-announces-stay-home-stay-healthy%C2%A0order
Lockdown	2020-03-23	0.35	Louisiana	https://gov.louisiana.gov/assets/Proclamations/2020/JBE-33-2020.pdf
Lockdown	2020-03-24	0.42	Michigan	https://www.michigan.gov/coronavirus/0,9753,7-406-98178_98455-521682--,00.html
Lockdown	2020-03-24	0.42	West Virginia	https://governor.wv.gov/News/press-releases/2020/Pages/COVID-19-UPDATE-Gov.-Justice-issues-Stay-at-Home-order-for-all-West-Virginians.aspx
Lockdown	2020-03-24	0.42	Massachusetts	https://www.mass.gov/news/dph-public-health-advisory-stay-at-home-advisory
Lockdown	2020-03-24	0.42	Vermont	https://governor.vermont.gov/sites/scott/files/documents/ADDENDUM%206%20TO%20EXECUTIVE%20ORDER%2001-20.pdf
Lockdown	2020-03-24	0.42	Delaware	https://governor.delaware.gov/wp-content/uploads/sites/24/2020/03/Fifth-Modification-to-State-of-Emergency-03222020.pdf
Lockdown	2020-03-25	0.46	Indiana	https://www.in.gov/gov/3232.htm
Lockdown	2020-03-25	0.46	Hawaii	https://governor.hawaii.gov/newsroom/latest-news/office-of-the-governor-news-release-governor-ige-issues-statewide-order-to-stay-at-home-work-from-home-to-fight-covid-19/
Lockdown	2020-03-25	0.46	Wisconsin	https://content.govdelivery.com/accounts/WIGOV/bulletins/282deef
Lockdown	2020-03-25	0.46	Idaho	https://www.idahostatesman.com/news/coronavirus/article241479406.html
Lockdown	2020-03-26	0.48	New Hampshire	https://www.governor.nh.gov/news-media/emergency-orders/documents/emergency-order-17-1.pdf
Lockdown	2020-03-26	0.48	Colorado	https://bloximages.newyork1.vip.townnews.com/coloradopolitics.com/content/tncms/assets/v3/editorial/1/5c/15c0d646-6efd-11ea-9f44-936924cd21a7/5e7bfea00dc56.pdf.pdf
Lockdown	2020-03-27	0.51	Utah	https://coronavirus.utah.gov/full-text-governors-stay-home-stay-safe-directive/

Lockdown	2020-03-27	0.51	Minnesota	https://mn.gov/governor/assets/3a.%20EO%2020-20%20FINAL%20SIGNED%20Filed_tcm1055-425020.pdf
Lockdown	2020-03-28	0.52	Rhode Island	http://www.governor.ri.gov/documents/orders/Executive-Order-20-13.pdf
Lockdown	2020-03-28	0.52	Montana	https://news.mt.gov/governor-bullock-issues-stay-at-home-directive-to-slow-the-spread-of-covid-19
Lockdown	2020-03-28	0.52	Alaska	https://gov.alaska.gov/home/covid19-healthmandates/
Lockdown	2020-03-30	0.61	Virginia	https://www.governor.virginia.gov/newsroom/all-releases/2020/march/headline-855702-en.html
Lockdown	2020-03-30	0.61	North Carolina	https://files.nc.gov/governor/documents/files/EO121-Stay-at-Home-Order-3.pdf
Lockdown	2020-03-30	0.61	Kansas	https://governor.kansas.gov/wp-content/uploads/2020/03/EO20-16.pdf
Lockdown	2020-03-30	0.61	Maryland	https://www.youtube.com/watch?v=8TPx6pxBCyM
Lockdown	2020-03-30	0.61	District of Columbia	https://coronavirus.dc.gov/release/mayor-bowser-issues-stay-home-order
Lockdown	2020-03-31	0.63	Arizona	https://azgovernor.gov/governor/news/2020/03/stay-home-stay-healthy-stay-connected
Lockdown	2020-04-01	0.76	Florida	https://eu.floridatoday.com/story/news/2020/04/01/coronavirus-florida-stay-home-order-what-means-explanation-what-essential-non-essential-desantis/5104936002/
Lockdown	2020-04-01	0.76	Pennsylvania	https://www.nytimes.com/interactive/2020/us/coronavirus-stay-at-home-order.html
Lockdown	2020-04-01	0.76	Nevada	https://www.fox5vegas.com/coronavirus/nevada-gov-sisolak-issues-stay-at-home-directive-through-april-30/article_6b11e83c-7430-11ea-abe6-0f026faccbed.html
Lockdown	2020-04-01	0.76	Tennessee	https://publications.tnsosfiles.com/pub/execorders/exec-orders-lee22.pdf
Lockdown	2020-04-02	0.89	Georgia	https://gov.georgia.gov/document/2020-executive-order/04022001/download

Lockdown	2020-04-02	0.89	Texas	https://www.nbcnews.com/health/health-news/here-are-stay-home-orders-across-country-n1168736
Lockdown	2020-04-02	0.89	Maine	https://www.maine.gov/governor/mills/news/governor-mills-issues-stay-healthy-home-mandate-2020-03-31
Lockdown	2020-04-03	0.89	Mississippi	https://thehill.com/homenews/state-watch/490674-mississippi-governor-issues-stay-at-home-order
Lockdown	2020-04-04	0.91	Alabama	https://governor.alabama.gov/newsroom/2020/04/governor-ivey-issues-stay-at-home-order/
Lockdown	2020-04-06	0.93	Missouri	https://governor.mo.gov/priorities/stay-home-order
Lockdown	2020-04-07	0.94	South Carolina	http://abcnews4.com/news/local/gov-mcmaster-orders-stay-at-home-order-for-south-carolina

**NPI**	**Date**	**Cumulative share**	**Region**	**Source**
Work-from-home order	2020-03-15	0.01	Puerto Rico	https://www.estado.pr.gov/es/ordenes-ejecutivas/
Work-from-home order	2020-03-19	0.17	California	https://www.gov.ca.gov/2020/03/19/governor-gavin-newsom-issues-stay-at-home-order/
Work-from-home order	2020-03-19	0.17	Pennsylvania	https://www.governor.pa.gov/newsroom/all-non-life-sustaining-businesses-in-pennsylvania-to-close-physical-locations-as-of-8-pm-today-to-slow-spread-of-covid-19/
Work-from-home order	2020-03-21	0.23	Illinois	https://www.nytimes.com/2020/03/21/world/coronavirus-news.html
Work-from-home order	2020-03-21	0.23	New Jersey	https://www.nj.gov/governor/news/news/562020/approved/20200320j.shtml
Work-from-home order	2020-03-22	0.29	New York	https://www.governor.ny.gov/news/no-2028-continuing-temporary-suspension-and-modification-laws-relating-disaster-emergency

Work-from-home order	2020-03-23	0.42	New Mexico	https://www.governor.state.nm.us/2020/03/23/state-enacts-further-restrictions-to-stop-spread-including-stay-at-home-instruction/
Work-from-home order	2020-03-23	0.42	Washington	https://www.governor.wa.gov/news-media/inslee-announces-stay-home-stay-healthy%C2%A0order
Work-from-home order	2020-03-23	0.42	Oregon	https://govstatus.egov.com/or-covid-19
Work-from-home order	2020-03-23	0.42	Connecticut	https://portal.ct.gov/Office-of-the-Governor/News/Press-Releases/2020/03-2020/Governor-Lamont-Signs-Executive-Order-Asking-Connecticut-Businesses-and-Residents-Stay-Safe
Work-from-home order	2020-03-23	0.42	Massachusetts	https://www.mass.gov/news/governor-charlie-baker-orders-all-non-essential-businesses-to-cease-in-person-operation
Work-from-home order	2020-03-23	0.42	Ohio	https://coronavirus.ohio.gov/static/DirectorsOrderStayAtHome.pdf
Work-from-home order	2020-03-23	0.42	Maryland	https://governor.maryland.gov/2020/03/23/governor-hogan-announces-closure-of-all-non-essential-businesses-175-million-relief-package-for-workers-and-small-businesses-affected-by-covid-19/
Work-from-home order	2020-03-24	0.46	Michigan	https://www.michigan.gov/coronavirus/0,9753,7-406-98178_98455-521682--,00.html
Work-from-home order	2020-03-24	0.46	West Virginia	https://governor.wv.gov/News/press-releases/2020/Pages/COVID-19-UPDATE-Gov.-Justice-issues-Stay-at-Home-order-for-all-West-Virginians.aspx
Work-from-home order	2020-03-24	0.46	Delaware	https://governor.delaware.gov/wp-content/uploads/sites/24/2020/03/Fourth-Modification-to-State-of-Emergency-03222020.pdf
Work-from-home order	2020-03-24	0.46	Vermont	https://governor.vermont.gov/sites/scott/files/documents/ADDENDUM%206%20TO%20EXECUTIVE%20ORDER%2001-20.pdf
Work-from-home order	2020-03-25	0.52	Wisconsin	https://content.govdelivery.com/accounts/WIGOV/bulletins/282deef

Work-from-home order	2020-03-25	0.52	Oklahoma	https://www.sos.ok.gov/documents/executive/1919.pdf
Work-from-home order	2020-03-25	0.52	Indiana	https://www.in.gov/gov/3232.htm
Work-from-home order	2020-03-25	0.52	Idaho	https://www.idahostatesman.com/news/coronavirus/article241479406.html
Work-from-home order	2020-03-25	0.52	Hawaii	https://governor.hawaii.gov/newsroom/latest-news/office-of-the-governor-news-release-governor-ige-issues-statewide-order-to-stay-at-home-work-from-home-to-fight-covid-19/
Work-from-home order	2020-03-26	0.55	Colorado	https://bloximages.newyork1.vip.townnews.com/coloradopolitics.com/content/tncms/assets/v3/editorial/1/5c/15c0d646-6efd-11ea-9f44-936924cd21a7/5e7bfea00dc56.pdf.pdf
Work-from-home order	2020-03-26	0.55	New Hampshire	https://www.governor.nh.gov/news-media/emergency-orders/documents/emergency-order-17-1.pdf
Work-from-home order	2020-03-26	0.55	Kentucky	https://governor.ky.gov/attachments/20200325_Executive-Order_2020-257_Healthy-at-Home.pdf
Work-from-home order	2020-03-27	0.58	Alabama	https://governor.alabama.gov/newsroom/2020/04/governor-ivey-issues-stay-at-home-order/
Work-from-home order	2020-03-27	0.58	Minnesota	https://mn.gov/governor/assets/3a.%20EO%2020-20%20FINAL%20SIGNED%20Filed_tcm1055-425020.pdf
Work-from-home order	2020-03-28	0.59	Alaska	https://gov.alaska.gov/home/covid19-healthmandates/
Work-from-home order	2020-03-28	0.59	Montana	https://news.mt.gov/governor-bullock-issues-stay-at-home-directive-to-slow-the-spread-of-covid-19
Work-from-home order	2020-03-30	0.64	Kansas	https://governor.kansas.gov/governor-kelly-issues-temporary-statewide-stay-home-order-in-ongoing-effort-to-combat-covid-19/

Work-from-home order	2020-03-30	0.64	Rhode Island	http://www.governor.ri.gov/documents/orders/Executive-Order-20-13.pdf
Work-from-home order	2020-03-30	0.64	District of Columbia	https://coronavirus.dc.gov/release/mayor-bowser-issues-stay-home-order
Work-from-home order	2020-03-30	0.64	North Carolina	https://files.nc.gov/governor/documents/files/EO121-Stay-at-Home-Order-3.pdf
Work-from-home order	2020-04-01	0.66	Tennessee	https://publications.tnsosfiles.com/pub/execorders/exec-orders-lee22.pdf
Work-from-home order	2020-04-02	0.74	Texas	https://www.nbcnews.com/health/health-news/here-are-stay-home-orders-across-country-n1168736
Work-from-home order	2020-04-07	0.76	South Carolina	http://abcnews4.com/news/local/gov-mcmaster-orders-stay-at-home-order-for-south-carolina

Table 7. Sources for policies implemented across different US States

NPI	Date	Cumulative share	Region	Source
Ban of large gatherings	2020-03-09	0.15	Bavaria	https://www.br.de/nachrichten/bayern/coronavirus-bayern-will-grossveranstaltungen-verbieten,RslNyZO
Ban of large gatherings	2020-03-10	0.37	North Rhine-Westphalia	https://www1.wdr.de/nachrichten/themen/coronavirus/veranstaltungen-corona-virus-absage-nrw-100.html
Ban of large gatherings	2020-03-11	0.70	Baden-Wuerttemberg	https://bnn.de/lokales/karlsruhe/baden-wuerttemberg-will-grosse-veranstaltungen-wegen-des-coronavirus-untersagen-lassen
Ban of large gatherings	2020-03-11	0.70	Berlin	https://www.t-online.de/nachrichten/panorama/id_87498882/coronavirus-in-diesen-bundeslaendern-sind-grossveranstaltungen-verboten.html
Ban of large gatherings	2020-03-11	0.70	Hamburg	https://www.t-online.de/nachrichten/panorama/id_87498882/coronavirus-in-diesen-bundeslaendern-sind-grossveranstaltungen-verboten.html
Ban of large gatherings	2020-03-11	0.70	Lower Saxony	https://www.t-online.de/nachrichten/panorama/id_87498882/coronavirus-in-diesen-bundeslaendern-sind-grossveranstaltungen-verboten.html
Ban of large gatherings	2020-03-11	0.70	Schleswig-Holstein	https://www.ndr.de/nachrichten/schleswig-holstein/Details-zur-Absage-von-Grossveranstaltungen-,pk214.html
Ban of large gatherings	2020-03-12	0.73	Bremen	https://www.t-online.de/nachrichten/panorama/id_87498882/coronavirus-in-diesen-bundeslaendern-sind-grossveranstaltungen-verboten.html
Ban of large gatherings	2020-03-12	0.73	Thuringia	https://www.mdr.de/thueringen/coronavirus-veranstaltungen-massnahmen-teilnehmer-100.html
Ban of large gatherings	2020-03-13	1.00	Brandenburg	https://twitter.com/StM_Klose/status/1238028608469336070
Ban of large gatherings	2020-03-13	1.00	Hesse	https://twitter.com/StM_Klose/status/1238028608469336070

Ban of large gatherings	2020-03-13	1.00	Mecklenburg-Western Pomerania	https://twitter.com/StM_Klose/status/1238028608469336070
Ban of large gatherings	2020-03-13	1.00	Rhineland-Palatinate	https://twitter.com/StM_Klose/status/1238028608469336070
Ban of large gatherings	2020-03-13	1.00	Saarland	https://twitter.com/StM_Klose/status/1238028608469336070
Ban of large gatherings	2020-03-13	1.00	Saxony	https://twitter.com/StM_Klose/status/1238028608469336070
Ban of large gatherings	2020-03-13	1.00	Saxony-Anhalt	https://twitter.com/StM_Klose/status/1238028608469336070

**NPI**	**Date**	**Cumulative share**	**Region**	**Source**
Ban of small gatherings	2020-03-14	0.04	Berlin	https://www.reuters.com/article/us-health-coronavirus-germany/berlin-joins-cologne-in-closing-bars-clubs-as-germany-toughens-coronavirus-response-idUSKBN2110LS
Ban of small gatherings	2020-03-17	0.20	Bavaria	https://www.reuters.com/article/us-health-coronavirus-germany-economy/merkel-says-lets-get-through-this-as-shops-bars-and-churches-shut-idUSKBN213178
Ban of small gatherings	2020-03-23	1.00	Baden-Wuerttemberg	https://www.bundesregierung.de/breg-de/themen/coronavirus/faqs-neue-leitlinien-1733416
Ban of small gatherings	2020-03-23	1.00	Brandenburg	https://www.bundesregierung.de/breg-de/themen/coronavirus/faqs-neue-leitlinien-1733416
Ban of small gatherings	2020-03-23	1.00	Bremen	https://www.bundesregierung.de/breg-de/themen/coronavirus/faqs-neue-leitlinien-1733416
Ban of small gatherings	2020-03-23	1.00	Hamburg	https://www.bundesregierung.de/breg-de/themen/coronavirus/faqs-neue-leitlinien-1733416

Ban of small gatherings	2020-03-23	1.00	Hesse	https://www.bundesregierung.de/breg-de/themen/coronavirus/faqs-neue-leitlinien-1733416
Ban of small gatherings	2020-03-23	1.00	Lower Saxony	https://www.bundesregierung.de/breg-de/themen/coronavirus/faqs-neue-leitlinien-1733416
Ban of small gatherings	2020-03-23	1.00	Mecklenburg-Western Pomerania	https://www.bundesregierung.de/breg-de/themen/coronavirus/faqs-neue-leitlinien-1733416
Ban of small gatherings	2020-03-23	1.00	North Rhine-Westphalia	https://www.bundesregierung.de/breg-de/themen/coronavirus/faqs-neue-leitlinien-1733416
Ban of small gatherings	2020-03-23	1.00	Rhineland-Palatinate	https://www.bundesregierung.de/breg-de/themen/coronavirus/faqs-neue-leitlinien-1733416
Ban of small gatherings	2020-03-23	1.00	Saarland	https://www.bundesregierung.de/breg-de/themen/coronavirus/faqs-neue-leitlinien-1733416
Ban of small gatherings	2020-03-23	1.00	Saxony	https://www.bundesregierung.de/breg-de/themen/coronavirus/faqs-neue-leitlinien-1733416
Ban of small gatherings	2020-03-23	1.00	Saxony-Anhalt	https://www.bundesregierung.de/breg-de/themen/coronavirus/faqs-neue-leitlinien-1733416
Ban of small gatherings	2020-03-23	1.00	Schleswig-Holstein	https://www.bundesregierung.de/breg-de/themen/coronavirus/faqs-neue-leitlinien-1733416
Ban of small gatherings	2020-03-23	1.00	Thuringia	https://www.bundesregierung.de/breg-de/themen/coronavirus/faqs-neue-leitlinien-1733416

**NPI**	**Date**	**Cumulative share**	**Region**	**Source**
School closure	2020-03-16	0.81	Bavaria	https://www.spiegel.de/international/germany/the-shutdown-begins-across-germany-a-3c541d1d-1d42-4672-9fdc-af6c3247df76
School closure	2020-03-16	0.81	Berlin	https://www.spiegel.de/international/germany/the-shutdown-begins-across-germany-a-3c541d1d-1d42-4672-9fdc-af6c3247df76

School closure	2020-03-16	0.81	Bremen	https://www.spiegel.de/international/germany/the-shutdown-begins-across-germany-a-3c541d1d-1d42-4672-9fdc-af6c3247df76
School closure	2020-03-16	0.81	Hamburg	https://www.spiegel.de/international/germany/the-shutdown-begins-across-germany-a-3c541d1d-1d42-4672-9fdc-af6c3247df76
School closure	2020-03-16	0.81	Hesse	https://www.spiegel.de/international/germany/the-shutdown-begins-across-germany-a-3c541d1d-1d42-4672-9fdc-af6c3247df76
School closure	2020-03-16	0.81	Lower Saxony	https://www.spiegel.de/international/germany/the-shutdown-begins-across-germany-a-3c541d1d-1d42-4672-9fdc-af6c3247df76
School closure	2020-03-16	0.81	Mecklenburg-Western Pomerania	https://www.spiegel.de/international/germany/the-shutdown-begins-across-germany-a-3c541d1d-1d42-4672-9fdc-af6c3247df76
School closure	2020-03-16	0.81	North Rhine-Westphalia	https://www.spiegel.de/international/germany/the-shutdown-begins-across-germany-a-3c541d1d-1d42-4672-9fdc-af6c3247df76
School closure	2020-03-16	0.81	Rhineland-Palatinate	https://www.spiegel.de/international/germany/the-shutdown-begins-across-germany-a-3c541d1d-1d42-4672-9fdc-af6c3247df76
School closure	2020-03-16	0.81	Saarland	https://www.spiegel.de/international/germany/the-shutdown-begins-across-germany-a-3c541d1d-1d42-4672-9fdc-af6c3247df76
School closure	2020-03-16	0.81	Saxony	https://www.spiegel.de/international/germany/germany-states-move-to-close-educational-and-daycare-facilities-a-e9c13296-002b-484b-88bc-e14ea295ff10
School closure	2020-03-16	0.81	Saxony-Anhalt	https://www.spiegel.de/international/germany/the-shutdown-begins-across-germany-a-3c541d1d-1d42-4672-9fdc-af6c3247df76
School closure	2020-03-16	0.81	Schleswig-Holstein	https://www.spiegel.de/international/germany/the-shutdown-begins-across-germany-a-3c541d1d-1d42-4672-9fdc-af6c3247df76
School closure	2020-03-17	0.97	Baden-Wuerttemberg	https://www.spiegel.de/international/germany/the-shutdown-begins-across-germany-a-3c541d1d-1d42-4672-9fdc-af6c3247df76

School closure	2020-03-17	0.97	Thuringia	https://www.spiegel.de/international/germany/the-shutdown-begins-across-germany-a-3c541d1d-1d42-4672-9fdc-af6c3247df76
School closure	2020-03-18	1.00	Brandenburg	https://www.spiegel.de/international/germany/the-shutdown-begins-across-germany-a-3c541d1d-1d42-4672-9fdc-af6c3247df76

NPI	Date	Cumulative share	Region	Source
Venue closure	2020-03-14	0.05	Berlin	https://www.reuters.com/article/us-health-coronavirus-germany/berlin-joins-cologne-in-closing-bars-clubs-as-germany-toughens-coronavirus-response-idUSKBN2110LS
Venue closure	2020-03-14	0.05	Saarland	https://www.reuters.com/article/us-health-coronavirus-germany/berlin-joins-cologne-in-closing-bars-clubs-as-germany-toughens-coronavirus-response-idUSKBN2110LS
Venue closure	2020-03-16	1.00	Baden-Wuerttemberg	https://www.bundesregierung.de/breg-de/themen/coronavirus/leitlinien-bund-laender-1731000
Venue closure	2020-03-16	1.00	Bavaria	https://www.bundesregierung.de/breg-de/themen/coronavirus/leitlinien-bund-laender-1731000
Venue closure	2020-03-16	1.00	Brandenburg	https://www.bundesregierung.de/breg-de/themen/coronavirus/leitlinien-bund-laender-1731000
Venue closure	2020-03-16	1.00	Bremen	https://www.bundesregierung.de/breg-de/themen/coronavirus/leitlinien-bund-laender-1731000
Venue closure	2020-03-16	1.00	Hamburg	https://www.bundesregierung.de/breg-de/themen/coronavirus/leitlinien-bund-laender-1731000
Venue closure	2020-03-16	1.00	Hesse	https://www.bundesregierung.de/breg-de/themen/coronavirus/leitlinien-bund-laender-1731000
Venue closure	2020-03-16	1.00	Lower Saxony	https://www.bundesregierung.de/breg-de/themen/coronavirus/leitlinien-bund-laender-1731000

Venue closure	2020-03-16	1.00	Mecklenburg-Western Pomerania	https://www.bundesregierung.de/breg-de/themen/coronavirus/leitlinien-bund-laender-1731000
Venue closure	2020-03-16	1.00	North Rhine-Westphalia	https://www.bundesregierung.de/breg-de/themen/coronavirus/leitlinien-bund-laender-1731000
Venue closure	2020-03-16	1.00	Rhineland-Palatinate	https://www.bundesregierung.de/breg-de/themen/coronavirus/leitlinien-bund-laender-1731000
Venue closure	2020-03-16	1.00	Saxony	https://www.bundesregierung.de/breg-de/themen/coronavirus/leitlinien-bund-laender-1731000
Venue closure	2020-03-16	1.00	Saxony-Anhalt	https://www.bundesregierung.de/breg-de/themen/coronavirus/leitlinien-bund-laender-1731000
Venue closure	2020-03-16	1.00	Schleswig-Holstein	https://www.bundesregierung.de/breg-de/themen/coronavirus/leitlinien-bund-laender-1731000
Venue closure	2020-03-16	1.00	Thuringia	https://www.bundesregierung.de/breg-de/themen/coronavirus/leitlinien-bund-laender-1731000

**NPI**	**Date**	**Cumulative share**	**Region**	**Source**
Lockdown	2020-03-21	0.15	Bavaria	https://www.corona-katastrophenschutz.bayern.de/

**NPI**	**Date**	**Cumulative share**	**Region**	**Source**
Work-from-home order	*NPI implemented in no region*			

Table 8. Sources for policies implemented across different German regions

NPI	Date	Cumulative share	Region	Source
Ban of large gatherings	2020-03-10	0.15	La Rioja	https://www.eldiario.es/sociedad/Sanidad-consejo-ministros-medidas_0_1004400105.html
Ban of large gatherings	2020-03-10	0.15	Community of Madrid	https://www.eldiario.es/sociedad/Sanidad-consejo-ministros-medidas_0_1004400105.html
Ban of large gatherings	2020-03-12	0.31	Catalonia	https://www.catalannews.com/society-science/item/catalonia-bans-events-of-over-1000-people-in-efforts-to-control-coronavirus
Ban of large gatherings	2020-03-13	1.00	Andalusia	https://elpais.com/sociedad/2020-03-12/el-gobierno-extiende-a-toda-espana-la-recomendacion-de-cancelar-clases-y-celebrar-eventos-masivos-en-espacios-cerrados.html
Ban of large gatherings	2020-03-13	1.00	Navarre	https://elpais.com/sociedad/2020-03-12/el-gobierno-extiende-a-toda-espana-la-recomendacion-de-cancelar-clases-y-celebrar-eventos-masivos-en-espacios-cerrados.html
Ban of large gatherings	2020-03-13	1.00	Galicia	https://elpais.com/sociedad/2020-03-12/el-gobierno-extiende-a-toda-espana-la-recomendacion-de-cancelar-clases-y-celebrar-eventos-masivos-en-espacios-cerrados.html
Ban of large gatherings	2020-03-13	1.00	Extremadura	https://elpais.com/sociedad/2020-03-12/el-gobierno-extiende-a-toda-espana-la-recomendacion-de-cancelar-clases-y-celebrar-eventos-masivos-en-espacios-cerrados.html
Ban of large gatherings	2020-03-13	1.00	Region of Murcia	https://elpais.com/sociedad/2020-03-12/el-gobierno-extiende-a-toda-espana-la-recomendacion-de-cancelar-clases-y-celebrar-eventos-masivos-en-espacios-cerrados.html

Ban of large gatherings	2020-03-13	1.00	Castille-La Mancha	https://elpais.com/sociedad/2020-03-12/el-gobierno-extiende-a-toda-espana-la-recomendacion-de-cancelar-clases-y-celebrar-eventos-masivos-en-espacios-cerrados.html
Ban of large gatherings	2020-03-13	1.00	Cantabria	https://elpais.com/sociedad/2020-03-12/el-gobierno-extiende-a-toda-espana-la-recomendacion-de-cancelar-clases-y-celebrar-eventos-masivos-en-espacios-cerrados.html
Ban of large gatherings	2020-03-13	1.00	Canary Islands	https://elpais.com/sociedad/2020-03-12/el-gobierno-extiende-a-toda-espana-la-recomendacion-de-cancelar-clases-y-celebrar-eventos-masivos-en-espacios-cerrados.html
Ban of large gatherings	2020-03-13	1.00	Basque Country	https://elpais.com/sociedad/2020-03-12/el-gobierno-extiende-a-toda-espana-la-recomendacion-de-cancelar-clases-y-celebrar-eventos-masivos-en-espacios-cerrados.html
Ban of large gatherings	2020-03-13	1.00	Balearic Islands	https://elpais.com/sociedad/2020-03-12/el-gobierno-extiende-a-toda-espana-la-recomendacion-de-cancelar-clases-y-celebrar-eventos-masivos-en-espacios-cerrados.html
Ban of large gatherings	2020-03-13	1.00	Asturias	https://elpais.com/sociedad/2020-03-12/el-gobierno-extiende-a-toda-espana-la-recomendacion-de-cancelar-clases-y-celebrar-eventos-masivos-en-espacios-cerrados.html
Ban of large gatherings	2020-03-13	1.00	Aragon	https://elpais.com/sociedad/2020-03-12/el-gobierno-extiende-a-toda-espana-la-recomendacion-de-cancelar-clases-y-celebrar-eventos-masivos-en-espacios-cerrados.html
Ban of large gatherings	2020-03-13	1.00	Castille and Leon	https://elpais.com/sociedad/2020-03-12/el-gobierno-extiende-a-toda-espana-la-recomendacion-de-cancelar-clases-y-celebrar-eventos-masivos-en-espacios-cerrados.html

NPI	Date	Cumulative share	Region	Source
Ban of large gatherings	2020-03-13	1.00	Valencian Community	https://elpais.com/sociedad/2020-03-12/el-gobierno-extiende-a-toda-espana-la-recomendacion-de-cancelar-clases-y-celebrar-eventos-masivos-en-espacios-cerrados.html
**NPI**	**Date**	**Cumulative share**	**Region**	**Source**
Ban of small gatherings	2020-03-15	1.00	Andalusia	https://www.cnbc.com/2020/03/14/spain-declares-state-of-emergency-due-to-coronavirus.html
Ban of small gatherings	2020-03-15	1.00	Navarre	https://www.cnbc.com/2020/03/14/spain-declares-state-of-emergency-due-to-coronavirus.html
Ban of small gatherings	2020-03-15	1.00	La Rioja	https://www.cnbc.com/2020/03/14/spain-declares-state-of-emergency-due-to-coronavirus.html
Ban of small gatherings	2020-03-15	1.00	Galicia	https://www.cnbc.com/2020/03/14/spain-declares-state-of-emergency-due-to-coronavirus.html
Ban of small gatherings	2020-03-15	1.00	Extremadura	https://www.cnbc.com/2020/03/14/spain-declares-state-of-emergency-due-to-coronavirus.html
Ban of small gatherings	2020-03-15	1.00	Community of Madrid	https://www.cnbc.com/2020/03/14/spain-declares-state-of-emergency-due-to-coronavirus.html
Ban of small gatherings	2020-03-15	1.00	Catalonia	https://www.cnbc.com/2020/03/14/spain-declares-state-of-emergency-due-to-coronavirus.html
Ban of small gatherings	2020-03-15	1.00	Region of Murcia	https://www.cnbc.com/2020/03/14/spain-declares-state-of-emergency-due-to-coronavirus.html
Ban of small gatherings	2020-03-15	1.00	Castille-La Mancha	https://www.cnbc.com/2020/03/14/spain-declares-state-of-emergency-due-to-coronavirus.html
Ban of small gatherings	2020-03-15	1.00	Cantabria	https://www.cnbc.com/2020/03/14/spain-declares-state-of-emergency-due-to-coronavirus.html

Ban of small gatherings	2020-03-15	1.00	Canary Islands	https://www.cnbc.com/2020/03/14/spain-declares-state-of-emergency-due-to-coronavirus.html
Ban of small gatherings	2020-03-15	1.00	Basque Country	https://www.cnbc.com/2020/03/14/spain-declares-state-of-emergency-due-to-coronavirus.html
Ban of small gatherings	2020-03-15	1.00	Balearic Islands	https://www.cnbc.com/2020/03/14/spain-declares-state-of-emergency-due-to-coronavirus.html
Ban of small gatherings	2020-03-15	1.00	Asturias	https://www.cnbc.com/2020/03/14/spain-declares-state-of-emergency-due-to-coronavirus.html
Ban of small gatherings	2020-03-15	1.00	Aragon	https://www.cnbc.com/2020/03/14/spain-declares-state-of-emergency-due-to-coronavirus.html
Ban of small gatherings	2020-03-15	1.00	Castille and Leon	https://www.cnbc.com/2020/03/14/spain-declares-state-of-emergency-due-to-coronavirus.html
Ban of small gatherings	2020-03-15	1.00	Valencian Community	https://www.cnbc.com/2020/03/14/spain-declares-state-of-emergency-due-to-coronavirus.html

**NPI**	**Date**	**Cumulative share**	**Region**	**Source**
School closure	2020-03-11	0.15	La Rioja	https://www.thestar.com.my/news/world/2020/03/10/spain039s-la-rioja-region-orders-schools-shutdown-as-coronavirus-spreads
School closure	2020-03-11	0.15	Community of Madrid	https://english.elpais.com/society/2020-03-09/madrid-basque-city-close-schools-as-coronavirus-continues-spread-in-spain.html
School closure	2020-03-13	0.46	Galicia	https://english.elpais.com/society/2020-03-12/basque-country-galicia-and-murcia-close-schools-in-bid-to-slow-coronavirus.html
School closure	2020-03-13	0.46	Basque Country	https://english.elpais.com/society/2020-03-12/basque-country-galicia-and-murcia-close-schools-in-bid-to-slow-coronavirus.html
School closure	2020-03-13	0.46	Canary Islands	https://english.elpais.com/society/2020-03-12/basque-country-galicia-and-murcia-close-schools-in-bid-to-slow-coronavirus.html

School closure	2020-03-13	0.46	Catalonia	https://english.elpais.com/society/2020-03-12/basque-country-galicia-and-murcia-close-schools-in-bid-to-slow-coronavirus.html
School closure	2020-03-16	1.00	Andalusia	https://english.elpais.com/society/2020-03-12/basque-country-galicia-and-murcia-close-schools-in-bid-to-slow-coronavirus.html
School closure	2020-03-16	1.00	Navarre	https://english.elpais.com/society/2020-03-12/basque-country-galicia-and-murcia-close-schools-in-bid-to-slow-coronavirus.html
School closure	2020-03-16	1.00	Extremadura	https://english.elpais.com/society/2020-03-12/basque-country-galicia-and-murcia-close-schools-in-bid-to-slow-coronavirus.html
School closure	2020-03-16	1.00	Castille-La Mancha	https://english.elpais.com/society/2020-03-12/basque-country-galicia-and-murcia-close-schools-in-bid-to-slow-coronavirus.html
School closure	2020-03-16	1.00	Castille and Leon	https://english.elpais.com/society/2020-03-12/basque-country-galicia-and-murcia-close-schools-in-bid-to-slow-coronavirus.html
School closure	2020-03-16	1.00	Cantabria	https://english.elpais.com/society/2020-03-12/basque-country-galicia-and-murcia-close-schools-in-bid-to-slow-coronavirus.html
School closure	2020-03-16	1.00	Balearic Islands	https://english.elpais.com/society/2020-03-12/basque-country-galicia-and-murcia-close-schools-in-bid-to-slow-coronavirus.html
School closure	2020-03-16	1.00	Asturias	https://english.elpais.com/society/2020-03-12/basque-country-galicia-and-murcia-close-schools-in-bid-to-slow-coronavirus.html
School closure	2020-03-16	1.00	Aragon	https://english.elpais.com/society/2020-03-12/basque-country-galicia-and-murcia-close-schools-in-bid-to-slow-coronavirus.html
School closure	2020-03-16	1.00	Region of Murcia	https://english.elpais.com/society/2020-03-12/basque-country-galicia-and-murcia-close-schools-in-bid-to-slow-coronavirus.html
School closure	2020-03-16	1.00	Valencian Community	https://english.elpais.com/society/2020-03-12/basque-country-galicia-and-murcia-close-schools-in-bid-to-slow-coronavirus.html
**NPI**	**Date**	**Cumulative share**	**Region**	**Source**

Venue closure	2020-03-13	0.29	Galicia	https://www.nytimes.com/aponline/2020/03/14/world/europe/ap-eu-virus-outbreak-spain.html
Venue closure	2020-03-13	0.29	Cantabria	https://www.eldiario.es/cantabria/ultima-hora/Cantabria-hosteleria-superficies-edificios-actividades_0_1005450439.html
Venue closure	2020-03-13	0.29	Castille and Leon	https://twitter.com/FranciscoIgea/status/1238571610468220929
Venue closure	2020-03-13	0.29	Catalonia	https://elpais.com/espana/catalunya/2020-03-13/cataluna-cierra-pistas-de-esqui-discotecas-y-areas-comerciales-que-no-sean-de-alimentacion.html
Venue closure	2020-03-14	0.56	Valencian Community	https://english.elpais.com/society/2020-03-13/madrid-orders-restaurants-and-bars-to-close-from-saturday-onward-to-slow-coronavirus-spread.html
Venue closure	2020-03-14	0.56	Asturias	https://cadenaser.com/emisora/2020/03/13/ser_gijon/1584138177_171951.html
Venue closure	2020-03-14	0.56	Community of Madrid	https://english.elpais.com/society/2020-03-13/madrid-orders-restaurants-and-bars-to-close-from-saturday-onward-to-slow-coronavirus-spread.html
Venue closure	2020-03-15	1.00	Navarre	https://www.cnbc.com/2020/03/14/spain-declares-state-of-emergency-due-to-coronavirus.html
Venue closure	2020-03-15	1.00	La Rioja	https://www.cnbc.com/2020/03/14/spain-declares-state-of-emergency-due-to-coronavirus.html
Venue closure	2020-03-15	1.00	Extremadura	https://www.cnbc.com/2020/03/14/spain-declares-state-of-emergency-due-to-coronavirus.html
Venue closure	2020-03-15	1.00	Andalusia	https://www.cnbc.com/2020/03/14/spain-declares-state-of-emergency-due-to-coronavirus.html
Venue closure	2020-03-15	1.00	Canary Islands	https://www.cnbc.com/2020/03/14/spain-declares-state-of-emergency-due-to-coronavirus.html

Venue closure	2020-03-15	1.00	Basque Country	https://www.cnbc.com/2020/03/14/spain-declares-state-of-emergency-due-to-coronavirus.html
Venue closure	2020-03-15	1.00	Balearic Islands	https://www.cnbc.com/2020/03/14/spain-declares-state-of-emergency-due-to-coronavirus.html
Venue closure	2020-03-15	1.00	Aragon	https://www.cnbc.com/2020/03/14/spain-declares-state-of-emergency-due-to-coronavirus.html
Venue closure	2020-03-15	1.00	Region of Murcia	https://www.cnbc.com/2020/03/14/spain-declares-state-of-emergency-due-to-coronavirus.html
Venue closure	2020-03-15	1.00	Castille-La Mancha	https://www.cnbc.com/2020/03/14/spain-declares-state-of-emergency-due-to-coronavirus.html

**NPI**	**Date**	**Cumulative share**	**Region**	**Source**
Lockdown	2020-03-15	1.00	Andalusia	https://www.cnbc.com/2020/03/14/spain-declares-state-of-emergency-due-to-coronavirus.html
Lockdown	2020-03-15	1.00	Navarre	https://www.cnbc.com/2020/03/14/spain-declares-state-of-emergency-due-to-coronavirus.html
Lockdown	2020-03-15	1.00	La Rioja	https://www.cnbc.com/2020/03/14/spain-declares-state-of-emergency-due-to-coronavirus.html
Lockdown	2020-03-15	1.00	Galicia	https://www.cnbc.com/2020/03/14/spain-declares-state-of-emergency-due-to-coronavirus.html
Lockdown	2020-03-15	1.00	Extremadura	https://www.cnbc.com/2020/03/14/spain-declares-state-of-emergency-due-to-coronavirus.html
Lockdown	2020-03-15	1.00	Community of Madrid	https://www.cnbc.com/2020/03/14/spain-declares-state-of-emergency-due-to-coronavirus.html

NPI	Date	Cumulative share	Region	Source
Lockdown	2020-03-15	1.00	Catalonia	https://www.cnbc.com/2020/03/14/spain-declares-state-of-emergency-due-to-coronavirus.html
Lockdown	2020-03-15	1.00	Region of Murcia	https://www.cnbc.com/2020/03/14/spain-declares-state-of-emergency-due-to-coronavirus.html
Lockdown	2020-03-15	1.00	Castille-La Mancha	https://www.cnbc.com/2020/03/14/spain-declares-state-of-emergency-due-to-coronavirus.html
Lockdown	2020-03-15	1.00	Cantabria	https://www.cnbc.com/2020/03/14/spain-declares-state-of-emergency-due-to-coronavirus.html
Lockdown	2020-03-15	1.00	Canary Islands	https://www.cnbc.com/2020/03/14/spain-declares-state-of-emergency-due-to-coronavirus.html
Lockdown	2020-03-15	1.00	Basque Country	https://www.cnbc.com/2020/03/14/spain-declares-state-of-emergency-due-to-coronavirus.html
Lockdown	2020-03-15	1.00	Balearic Islands	https://www.cnbc.com/2020/03/14/spain-declares-state-of-emergency-due-to-coronavirus.html
Lockdown	2020-03-15	1.00	Asturias	https://www.cnbc.com/2020/03/14/spain-declares-state-of-emergency-due-to-coronavirus.html
Lockdown	2020-03-15	1.00	Aragon	https://www.cnbc.com/2020/03/14/spain-declares-state-of-emergency-due-to-coronavirus.html
Lockdown	2020-03-15	1.00	Castille and Leon	https://www.cnbc.com/2020/03/14/spain-declares-state-of-emergency-due-to-coronavirus.html
Lockdown	2020-03-15	1.00	Valencian Community	https://www.cnbc.com/2020/03/14/spain-declares-state-of-emergency-due-to-coronavirus.html

Work-from-home order	2020-03-30	1.00	Andalusia	https://elpais.com/espana/2020-03-28/el-gobierno-amplia-el-confinamiento-los-trabajadores-de-actividades-no-esenciales-deberan-quedarse-en-casa.html
Work-from-home order	2020-03-30	1.00	Navarre	https://elpais.com/espana/2020-03-28/el-gobierno-amplia-el-confinamiento-los-trabajadores-de-actividades-no-esenciales-deberan-quedarse-en-casa.html
Work-from-home order	2020-03-30	1.00	La Rioja	https://elpais.com/espana/2020-03-28/el-gobierno-amplia-el-confinamiento-los-trabajadores-de-actividades-no-esenciales-deberan-quedarse-en-casa.html
Work-from-home order	2020-03-30	1.00	Galicia	https://elpais.com/espana/2020-03-28/el-gobierno-amplia-el-confinamiento-los-trabajadores-de-actividades-no-esenciales-deberan-quedarse-en-casa.html
Work-from-home order	2020-03-30	1.00	Extremadura	https://elpais.com/espana/2020-03-28/el-gobierno-amplia-el-confinamiento-los-trabajadores-de-actividades-no-esenciales-deberan-quedarse-en-casa.html
Work-from-home order	2020-03-30	1.00	Community of Madrid	https://elpais.com/espana/2020-03-28/el-gobierno-amplia-el-confinamiento-los-trabajadores-de-actividades-no-esenciales-deberan-quedarse-en-casa.html
Work-from-home order	2020-03-30	1.00	Catalonia	https://elpais.com/espana/2020-03-28/el-gobierno-amplia-el-confinamiento-los-trabajadores-de-actividades-no-esenciales-deberan-quedarse-en-casa.html
Work-from-home order	2020-03-30	1.00	Region of Murcia	https://elpais.com/espana/2020-03-28/el-gobierno-amplia-el-confinamiento-los-trabajadores-de-actividades-no-esenciales-deberan-quedarse-en-casa.html

Work-from-home order	2020-03-30	1.00	Castille-La Mancha	https://elpais.com/espana/2020-03-28/el-gobierno-amplia-el-confinamiento-los-trabajadores-de-actividades-no-esenciales-deberan-quedarse-en-casa.html
Work-from-home order	2020-03-30	1.00	Cantabria	https://elpais.com/espana/2020-03-28/el-gobierno-amplia-el-confinamiento-los-trabajadores-de-actividades-no-esenciales-deberan-quedarse-en-casa.html
Work-from-home order	2020-03-30	1.00	Canary Islands	https://elpais.com/espana/2020-03-28/el-gobierno-amplia-el-confinamiento-los-trabajadores-de-actividades-no-esenciales-deberan-quedarse-en-casa.html
Work-from-home order	2020-03-30	1.00	Basque Country	https://elpais.com/espana/2020-03-28/el-gobierno-amplia-el-confinamiento-los-trabajadores-de-actividades-no-esenciales-deberan-quedarse-en-casa.html
Work-from-home order	2020-03-30	1.00	Balearic Islands	https://elpais.com/espana/2020-03-28/el-gobierno-amplia-el-confinamiento-los-trabajadores-de-actividades-no-esenciales-deberan-quedarse-en-casa.html
Work-from-home order	2020-03-30	1.00	Asturias	https://elpais.com/espana/2020-03-28/el-gobierno-amplia-el-confinamiento-los-trabajadores-de-actividades-no-esenciales-deberan-quedarse-en-casa.html
Work-from-home order	2020-03-30	1.00	Aragon	https://elpais.com/espana/2020-03-28/el-gobierno-amplia-el-confinamiento-los-trabajadores-de-actividades-no-esenciales-deberan-quedarse-en-casa.html
Work-from-home order	2020-03-30	1.00	Castille and Leon	https://elpais.com/espana/2020-03-28/el-gobierno-amplia-el-confinamiento-los-trabajadores-de-actividades-no-esenciales-deberan-quedarse-en-casa.html

Work-from-home order	2020-03-30	1.00	Valencian Community	https://elpais.com/espana/2020-03-28/el-gobierno-amplia-el-confinamiento-los-trabajadores-de-actividades-no-esenciales-deberan-quedarse-en-casa.html

Table 9. Sources for policies implemented across different Spanish regions

NPI	Date	Cumulative share	Region	Source
Ban of large gatherings	2020-03-12	0.27	Alberta	https://www.alberta.ca/release.cfm?xID=6980324A5B1B0-BC2C-40A8-A6AD9E30E3189425
Ban of large gatherings	2020-03-12	0.27	British Columbia	https://news.gov.bc.ca/releases/2020HLTH0077-000484
Ban of large gatherings	2020-03-12	0.27	New Brunswick	https://www2.gnb.ca/content/gnb/en/news/news_release.2020.03.0114.html
Ban of large gatherings	2020-03-13	0.71	Manitoba	https://news.gov.mb.ca/news/index.html?item=46933&posted=2020-03-13
Ban of large gatherings	2020-03-13	0.71	Newfoundland and Labrador	https://www.gov.nl.ca/releases/2020/tcii/0313n04/
Ban of large gatherings	2020-03-13	0.71	Nunavut	https://www.gov.nu.ca/health/news/government-nunavut-response-covid-19
Ban of large gatherings	2020-03-13	0.71	Ontario	https://news.ontario.ca/mtc/en/2020/03/statement-from-minister-elliott-and-minister-macleod-on-the-2019-novel-coronavirus-covid-19-1.html
Ban of large gatherings	2020-03-14	0.94	Quebec	http://www.fil-information.gouv.qc.ca/Pages/Article.aspx?lang=en&motsCles=Covid&listeThe=&listeReg=&listeDiff=&type=&dateDebut=2019-09-28&dateFin=2020-03-28&afficherResultats=oui&Page=5&idArticle=2803149905
Ban of large gatherings	2020-03-15	0.96	Nova Scotia	https://novascotia.ca/news/release/?id=20200315002
Ban of large gatherings	2020-03-16	1.00	Prince Edward Island	https://www.princeedwardisland.ca/en/news/premier-announces-initial-financial-support-declares-public-health-emergency
Ban of large gatherings	2020-03-16	1.00	Saskatchewan	https://www.saskatchewan.ca/government/news-and-media/2020/march/13/further-measures-for-covid-19

Ban of large gatherings	2020-03-16	1.00	Yukon	https://yukon.ca/en/news/chief-medical-officer-health-recommends-broad-new-measures-yukon
Ban of large gatherings	2020-03-22	1.00	Northwest Territories	https://www.hss.gov.nt.ca/en/newsroom/all-gatherings-are-advised-cancel-effective-immediately

**NPI**	**Date**	**Cumulative share**	**Region**	**Source**
Ban of small gatherings	2020-03-16	0.00	Prince Edward Island	https://www.princeedwardisland.ca/en/news/premier-announces-initial-financial-support-declares-public-health-emergency
Ban of small gatherings	2020-03-19	0.02	New Brunswick	https://www2.gnb.ca/content/gnb/en/news/news_release.2020.03.0139.html
Ban of small gatherings	2020-03-20	0.06	Saskatchewan	https://www.saskatchewan.ca/government/news-and-media/2020/march/20/covid-19-update-march-20
Ban of small gatherings	2020-03-21	0.28	Quebec	http://www.fil-information.gouv.qc.ca/Pages/Article.aspx?lang=en&motsCles=Covid&listeThe=&listeReg=&listeDiff=&type=&dateDebut=2019-09-28&dateFin=2020-03-28&afficherResultats=oui&Page=2&idArticle=2803211636
Ban of small gatherings	2020-03-22	0.31	Nova Scotia	https://dailyhive.com/vancouver/nova-scotia-coronavirus-state-of-emergency
Ban of small gatherings	2020-03-22	0.31	Yukon	https://yukon.ca/en/news/yukons-chief-medical-officer-health-provides-update-covid-19-0
Ban of small gatherings	2020-03-24	0.31	Nunavut	https://www.gov.nu.ca/health/news/chief-public-health-officer-orders-prohibition-travel-nunavut-limited-exceptions
Ban of small gatherings	2020-03-27	0.43	Alberta	https://www.cbc.ca/news/canada/edmonton/alberta-covid-19-coronavirus-deena-hinshaw-1.5512445
Ban of small gatherings	2020-03-28	0.81	Ontario	https://globalnews.ca/news/6746181/ontario-ban-gatherings/

Ban of small gatherings	2020-03-31	0.83	Newfoundland and Labrador	https://www.gov.nl.ca/covid-19/faqs/
Ban of small gatherings	2020-04-01	0.86	Manitoba	https://news.gov.mb.ca/news/index.html?item=47337&posted=2020-03-30
Ban of small gatherings	2020-04-11	0.87	Northwest Territories	https://www.gov.nt.ca/en/newsroom/two-new-orders-nwt-chief-public-health-officer-strengthen-response-covid-19-pandemic

**NPI**	**Date**	**Cumulative share**	**Region**	**Source**
School closure	2020-03-13	0.02	New Brunswick	https://www2.gnb.ca/content/gnb/en/news/news_release.2020.03.0117.html
School closure	2020-03-14	0.25	Quebec	http://www.fil-information.gouv.qc.ca/Pages/Article.aspx?lang=en&motsCles=Covid&listeThe=&listeReg=&listeDiff=&type=&dateDebut=2019-09-28&dateFin=2020-03-28&afficherResultats=oui&Page=6&idArticle=2803137507
School closure	2020-03-15	0.36	Alberta	https://www.alberta.ca/release.cfm?xID=69818C355F188-C2A3-F5C6-875A2A33929D5C05
School closure	2020-03-16	0.80	Newfoundland and Labrador	https://www.gov.nl.ca/releases/2020/eecd/0316n04/
School closure	2020-03-16	0.80	Northwest Territories	https://cabinradio.ca/32133/news/education/nwt-tells-schools-to-close-until-after-easter-daycares-unaffected/
School closure	2020-03-16	0.80	Ontario	https://news.ontario.ca/maesd/en/2020/03/statement-from-minister-elliott-and-minister-romano-on-the-2019-novel-coronavirus-covid-19.html
School closure	2020-03-16	0.80	Saskatchewan	https://www.saskatchewan.ca/government/news-and-media/2020/march/16/class-suspensions
School closure	2020-03-17	0.96	British Columbia	https://news.gov.bc.ca/releases/2020EMBC0014-000552

School closure	2020-03-17	0.96	Nova Scotia	https://novascotia.ca/news/release/?id=20200315002
School closure	2020-03-17	0.96	Nunavut	https://www.gov.nu.ca/health/news/temporary-nunavut-wide-school-and-daycare-closures-precaution-covid-19
School closure	2020-03-17	0.96	Prince Edward Island	https://www.princeedwardisland.ca/en/news/province-announces-covid-19-related-closures
School closure	2020-03-18	0.96	Yukon	https://yukon.ca/en/news/chief-medical-officer-health-declares-public-health-emergency
School closure	2020-03-23	1.00	Manitoba	https://news.gov.mb.ca/news/index.html?item=46936&posted=2020-03-14

**NPI**	**Date**	**Cumulative share**	**Region**	**Source**
Venue closure	2020-03-15	0.23	Quebec	http://www.fil-information.gouv.qc.ca/Pages/Article.aspx?lang=en&motsCles=Covid&listeThe=&listeReg=&listeDiff=&type=&dateDebut=2019-09-28&dateFin=2020-03-28&afficherResultats=oui&Page=5&idArticle=2803159403
Venue closure	2020-03-16	0.61	Ontario	https://news.ontario.ca/mohltc/en/2020/03/enhanced-measures-to-protect-ontarians-from-covid-19.html
Venue closure	2020-03-17	0.89	Alberta	https://www.alberta.ca/release.cfm?xID=69828242A5FFC-D75A-C83E-690D8028C0C4E09F
Venue closure	2020-03-17	0.89	British Columbia	https://news.gov.bc.ca/releases/2020EMBC0014-000552
Venue closure	2020-03-17	0.89	New Brunswick	https://www2.gnb.ca/content/gnb/en/news/news_release.2020.03.0127.html
Venue closure	2020-03-17	0.89	Prince Edward Island	https://www.princeedwardisland.ca/en/news/chief-public-health-officer-urges-islanders-work-together-reduce-spread-covid-19

Venue closure	2020-03-19	0.92	Nova Scotia	https://novascotia.ca/news/release/?id=20200317005
Venue closure	2020-03-20	0.95	Nunavut	https://www.gov.nu.ca/health/news/minister-health-declares-public-health-emergency
Venue closure	2020-03-20	0.95	Saskatchewan	https://www.saskatchewan.ca/government/news-and-media/2020/march/20/covid-19-update-march-20
Venue closure	2020-03-22	0.95	Northwest Territories	https://www.hss.gov.nt.ca/en/newsroom/all-gatherings-are-advised-cancel-effective-immediately
Venue closure	2020-03-23	0.96	Newfoundland and Labrador	https://www.gov.nl.ca/covid-19/
Venue closure	2020-03-25	0.96	Yukon	https://yukon.ca/en/news/yukons-chief-medical-officer-health-provides-update-covid-19-0
Venue closure	2020-04-01	1.00	Manitoba	https://news.gov.mb.ca/news/index.html?item=47337&posted=2020-03-30

**NPI**	**Date**	**Cumulative share**	**Region**	**Source**
Lockdown	2020-03-29	0.02	New Brunswick	https://www2.gnb.ca/content/gnb/en/news/news_release.2020.03.0164.html
Lockdown	2020-03-30	0.41	Ontario	https://news.ontario.ca/mohltc/en/2020/03/statement-from-the-chief-medical-officer-of-health.html

**NPI**	**Date**	**Cumulative share**	**Region**	**Source**
Work-from-home order	2020-03-24	0.39	Ontario	https://news.ontario.ca/opo/en/2020/03/ontario-closing-at-risk-workplaces-to-protect-health-and-safety.html
Work-from-home order	2020-03-27	0.39	Prince Edward Island	https://www.princeedwardisland.ca/en/news/prince-edward-island-extends-closures-for-schools-daycares-non-essential-services

Work-from-home order	2020-04-01	0.43	Manitoba	https://news.gov.mb.ca/news/index.html?item=47337&posted=2020-03-30

Table 10. Sources for policies implemented across different Canadian regions

NPI	Date	Cumulative share	Region	Source
Ban of large gatherings	2020-03-16	1.00	Western Australia	https://www.watoday.com.au/politics/federal/effective-ban-on-non-essential-mass-gatherings-of-500-people-20200313-p549u5.html
Ban of large gatherings	2020-03-16	1.00	Northern Territory	https://www.watoday.com.au/politics/federal/effective-ban-on-non-essential-mass-gatherings-of-500-people-20200313-p549u5.html
Ban of large gatherings	2020-03-16	1.00	South Australia	https://www.watoday.com.au/politics/federal/effective-ban-on-non-essential-mass-gatherings-of-500-people-20200313-p549u5.html
Ban of large gatherings	2020-03-16	1.00	Queensland	https://www.watoday.com.au/politics/federal/effective-ban-on-non-essential-mass-gatherings-of-500-people-20200313-p549u5.html
Ban of large gatherings	2020-03-16	1.00	New South Wales	https://www.watoday.com.au/politics/federal/effective-ban-on-non-essential-mass-gatherings-of-500-people-20200313-p549u5.html
Ban of large gatherings	2020-03-16	1.00	Victoria	https://www.watoday.com.au/politics/federal/effective-ban-on-non-essential-mass-gatherings-of-500-people-20200313-p549u5.html
Ban of large gatherings	2020-03-16	1.00	Tasmania	https://www.watoday.com.au/politics/federal/effective-ban-on-non-essential-mass-gatherings-of-500-people-20200313-p549u5.html
Ban of large gatherings	2020-03-16	1.00	Australian Capital Territory	https://www.watoday.com.au/politics/federal/effective-ban-on-non-essential-mass-gatherings-of-500-people-20200313-p549u5.html

NPI	Date	Cumulative share	Region	Source
Ban of small gatherings	2020-03-23	0.01	Northern Territory	https://coronavirus.nt.gov.au/updates
Ban of small gatherings	2020-03-28	0.08	South Australia	https://www.sa.gov.au/__data/assets/pdf_file/0003/605055/Emergency-Management-GatheringsCOVID-19-Direction-2020_FINAL.pdf

Ban of small gatherings	2020-03-29	0.90	Queensland	https://www.health.gov.au/news/health-alerts/novel-coronavirus-2019-ncov-health-alert/how-to-protect-yourself-and-others-from-coronavirus-covid-19/limits-on-public-gatherings-for-coronavirus-covid-19
Ban of small gatherings	2020-03-29	0.90	New South Wales	https://www.health.gov.au/news/health-alerts/novel-coronavirus-2019-ncov-health-alert/how-to-protect-yourself-and-others-from-coronavirus-covid-19/limits-on-public-gatherings-for-coronavirus-covid-19
Ban of small gatherings	2020-03-29	0.90	Victoria	https://www.health.gov.au/news/health-alerts/novel-coronavirus-2019-ncov-health-alert/how-to-protect-yourself-and-others-from-coronavirus-covid-19/limits-on-public-gatherings-for-coronavirus-covid-19
Ban of small gatherings	2020-03-29	0.90	Tasmania	https://www.health.gov.au/news/health-alerts/novel-coronavirus-2019-ncov-health-alert/how-to-protect-yourself-and-others-from-coronavirus-covid-19/limits-on-public-gatherings-for-coronavirus-covid-19
Ban of small gatherings	2020-03-29	0.90	Australian Capital Territory	https://www.health.gov.au/news/health-alerts/novel-coronavirus-2019-ncov-health-alert/how-to-protect-yourself-and-others-from-coronavirus-covid-19/limits-on-public-gatherings-for-coronavirus-covid-19
Ban of small gatherings	2020-04-01	1.00	Western Australia	https://ww2.health.wa.gov.au/~/media/Files/Corporate/general%20documents/Infectious%20diseases/PDF/Coronavirus/COVID19-Agency-Advisory-14-2020.pdf

**NPI**	**Date**	**Cumulative share**	**Region**	**Source**
School closure	2020-03-16	0.32	New South Wales	https://www.abc.net.au/news/2020-03-23/coronavirus-parents-told-to-keep-children-home-from-school/12079524
School closure	2020-03-23	0.60	Victoria	https://www.theguardian.com/world/2020/mar/22/victoria-nsw-lockdowns-scott-morrison-coronavirus-national-cabinet
School closure	2020-03-23	0.60	Australian Capital Territory	https://www.theguardian.com/world/2020/mar/22/victoria-nsw-lockdowns-scott-morrison-coronavirus-national-cabinet

School closure	2020-04-20	0.80	Queensland	https://www.theguardian.com/australia-news/2020/apr/13/are-schools-open-closed-term-2-australia-coronavirus-easter-holidays

NPI	Date	Cumulative share	Region	Source
Venue closure	2020-03-23	1.00	Western Australia	https://www.theguardian.com/world/live/2020/mar/22/coronavirus-updates-live-australia-nsw-victoria-qld-tasmania-cases-government-stimulus-latest-update-news
Venue closure	2020-03-23	1.00	Northern Territory	https://www.theguardian.com/world/live/2020/mar/22/coronavirus-updates-live-australia-nsw-victoria-qld-tasmania-cases-government-stimulus-latest-update-news
Venue closure	2020-03-23	1.00	South Australia	https://www.theguardian.com/world/live/2020/mar/22/coronavirus-updates-live-australia-nsw-victoria-qld-tasmania-cases-government-stimulus-latest-update-news
Venue closure	2020-03-23	1.00	Queensland	https://www.theguardian.com/world/live/2020/mar/22/coronavirus-updates-live-australia-nsw-victoria-qld-tasmania-cases-government-stimulus-latest-update-news
Venue closure	2020-03-23	1.00	New South Wales	https://www.theguardian.com/world/live/2020/mar/22/coronavirus-updates-live-australia-nsw-victoria-qld-tasmania-cases-government-stimulus-latest-update-news
Venue closure	2020-03-23	1.00	Victoria	https://www.theguardian.com/world/live/2020/mar/22/coronavirus-updates-live-australia-nsw-victoria-qld-tasmania-cases-government-stimulus-latest-update-news
Venue closure	2020-03-23	1.00	Tasmania	https://www.theguardian.com/world/live/2020/mar/22/coronavirus-updates-live-australia-nsw-victoria-qld-tasmania-cases-government-stimulus-latest-update-news

Venue closure	2020-03-23	1.00	Australian Capital Territory	https://www.theguardian.com/world/live/2020/mar/22/coronavirus-updates-live-australia-nsw-victoria-qld-tasmania-cases-government-stimulus-latest-update-news

NPI	Date	Cumulative share	Region	Source
Lockdown	2020-03-29	0.02	Australian Capital Territory	https://www.covid19.act.gov.au/news-articles/latest-federal-government-announcement
Lockdown	2020-03-31	0.62	New South Wales	https://www.legislation.nsw.gov.au/_emergency/Public%20Health%20(COVID-19%20Restrictions%20on%20Gathering%20and%20Movement)%20Order%202020.pdf
Lockdown	2020-03-31	0.62	Victoria	https://www.vic.gov.au/coronavirusresponse
Lockdown	2020-03-31	0.62	Tasmania	http://www.premier.tas.gov.au/releases/keeping_tasmanians_safe_and_secure_stay_home,_save_lives
Lockdown	2020-04-02	0.82	Queensland	https://www.health.qld.gov.au/system-governance/legislation/cho-public-health-directions-under-expanded-public-health-act-powers/home-confinement-movement-gathering-direction

Table 11. Sources for policies implemented across different Australian regions

NPI	Date	Cumulative share	Region	Source
Ban of large gatherings	2020-03-02	0.32	Lombardia	https://www.gazzettaufficiale.it/eli/id/2020/03/01/20A01381/sg
Ban of large gatherings	2020-03-02	0.32	Veneto	https://www.gazzettaufficiale.it/eli/id/2020/03/01/20A01381/sg
Ban of large gatherings	2020-03-02	0.32	Emilia-Romagna	https://www.gazzettaufficiale.it/eli/id/2020/03/01/20A01381/sg
Ban of large gatherings	2020-03-08	1.00	Trentino-South Tyrol	https://www.gazzettaufficiale.it/showNewsDetail?id=2513&backTo=archivio&anno=2020&provenienza=archivio
Ban of large gatherings	2020-03-08	1.00	Tuscany	https://www.gazzettaufficiale.it/showNewsDetail?id=2513&backTo=archivio&anno=2020&provenienza=archivio
Ban of large gatherings	2020-03-08	1.00	Sicily	https://www.gazzettaufficiale.it/showNewsDetail?id=2513&backTo=archivio&anno=2020&provenienza=archivio
Ban of large gatherings	2020-03-08	1.00	Sardinia	https://www.gazzettaufficiale.it/showNewsDetail?id=2513&backTo=archivio&anno=2020&provenienza=archivio
Ban of large gatherings	2020-03-08	1.00	Puglia (Apulia)	https://www.gazzettaufficiale.it/showNewsDetail?id=2513&backTo=archivio&anno=2020&provenienza=archivio
Ban of large gatherings	2020-03-08	1.00	Piemonte	https://www.gazzettaufficiale.it/showNewsDetail?id=2513&backTo=archivio&anno=2020&provenienza=archivio
Ban of large gatherings	2020-03-08	1.00	Molise	https://www.gazzettaufficiale.it/showNewsDetail?id=2513&backTo=archivio&anno=2020&provenienza=archivio
Ban of large gatherings	2020-03-08	1.00	Liguria	https://www.gazzettaufficiale.it/showNewsDetail?id=2513&backTo=archivio&anno=2020&provenienza=archivio

Ban of large gatherings	2020-03-08	1.00	Umbria	https://www.gazzettaufficiale.it/showNewsDetail?id=2513&backTo=archivio&anno=2020&provenienza=archivio
Ban of large gatherings	2020-03-08	1.00	Lazio	https://www.gazzettaufficiale.it/showNewsDetail?id=2513&backTo=archivio&anno=2020&provenienza=archivio
Ban of large gatherings	2020-03-08	1.00	Friuli-Venezia Giulia	https://www.gazzettaufficiale.it/showNewsDetail?id=2513&backTo=archivio&anno=2020&provenienza=archivio
Ban of large gatherings	2020-03-08	1.00	Campania	https://www.gazzettaufficiale.it/showNewsDetail?id=2513&backTo=archivio&anno=2020&provenienza=archivio
Ban of large gatherings	2020-03-08	1.00	Calabria	https://www.gazzettaufficiale.it/showNewsDetail?id=2513&backTo=archivio&anno=2020&provenienza=archivio
Ban of large gatherings	2020-03-08	1.00	Basilicata	https://www.gazzettaufficiale.it/showNewsDetail?id=2513&backTo=archivio&anno=2020&provenienza=archivio
Ban of large gatherings	2020-03-08	1.00	Abruzzo	https://www.gazzettaufficiale.it/showNewsDetail?id=2513&backTo=archivio&anno=2020&provenienza=archivio
Ban of large gatherings	2020-03-08	1.00	Marche	https://www.gazzettaufficiale.it/showNewsDetail?id=2513&backTo=archivio&anno=2020&provenienza=archivio
Ban of large gatherings	2020-03-08	1.00	Aosta Valley	https://www.gazzettaufficiale.it/showNewsDetail?id=2513&backTo=archivio&anno=2020&provenienza=archivio

**NPI**	**Date**	**Cumulative share**	**Region**	**Source**
Ban of small gatherings	2020-03-02	0.32	Lombardia	https://www.gazzettaufficiale.it/eli/id/2020/03/01/20A01381/sg
Ban of small gatherings	2020-03-02	0.32	Veneto	https://www.gazzettaufficiale.it/eli/id/2020/03/01/20A01381/sg
Ban of small gatherings	2020-03-02	0.32	Emilia-Romagna	https://www.gazzettaufficiale.it/eli/id/2020/03/01/20A01381/sg

Ban of small gatherings	2020-03-08	1.00	Trentino-South Tyrol	https://www.gazzettaufficiale.it/showNewsDetail?id=2513&backTo=archivio&anno=2020&provenienza=archivio
Ban of small gatherings	2020-03-08	1.00	Tuscany	https://www.gazzettaufficiale.it/showNewsDetail?id=2513&backTo=archivio&anno=2020&provenienza=archivio
Ban of small gatherings	2020-03-08	1.00	Sicily	https://www.gazzettaufficiale.it/showNewsDetail?id=2513&backTo=archivio&anno=2020&provenienza=archivio
Ban of small gatherings	2020-03-08	1.00	Sardinia	https://www.gazzettaufficiale.it/showNewsDetail?id=2513&backTo=archivio&anno=2020&provenienza=archivio
Ban of small gatherings	2020-03-08	1.00	Puglia (Apulia)	https://www.gazzettaufficiale.it/showNewsDetail?id=2513&backTo=archivio&anno=2020&provenienza=archivio
Ban of small gatherings	2020-03-08	1.00	Piemonte	https://www.gazzettaufficiale.it/showNewsDetail?id=2513&backTo=archivio&anno=2020&provenienza=archivio
Ban of small gatherings	2020-03-08	1.00	Molise	https://www.gazzettaufficiale.it/showNewsDetail?id=2513&backTo=archivio&anno=2020&provenienza=archivio
Ban of small gatherings	2020-03-08	1.00	Liguria	https://www.gazzettaufficiale.it/showNewsDetail?id=2513&backTo=archivio&anno=2020&provenienza=archivio
Ban of small gatherings	2020-03-08	1.00	Umbria	https://www.gazzettaufficiale.it/showNewsDetail?id=2513&backTo=archivio&anno=2020&provenienza=archivio
Ban of small gatherings	2020-03-08	1.00	Lazio	https://www.gazzettaufficiale.it/showNewsDetail?id=2513&backTo=archivio&anno=2020&provenienza=archivio
Ban of small gatherings	2020-03-08	1.00	Friuli-Venezia Giulia	https://www.gazzettaufficiale.it/showNewsDetail?id=2513&backTo=archivio&anno=2020&provenienza=archivio
Ban of small gatherings	2020-03-08	1.00	Campania	https://www.gazzettaufficiale.it/showNewsDetail?id=2513&backTo=archivio&anno=2020&provenienza=archivio
Ban of small gatherings	2020-03-08	1.00	Calabria	https://www.gazzettaufficiale.it/showNewsDetail?id=2513&backTo=archivio&anno=2020&provenienza=archivio

Ban of small gatherings	2020-03-08	1.00	Basilicata	https://www.gazzettaufficiale.it/showNewsDetail?id=2513&backTo=archivio&anno=2020&provenienza=archivio
Ban of small gatherings	2020-03-08	1.00	Abruzzo	https://www.gazzettaufficiale.it/showNewsDetail?id=2513&backTo=archivio&anno=2020&provenienza=archivio
Ban of small gatherings	2020-03-08	1.00	Marche	https://www.gazzettaufficiale.it/showNewsDetail?id=2513&backTo=archivio&anno=2020&provenienza=archivio
Ban of small gatherings	2020-03-08	1.00	Aosta Valley	https://www.gazzettaufficiale.it/showNewsDetail?id=2513&backTo=archivio&anno=2020&provenienza=archivio

**NPI**	**Date**	**Cumulative share**	**Region**	**Source**
School closure	2020-03-02	0.32	Lombardia	https://www.gazzettaufficiale.it/eli/id/2020/03/01/20A01381/sg
School closure	2020-03-02	0.32	Veneto	https://www.gazzettaufficiale.it/eli/id/2020/03/01/20A01381/sg
School closure	2020-03-02	0.32	Emilia-Romagna	https://www.gazzettaufficiale.it/eli/id/2020/03/01/20A01381/sg
School closure	2020-03-05	1.00	Trentino-South Tyrol	https://www.gazzettaufficiale.it/eli/id/2020/03/04/20A01475/sg
School closure	2020-03-05	1.00	Tuscany	https://www.gazzettaufficiale.it/eli/id/2020/03/04/20A01475/sg
School closure	2020-03-05	1.00	Sicily	https://www.gazzettaufficiale.it/eli/id/2020/03/04/20A01475/sg
School closure	2020-03-05	1.00	Sardinia	https://www.gazzettaufficiale.it/eli/id/2020/03/04/20A01475/sg
School closure	2020-03-05	1.00	Puglia (Apulia)	https://www.gazzettaufficiale.it/eli/id/2020/03/04/20A01475/sg

NPI	Date	Cumulative share	Region	Source
School closure	2020-03-05	1.00	Piemonte	https://www.gazzettaufficiale.it/eli/id/2020/03/04/20A01475/sg
School closure	2020-03-05	1.00	Molise	https://www.gazzettaufficiale.it/eli/id/2020/03/04/20A01475/sg
School closure	2020-03-05	1.00	Liguria	https://www.gazzettaufficiale.it/eli/id/2020/03/04/20A01475/sg
School closure	2020-03-05	1.00	Umbria	https://www.gazzettaufficiale.it/eli/id/2020/03/04/20A01475/sg
School closure	2020-03-05	1.00	Lazio	https://www.gazzettaufficiale.it/eli/id/2020/03/04/20A01475/sg
School closure	2020-03-05	1.00	Friuli-Venezia Giulia	https://www.gazzettaufficiale.it/eli/id/2020/03/04/20A01475/sg
School closure	2020-03-05	1.00	Campania	https://www.gazzettaufficiale.it/eli/id/2020/03/04/20A01475/sg
School closure	2020-03-05	1.00	Calabria	https://www.gazzettaufficiale.it/eli/id/2020/03/04/20A01475/sg
School closure	2020-03-05	1.00	Basilicata	https://www.gazzettaufficiale.it/eli/id/2020/03/04/20A01475/sg
School closure	2020-03-05	1.00	Abruzzo	https://www.gazzettaufficiale.it/eli/id/2020/03/04/20A01475/sg
School closure	2020-03-05	1.00	Marche	https://www.gazzettaufficiale.it/eli/id/2020/03/04/20A01475/sg
School closure	2020-03-05	1.00	Aosta Valley	https://www.gazzettaufficiale.it/eli/id/2020/03/04/20A01475/sg

Venue closure		2020-03-12	1.00	Lombardia	https://www.gazzettaufficiale.it/showNewsDetail?id=2532&backTo=archivio&anno=2020&provenienza=archivio
Venue closure		2020-03-12	1.00	Trentino-South Tyrol	https://www.gazzettaufficiale.it/showNewsDetail?id=2532&backTo=archivio&anno=2020&provenienza=archivio
Venue closure		2020-03-12	1.00	Tuscany	https://www.gazzettaufficiale.it/showNewsDetail?id=2532&backTo=archivio&anno=2020&provenienza=archivio
Venue closure		2020-03-12	1.00	Sicily	https://www.gazzettaufficiale.it/showNewsDetail?id=2532&backTo=archivio&anno=2020&provenienza=archivio
Venue closure		2020-03-12	1.00	Sardinia	https://www.gazzettaufficiale.it/showNewsDetail?id=2532&backTo=archivio&anno=2020&provenienza=archivio
Venue closure		2020-03-12	1.00	Puglia (Apulia)	https://www.gazzettaufficiale.it/showNewsDetail?id=2532&backTo=archivio&anno=2020&provenienza=archivio
Venue closure		2020-03-12	1.00	Piemonte	https://www.gazzettaufficiale.it/showNewsDetail?id=2532&backTo=archivio&anno=2020&provenienza=archivio
Venue closure		2020-03-12	1.00	Molise	https://www.gazzettaufficiale.it/showNewsDetail?id=2532&backTo=archivio&anno=2020&provenienza=archivio
Venue closure		2020-03-12	1.00	Marche	https://www.gazzettaufficiale.it/showNewsDetail?id=2532&backTo=archivio&anno=2020&provenienza=archivio
Venue closure		2020-03-12	1.00	Liguria	https://www.gazzettaufficiale.it/showNewsDetail?id=2532&backTo=archivio&anno=2020&provenienza=archivio
Venue closure		2020-03-12	1.00	Lazio	https://www.gazzettaufficiale.it/showNewsDetail?id=2532&backTo=archivio&anno=2020&provenienza=archivio
Venue closure		2020-03-12	1.00	Friuli-Venezia Giulia	https://www.gazzettaufficiale.it/showNewsDetail?id=2532&backTo=archivio&anno=2020&provenienza=archivio
Venue closure		2020-03-12	1.00	Campania	https://www.gazzettaufficiale.it/showNewsDetail?id=2532&backTo=archivio&anno=2020&provenienza=archivio

Venue closure	2020-03-12	1.00	Calabria	https://www.gazzettaufficiale.it/showNewsDetail?id=2532&backTo=archivio&anno=2020&provenienza=archivio
Venue closure	2020-03-12	1.00	Basilicata	https://www.gazzettaufficiale.it/showNewsDetail?id=2532&backTo=archivio&anno=2020&provenienza=archivio
Venue closure	2020-03-12	1.00	Abruzzo	https://www.gazzettaufficiale.it/showNewsDetail?id=2532&backTo=archivio&anno=2020&provenienza=archivio
Venue closure	2020-03-12	1.00	Emilia-Romagna	https://www.gazzettaufficiale.it/showNewsDetail?id=2532&backTo=archivio&anno=2020&provenienza=archivio
Venue closure	2020-03-12	1.00	Veneto	https://www.gazzettaufficiale.it/showNewsDetail?id=2532&backTo=archivio&anno=2020&provenienza=archivio
Venue closure	2020-03-12	1.00	Umbria	https://www.gazzettaufficiale.it/showNewsDetail?id=2532&backTo=archivio&anno=2020&provenienza=archivio
Venue closure	2020-03-12	1.00	Aosta Valley	https://www.gazzettaufficiale.it/showNewsDetail?id=2532&backTo=archivio&anno=2020&provenienza=archivio

**NPI**	**Date**	**Cumulative share**	**Region**	**Source**
Lockdown	2020-03-12	1.00	Lombardia	https://www.gazzettaufficiale.it/showNewsDetail?id=2532&backTo=archivio&anno=2020&provenienza=archivio
Lockdown	2020-03-12	1.00	Trentino-South Tyrol	https://www.gazzettaufficiale.it/showNewsDetail?id=2532&backTo=archivio&anno=2020&provenienza=archivio
Lockdown	2020-03-12	1.00	Tuscany	https://www.gazzettaufficiale.it/showNewsDetail?id=2532&backTo=archivio&anno=2020&provenienza=archivio
Lockdown	2020-03-12	1.00	Sicily	https://www.gazzettaufficiale.it/showNewsDetail?id=2532&backTo=archivio&anno=2020&provenienza=archivio
Lockdown	2020-03-12	1.00	Sardinia	https://www.gazzettaufficiale.it/showNewsDetail?id=2532&backTo=archivio&anno=2020&provenienza=archivio

Lockdown	2020-03-12	1.00	Puglia (Apulia)	https://www.gazzettaufficiale.it/showNewsDetail?id=2532&backTo=archivio&anno=2020&provenienza=archivio
Lockdown	2020-03-12	1.00	Piemonte	https://www.gazzettaufficiale.it/showNewsDetail?id=2532&backTo=archivio&anno=2020&provenienza=archivio
Lockdown	2020-03-12	1.00	Molise	https://www.gazzettaufficiale.it/showNewsDetail?id=2532&backTo=archivio&anno=2020&provenienza=archivio
Lockdown	2020-03-12	1.00	Marche	https://www.gazzettaufficiale.it/showNewsDetail?id=2532&backTo=archivio&anno=2020&provenienza=archivio
Lockdown	2020-03-12	1.00	Liguria	https://www.gazzettaufficiale.it/showNewsDetail?id=2532&backTo=archivio&anno=2020&provenienza=archivio
Lockdown	2020-03-12	1.00	Lazio	https://www.gazzettaufficiale.it/showNewsDetail?id=2532&backTo=archivio&anno=2020&provenienza=archivio
Lockdown	2020-03-12	1.00	Friuli-Venezia Giulia	https://www.gazzettaufficiale.it/showNewsDetail?id=2532&backTo=archivio&anno=2020&provenienza=archivio
Lockdown	2020-03-12	1.00	Campania	https://www.gazzettaufficiale.it/showNewsDetail?id=2532&backTo=archivio&anno=2020&provenienza=archivio
Lockdown	2020-03-12	1.00	Calabria	https://www.gazzettaufficiale.it/showNewsDetail?id=2532&backTo=archivio&anno=2020&provenienza=archivio
Lockdown	2020-03-12	1.00	Basilicata	https://www.gazzettaufficiale.it/showNewsDetail?id=2532&backTo=archivio&anno=2020&provenienza=archivio
Lockdown	2020-03-12	1.00	Abruzzo	https://www.gazzettaufficiale.it/showNewsDetail?id=2532&backTo=archivio&anno=2020&provenienza=archivio
Lockdown	2020-03-12	1.00	Emilia-Romagna	https://www.gazzettaufficiale.it/showNewsDetail?id=2532&backTo=archivio&anno=2020&provenienza=archivio
Lockdown	2020-03-12	1.00	Veneto	https://www.gazzettaufficiale.it/showNewsDetail?id=2532&backTo=archivio&anno=2020&provenienza=archivio

Lockdown	2020-03-12	1.00	Umbria	https://www.gazzettaufficiale.it/showNewsDetail?id=2532&backTo=archivio&anno=2020&provenienza=archivio
Lockdown	2020-03-12	1.00	Aosta Valley	https://www.gazzettaufficiale.it/showNewsDetail?id=2532&backTo=archivio&anno=2020&provenienza=archivio

**NPI**	**Date**	**Cumulative share**	**Region**	**Source**
Work-from-home order	2020-03-22	1.00	Lombardia	https://www.gazzettaufficiale.it/showNewsDetail?id=2545&backTo=archivio&anno=2020&provenienza=archivio
Work-from-home order	2020-03-22	1.00	Trentino-South Tyrol	https://www.gazzettaufficiale.it/showNewsDetail?id=2545&backTo=archivio&anno=2020&provenienza=archivio
Work-from-home order	2020-03-22	1.00	Tuscany	https://www.gazzettaufficiale.it/showNewsDetail?id=2545&backTo=archivio&anno=2020&provenienza=archivio
Work-from-home order	2020-03-22	1.00	Sicily	https://www.gazzettaufficiale.it/showNewsDetail?id=2545&backTo=archivio&anno=2020&provenienza=archivio
Work-from-home order	2020-03-22	1.00	Sardinia	https://www.gazzettaufficiale.it/showNewsDetail?id=2545&backTo=archivio&anno=2020&provenienza=archivio
Work-from-home order	2020-03-22	1.00	Puglia (Apulia)	https://www.gazzettaufficiale.it/showNewsDetail?id=2545&backTo=archivio&anno=2020&provenienza=archivio
Work-from-home order	2020-03-22	1.00	Piemonte	https://www.gazzettaufficiale.it/showNewsDetail?id=2545&backTo=archivio&anno=2020&provenienza=archivio
Work-from-home order	2020-03-22	1.00	Molise	https://www.gazzettaufficiale.it/showNewsDetail?id=2545&backTo=archivio&anno=2020&provenienza=archivio
Work-from-home order	2020-03-22	1.00	Marche	https://www.gazzettaufficiale.it/showNewsDetail?id=2545&backTo=archivio&anno=2020&provenienza=archivio
Work-from-home order	2020-03-22	1.00	Liguria	https://www.gazzettaufficiale.it/showNewsDetail?id=2545&backTo=archivio&anno=2020&provenienza=archivio

Work-from-home order	2020-03-22	1.00	Lazio	https://www.gazzettaufficiale.it/showNewsDetail?id=2545&backTo=archivio&anno=2020&provenienza=archivio
Work-from-home order	2020-03-22	1.00	Friuli-Venezia Giulia	https://www.gazzettaufficiale.it/showNewsDetail?id=2545&backTo=archivio&anno=2020&provenienza=archivio
Work-from-home order	2020-03-22	1.00	Campania	https://www.gazzettaufficiale.it/showNewsDetail?id=2545&backTo=archivio&anno=2020&provenienza=archivio
Work-from-home order	2020-03-22	1.00	Calabria	https://www.gazzettaufficiale.it/showNewsDetail?id=2545&backTo=archivio&anno=2020&provenienza=archivio
Work-from-home order	2020-03-22	1.00	Basilicata	https://www.gazzettaufficiale.it/showNewsDetail?id=2545&backTo=archivio&anno=2020&provenienza=archivio
Work-from-home order	2020-03-22	1.00	Abruzzo	https://www.gazzettaufficiale.it/showNewsDetail?id=2545&backTo=archivio&anno=2020&provenienza=archivio
Work-from-home order	2020-03-22	1.00	Emilia-Romagna	https://www.gazzettaufficiale.it/showNewsDetail?id=2545&backTo=archivio&anno=2020&provenienza=archivio
Work-from-home order	2020-03-22	1.00	Veneto	https://www.gazzettaufficiale.it/showNewsDetail?id=2545&backTo=archivio&anno=2020&provenienza=archivio
Work-from-home order	2020-03-22	1.00	Umbria	https://www.gazzettaufficiale.it/showNewsDetail?id=2545&backTo=archivio&anno=2020&provenienza=archivio
Work-from-home order	2020-03-22	1.00	Aosta Valley	https://www.gazzettaufficiale.it/showNewsDetail?id=2545&backTo=archivio&anno=2020&provenienza=archivio

Table 12. Sources for policies implemented across different Italian regions

NPI	Date	Cumulative share	Region	Source

Ban of large gatherings	2020-02-28	1.00	Zurich	https://www.admin.ch/gov/it/pagina-iniziale/documentazione/comunicati-stampa.msg-id-78289.html
Ban of large gatherings	2020-02-28	1.00	Neuchâtel	https://www.admin.ch/gov/it/pagina-iniziale/documentazione/comunicati-stampa.msg-id-78289.html
Ban of large gatherings	2020-02-28	1.00	Valais	https://www.admin.ch/gov/it/pagina-iniziale/documentazione/comunicati-stampa.msg-id-78289.html
Ban of large gatherings	2020-02-28	1.00	Vaud	https://www.admin.ch/gov/it/pagina-iniziale/documentazione/comunicati-stampa.msg-id-78289.html
Ban of large gatherings	2020-02-28	1.00	Ticino	https://www.admin.ch/gov/it/pagina-iniziale/documentazione/comunicati-stampa.msg-id-78289.html
Ban of large gatherings	2020-02-28	1.00	Thurgau	https://www.admin.ch/gov/it/pagina-iniziale/documentazione/comunicati-stampa.msg-id-78289.html
Ban of large gatherings	2020-02-28	1.00	Aargau	https://www.admin.ch/gov/it/pagina-iniziale/documentazione/comunicati-stampa.msg-id-78289.html
Ban of large gatherings	2020-02-28	1.00	Graubünden	https://www.admin.ch/gov/it/pagina-iniziale/documentazione/comunicati-stampa.msg-id-78289.html
Ban of large gatherings	2020-02-28	1.00	St. Gallen	https://www.admin.ch/gov/it/pagina-iniziale/documentazione/comunicati-stampa.msg-id-78289.html
Ban of large gatherings	2020-02-28	1.00	Appenzell Innerrhoden	https://www.admin.ch/gov/it/pagina-iniziale/documentazione/comunicati-stampa.msg-id-78289.html
Ban of large gatherings	2020-02-28	1.00	Appenzell Ausserrhoden	https://www.admin.ch/gov/it/pagina-iniziale/documentazione/comunicati-stampa.msg-id-78289.html
Ban of large gatherings	2020-02-28	1.00	Schaffhausen	https://www.admin.ch/gov/it/pagina-iniziale/documentazione/comunicati-stampa.msg-id-78289.html
Ban of large gatherings	2020-02-28	1.00	Basel-Landschaft	https://www.admin.ch/gov/it/pagina-iniziale/documentazione/comunicati-stampa.msg-id-78289.html

Ban of large gatherings	2020-02-28	1.00	Basel-Stadt	https://www.admin.ch/gov/it/pagina-iniziale/documentazione/comunicati-stampa.msg-id-78289.html
Ban of large gatherings	2020-02-28	1.00	Solothurn	https://www.admin.ch/gov/it/pagina-iniziale/documentazione/comunicati-stampa.msg-id-78289.html
Ban of large gatherings	2020-02-28	1.00	Fribourg	https://www.admin.ch/gov/it/pagina-iniziale/documentazione/comunicati-stampa.msg-id-78289.html
Ban of large gatherings	2020-02-28	1.00	Zug	https://www.admin.ch/gov/it/pagina-iniziale/documentazione/comunicati-stampa.msg-id-78289.html
Ban of large gatherings	2020-02-28	1.00	Glarus	https://www.admin.ch/gov/it/pagina-iniziale/documentazione/comunicati-stampa.msg-id-78289.html
Ban of large gatherings	2020-02-28	1.00	Nidwalden	https://www.admin.ch/gov/it/pagina-iniziale/documentazione/comunicati-stampa.msg-id-78289.html
Ban of large gatherings	2020-02-28	1.00	Obwalden	https://www.admin.ch/gov/it/pagina-iniziale/documentazione/comunicati-stampa.msg-id-78289.html
Ban of large gatherings	2020-02-28	1.00	Schwyz	https://www.admin.ch/gov/it/pagina-iniziale/documentazione/comunicati-stampa.msg-id-78289.html
Ban of large gatherings	2020-02-28	1.00	Uri	https://www.admin.ch/gov/it/pagina-iniziale/documentazione/comunicati-stampa.msg-id-78289.html
Ban of large gatherings	2020-02-28	1.00	Lucerne	https://www.admin.ch/gov/it/pagina-iniziale/documentazione/comunicati-stampa.msg-id-78289.html
Ban of large gatherings	2020-02-28	1.00	Bern	https://www.admin.ch/gov/it/pagina-iniziale/documentazione/comunicati-stampa.msg-id-78289.html
Ban of large gatherings	2020-02-28	1.00	Geneva	https://www.admin.ch/gov/it/pagina-iniziale/documentazione/comunicati-stampa.msg-id-78289.html
Ban of large gatherings	2020-02-28	1.00	Jura	https://www.admin.ch/gov/it/pagina-iniziale/documentazione/comunicati-stampa.msg-id-78289.html

NPI	Date	Cumulative share	Region	Source
Ban of small gatherings	2020-03-16	0.02	Neuchâtel	https://www.ne.ch/medias/Pages/20200315-mesures-urgence-lutte-covid19-canton-de-neuchatel.aspx
Ban of small gatherings	2020-03-18	0.03	Jura	https://www.jura.ch/CHA/SIC/Centre-medias/Communiques-2020/COVID-19-etat-de-necessite-decrete-et-interdiction-des-rassemblements-de-plus-de-5-personnes.html
Ban of small gatherings	2020-03-20	1.00	Valais	https://www.admin.ch/gov/it/pagina-iniziale/documentazione/comunicati-stampa/comunicati-stampa-consiglio-federale.msg-id-78513.html
Ban of small gatherings	2020-03-20	1.00	Vaud	https://www.admin.ch/gov/it/pagina-iniziale/documentazione/comunicati-stampa/comunicati-stampa-consiglio-federale.msg-id-78513.html
Ban of small gatherings	2020-03-20	1.00	Ticino	https://www.admin.ch/gov/it/pagina-iniziale/documentazione/comunicati-stampa/comunicati-stampa-consiglio-federale.msg-id-78513.html
Ban of small gatherings	2020-03-20	1.00	Thurgau	https://www.admin.ch/gov/it/pagina-iniziale/documentazione/comunicati-stampa/comunicati-stampa-consiglio-federale.msg-id-78513.html
Ban of small gatherings	2020-03-20	1.00	Aargau	https://www.admin.ch/gov/it/pagina-iniziale/documentazione/comunicati-stampa/comunicati-stampa-consiglio-federale.msg-id-78513.html
Ban of small gatherings	2020-03-20	1.00	Graubünden	https://www.admin.ch/gov/it/pagina-iniziale/documentazione/comunicati-stampa/comunicati-stampa-consiglio-federale.msg-id-78513.html
Ban of small gatherings	2020-03-20	1.00	St. Gallen	https://www.admin.ch/gov/it/pagina-iniziale/documentazione/comunicati-stampa/comunicati-stampa-consiglio-federale.msg-id-78513.html
Ban of small gatherings	2020-03-20	1.00	Appenzell Innerrhoden	https://www.admin.ch/gov/it/pagina-iniziale/documentazione/comunicati-stampa/comunicati-stampa-consiglio-federale.msg-id-78513.html
Ban of small gatherings	2020-03-20	1.00	Appenzell Ausserrhoden	https://www.admin.ch/gov/it/pagina-iniziale/documentazione/comunicati-stampa/comunicati-stampa-consiglio-federale.msg-id-78513.html

Ban of small gatherings	2020-03-20	1.00	Schaffhausen	https://www.admin.ch/gov/it/pagina-iniziale/documentazione/comunicati-stampa/comunicati-stampa-consiglio-federale.msg-id-78513.html
Ban of small gatherings	2020-03-20	1.00	Zurich	https://www.admin.ch/gov/it/pagina-iniziale/documentazione/comunicati-stampa/comunicati-stampa-consiglio-federale.msg-id-78513.html
Ban of small gatherings	2020-03-20	1.00	Basel-Stadt	https://www.admin.ch/gov/it/pagina-iniziale/documentazione/comunicati-stampa/comunicati-stampa-consiglio-federale.msg-id-78513.html
Ban of small gatherings	2020-03-20	1.00	Solothurn	https://www.admin.ch/gov/it/pagina-iniziale/documentazione/comunicati-stampa/comunicati-stampa-consiglio-federale.msg-id-78513.html
Ban of small gatherings	2020-03-20	1.00	Fribourg	https://www.admin.ch/gov/it/pagina-iniziale/documentazione/comunicati-stampa/comunicati-stampa-consiglio-federale.msg-id-78513.html
Ban of small gatherings	2020-03-20	1.00	Zug	https://www.admin.ch/gov/it/pagina-iniziale/documentazione/comunicati-stampa/comunicati-stampa-consiglio-federale.msg-id-78513.html
Ban of small gatherings	2020-03-20	1.00	Glarus	https://www.admin.ch/gov/it/pagina-iniziale/documentazione/comunicati-stampa/comunicati-stampa-consiglio-federale.msg-id-78513.html
Ban of small gatherings	2020-03-20	1.00	Nidwalden	https://www.admin.ch/gov/it/pagina-iniziale/documentazione/comunicati-stampa/comunicati-stampa-consiglio-federale.msg-id-78513.html
Ban of small gatherings	2020-03-20	1.00	Obwalden	https://www.admin.ch/gov/it/pagina-iniziale/documentazione/comunicati-stampa/comunicati-stampa-consiglio-federale.msg-id-78513.html
Ban of small gatherings	2020-03-20	1.00	Schwyz	https://www.admin.ch/gov/it/pagina-iniziale/documentazione/comunicati-stampa/comunicati-stampa-consiglio-federale.msg-id-78513.html
Ban of small gatherings	2020-03-20	1.00	Uri	https://www.admin.ch/gov/it/pagina-iniziale/documentazione/comunicati-stampa/comunicati-stampa-consiglio-federale.msg-id-78513.html
Ban of small gatherings	2020-03-20	1.00	Lucerne	https://www.admin.ch/gov/it/pagina-iniziale/documentazione/comunicati-stampa/comunicati-stampa-consiglio-federale.msg-id-78513.html
Ban of small gatherings	2020-03-20	1.00	Bern	https://www.admin.ch/gov/it/pagina-iniziale/documentazione/comunicati-stampa/comunicati-stampa-consiglio-federale.msg-id-78513.html

Ban of small gatherings	2020-03-20	1.00	Geneva	https://www.admin.ch/gov/it/pagina-iniziale/documentazione/comunicati-stampa/comunicati-stampa-consiglio-federale.msg-id-78513.html
Ban of small gatherings	2020-03-20	1.00	Basel-Landschaft	https://www.admin.ch/gov/it/pagina-iniziale/documentazione/comunicati-stampa/comunicati-stampa-consiglio-federale.msg-id-78513.html

**NPI**	**Date**	**Cumulative share**	**Region**	**Source**
School closure	2020-03-16	1.00	Zurich	https://www.admin.ch/gov/it/pagina-iniziale/documentazione/comunicati-stampa/comunicati-stampa-consiglio-federale.html?dyn_startDate=01.01.2015
School closure	2020-03-16	1.00	Neuchâtel	https://www.admin.ch/gov/it/pagina-iniziale/documentazione/comunicati-stampa/comunicati-stampa-consiglio-federale.html?dyn_startDate=01.01.2015
School closure	2020-03-16	1.00	Valais	https://www.admin.ch/gov/it/pagina-iniziale/documentazione/comunicati-stampa/comunicati-stampa-consiglio-federale.html?dyn_startDate=01.01.2015
School closure	2020-03-16	1.00	Vaud	https://www.admin.ch/gov/it/pagina-iniziale/documentazione/comunicati-stampa/comunicati-stampa-consiglio-federale.html?dyn_startDate=01.01.2015
School closure	2020-03-16	1.00	Ticino	https://www.admin.ch/gov/it/pagina-iniziale/documentazione/comunicati-stampa/comunicati-stampa-consiglio-federale.html?dyn_startDate=01.01.2015
School closure	2020-03-16	1.00	Thurgau	https://www.admin.ch/gov/it/pagina-iniziale/documentazione/comunicati-stampa/comunicati-stampa-consiglio-federale.html?dyn_startDate=01.01.2015

School closure	2020-03-16	1.00	Aargau	https://www.admin.ch/gov/it/pagina-iniziale/documentazione/comunicati-stampa/comunicati-stampa-consiglio-federale.html?dyn_startDate=01.01.2015
School closure	2020-03-16	1.00	Graubünden	https://www.admin.ch/gov/it/pagina-iniziale/documentazione/comunicati-stampa/comunicati-stampa-consiglio-federale.html?dyn_startDate=01.01.2015
School closure	2020-03-16	1.00	St. Gallen	https://www.admin.ch/gov/it/pagina-iniziale/documentazione/comunicati-stampa/comunicati-stampa-consiglio-federale.html?dyn_startDate=01.01.2015
School closure	2020-03-16	1.00	Appenzell Innerrhoden	https://www.admin.ch/gov/it/pagina-iniziale/documentazione/comunicati-stampa/comunicati-stampa-consiglio-federale.html?dyn_startDate=01.01.2015
School closure	2020-03-16	1.00	Appenzell Ausserrhoden	https://www.admin.ch/gov/it/pagina-iniziale/documentazione/comunicati-stampa/comunicati-stampa-consiglio-federale.html?dyn_startDate=01.01.2015
School closure	2020-03-16	1.00	Schaffhausen	https://www.admin.ch/gov/it/pagina-iniziale/documentazione/comunicati-stampa/comunicati-stampa-consiglio-federale.html?dyn_startDate=01.01.2015
School closure	2020-03-16	1.00	Basel-Landschaft	https://www.admin.ch/gov/it/pagina-iniziale/documentazione/comunicati-stampa/comunicati-stampa-consiglio-federale.html?dyn_startDate=01.01.2015
School closure	2020-03-16	1.00	Basel-Stadt	https://www.admin.ch/gov/it/pagina-iniziale/documentazione/comunicati-stampa/comunicati-stampa-consiglio-federale.html?dyn_startDate=01.01.2015

School closure	2020-03-16	1.00	Solothurn	https://www.admin.ch/gov/it/pagina-iniziale/documentazione/comunicati-stampa/comunicati-stampa-consiglio-federale.html?dyn_startDate=01.01.2015
School closure	2020-03-16	1.00	Fribourg	https://www.admin.ch/gov/it/pagina-iniziale/documentazione/comunicati-stampa/comunicati-stampa-consiglio-federale.html?dyn_startDate=01.01.2015
School closure	2020-03-16	1.00	Zug	https://www.admin.ch/gov/it/pagina-iniziale/documentazione/comunicati-stampa/comunicati-stampa-consiglio-federale.html?dyn_startDate=01.01.2015
School closure	2020-03-16	1.00	Glarus	https://www.admin.ch/gov/it/pagina-iniziale/documentazione/comunicati-stampa/comunicati-stampa-consiglio-federale.html?dyn_startDate=01.01.2015
School closure	2020-03-16	1.00	Nidwalden	https://www.admin.ch/gov/it/pagina-iniziale/documentazione/comunicati-stampa/comunicati-stampa-consiglio-federale.html?dyn_startDate=01.01.2015
School closure	2020-03-16	1.00	Obwalden	https://www.admin.ch/gov/it/pagina-iniziale/documentazione/comunicati-stampa/comunicati-stampa-consiglio-federale.html?dyn_startDate=01.01.2015
School closure	2020-03-16	1.00	Schwyz	https://www.admin.ch/gov/it/pagina-iniziale/documentazione/comunicati-stampa/comunicati-stampa-consiglio-federale.html?dyn_startDate=01.01.2015
School closure	2020-03-16	1.00	Uri	https://www.admin.ch/gov/it/pagina-iniziale/documentazione/comunicati-stampa/comunicati-stampa-consiglio-federale.html?dyn_startDate=01.01.2015

School closure	2020-03-16	1.00	Lucerne	https://www.admin.ch/gov/it/pagina-iniziale/documentazione/comunicati-stampa/comunicati-stampa-consiglio-federale.html?dyn_startDate=01.01.2015
School closure	2020-03-16	1.00	Bern	https://www.admin.ch/gov/it/pagina-iniziale/documentazione/comunicati-stampa/comunicati-stampa-consiglio-federale.html?dyn_startDate=01.01.2015
School closure	2020-03-16	1.00	Geneva	https://www.admin.ch/gov/it/pagina-iniziale/documentazione/comunicati-stampa/comunicati-stampa-consiglio-federale.html?dyn_startDate=01.01.2015
School closure	2020-03-16	1.00	Jura	https://www.admin.ch/gov/it/pagina-iniziale/documentazione/comunicati-stampa/comunicati-stampa-consiglio-federale.html?dyn_startDate=01.01.2015

**NPI**	**Date**	**Cumulative share**	**Region**	**Source**
Venue closure	2020-03-14	0.04	Ticino	https://www4.ti.ch/dss/dsp/covid19/home/
Venue closure	2020-03-16	0.09	Jura	https://www.jura.ch/CHA/SIC/Centre-medias/Communiques-2020/COVID-19-le-canton-du-Jura-prend-des-mesures-supplementaires-pour-proteger-la-population-et-enrayer-la-propagation-du-coronaviru.html
Venue closure	2020-03-16	0.09	Neuchâtel	https://www.ne.ch/medias/Pages/20200315-mesures-urgence-lutte-covid19-canton-de-neuchatel.aspx
Venue closure	2020-03-16	0.09	Graubünden	https://www.kantonsamtsblatt.gr.ch/it/efuc/00.045.026/publikation/
Venue closure	2020-03-17	1.00	Valais	https://www.admin.ch/gov/it/pagina-iniziale/documentazione/comunicati-stampa/comunicati-stampa-consiglio-federale.html?dyn_startDate=01.01.2015

Venue closure	2020-03-17	1.00	Vaud	https://www.admin.ch/gov/it/pagina-iniziale/documentazione/comunicati-stampa/comunicati-stampa-consiglio-federale.html?dyn_startDate=01.01.2015
Venue closure	2020-03-17	1.00	Thurgau	https://www.admin.ch/gov/it/pagina-iniziale/documentazione/comunicati-stampa/comunicati-stampa-consiglio-federale.html?dyn_startDate=01.01.2015
Venue closure	2020-03-17	1.00	Aargau	https://www.admin.ch/gov/it/pagina-iniziale/documentazione/comunicati-stampa/comunicati-stampa-consiglio-federale.html?dyn_startDate=01.01.2015
Venue closure	2020-03-17	1.00	St. Gallen	https://www.admin.ch/gov/it/pagina-iniziale/documentazione/comunicati-stampa/comunicati-stampa-consiglio-federale.html?dyn_startDate=01.01.2015
Venue closure	2020-03-17	1.00	Appenzell Innerrhoden	https://www.admin.ch/gov/it/pagina-iniziale/documentazione/comunicati-stampa/comunicati-stampa-consiglio-federale.html?dyn_startDate=01.01.2015
Venue closure	2020-03-17	1.00	Appenzell Ausserrhoden	https://www.admin.ch/gov/it/pagina-iniziale/documentazione/comunicati-stampa/comunicati-stampa-consiglio-federale.html?dyn_startDate=01.01.2015
Venue closure	2020-03-17	1.00	Schaffhausen	https://www.admin.ch/gov/it/pagina-iniziale/documentazione/comunicati-stampa/comunicati-stampa-consiglio-federale.html?dyn_startDate=01.01.2015
Venue closure	2020-03-17	1.00	Zurich	https://www.admin.ch/gov/it/pagina-iniziale/documentazione/comunicati-stampa/comunicati-stampa-consiglio-federale.html?dyn_startDate=01.01.2015

Venue closure	2020-03-17	1.00	Basel-Stadt	https://www.admin.ch/gov/it/pagina-iniziale/documentazione/comunicati-stampa/comunicati-stampa-consiglio-federale.html?dyn_startDate=01.01.2015
Venue closure	2020-03-17	1.00	Solothurn	https://www.admin.ch/gov/it/pagina-iniziale/documentazione/comunicati-stampa/comunicati-stampa-consiglio-federale.html?dyn_startDate=01.01.2015
Venue closure	2020-03-17	1.00	Fribourg	https://www.admin.ch/gov/it/pagina-iniziale/documentazione/comunicati-stampa/comunicati-stampa-consiglio-federale.html?dyn_startDate=01.01.2015
Venue closure	2020-03-17	1.00	Zug	https://www.admin.ch/gov/it/pagina-iniziale/documentazione/comunicati-stampa/comunicati-stampa-consiglio-federale.html?dyn_startDate=01.01.2015
Venue closure	2020-03-17	1.00	Glarus	https://www.admin.ch/gov/it/pagina-iniziale/documentazione/comunicati-stampa/comunicati-stampa-consiglio-federale.html?dyn_startDate=01.01.2015
Venue closure	2020-03-17	1.00	Nidwalden	https://www.admin.ch/gov/it/pagina-iniziale/documentazione/comunicati-stampa/comunicati-stampa-consiglio-federale.html?dyn_startDate=01.01.2015
Venue closure	2020-03-17	1.00	Obwalden	https://www.admin.ch/gov/it/pagina-iniziale/documentazione/comunicati-stampa/comunicati-stampa-consiglio-federale.html?dyn_startDate=01.01.2015
Venue closure	2020-03-17	1.00	Schwyz	https://www.admin.ch/gov/it/pagina-iniziale/documentazione/comunicati-stampa/comunicati-stampa-consiglio-federale.html?dyn_startDate=01.01.2015

Venue closure	2020-03-17	1.00	Uri	https://www.admin.ch/gov/it/pagina-iniziale/documentazione/comunicati-stampa/comunicati-stampa-consiglio-federale.html?dyn_startDate=01.01.2015
Venue closure	2020-03-17	1.00	Lucerne	https://www.admin.ch/gov/it/pagina-iniziale/documentazione/comunicati-stampa/comunicati-stampa-consiglio-federale.html?dyn_startDate=01.01.2015
Venue closure	2020-03-17	1.00	Bern	https://www.admin.ch/gov/it/pagina-iniziale/documentazione/comunicati-stampa/comunicati-stampa-consiglio-federale.html?dyn_startDate=01.01.2015
Venue closure	2020-03-17	1.00	Geneva	https://www.admin.ch/gov/it/pagina-iniziale/documentazione/comunicati-stampa/comunicati-stampa-consiglio-federale.html?dyn_startDate=01.01.2015
Venue closure	2020-03-17	1.00	Basel-Landschaft	https://www.admin.ch/gov/it/pagina-iniziale/documentazione/comunicati-stampa/comunicati-stampa-consiglio-federale.html?dyn_startDate=01.01.2015
**NPI**	**Date**	**Cumulative share**	**Region**	**Source**

**NPI**	**Date**	**Cumulative share**	**Region**	**Source**
Work-from-home order	2020-03-14	0.04	Ticino	https://www4.ti.ch/dss/dsp/covid19/home/

Table 13. Sources for policies implemented across different Swiss regions

References

1. Cori A, Ferguson NM, Fraser C, Cauchemez S. A New Framework and Software to Estimate Time-Varying Reproduction Numbers During Epidemics. American Journal of Epidemiology. 2013 4;178(9):1505–1512. Available from: https://academic.oup.com/aje/article-lookup/doi/10.1093/aje/kwt133.

2. Fraser C. Estimating Individual and Household Reproduction Numbers in an Emerging Epidemic. PLoS ONE. 2007 4;2(8):e758. Available from: https://dx.plos.org/10.1371/journal.pone.0000758.

3. McAloon C, Collins A, Hunt K, Barber A, Byrne AW, Butler F, et al. Incubation period of COVID-19: a rapid systematic review and meta-analysis of observational research. BMJ Open. 2020 4;10(8):e039652. Available from: https://bmjopen.bmj.com/lookup/doi/10.1136/bmjopen-2020-039652.

4. Cereda D, Tirani M, Rovida F, Demicheli V, Ajelli M, Poletti P, et al. The early phase of the COVID-19 outbreak in Lombardy, Italy. arXiv. 2020 4;Available from: http://arxiv.org/abs/2003.09320.

5. Akhmetzhanov AR, Mizumoto K, Jung Sm, Linton NM, Omori R, Nishiura H. Epidemiological characteristics of novel coronavirus infection: A statistical analysis of publicly available case data. medRxiv. 2020;Available from: https://www.medrxiv.org/content/medrxiv/early/2020/04/30/2020.04.24.20077800.full.pdf.

6. Günther F, Bender A, Katz K, Küchenhoff H, Höhle M. Nowcasting the COVID-19 pandemic in Bavaria. Biometrical Journal. 2020 4;p. bimj.202000112. Available from: https://onlinelibrary.wiley.com/doi/10.1002/bimj.202000112.

7. Leung K, Wu JT, Liu D, Leung GM. First-wave COVID-19 transmissibility and severity in China outside Hubei after control measures, and second-wave scenario planning: a modelling impact assessment. The Lancet. 2020 4;395(10233):1382–1393. Available from: https://linkinghub.elsevier.com/retrieve/pii/S0140673620307467.

8. Tariq A, Lee Y, Roosa K, Blumberg S, Yan P, Ma S, et al. Real-time monitoring the transmission potential of COVID-19 in Singapore, March 2020. BMC Medicine. 2020 4;18(1):166. Available

from: https://bmcmedicine.biomedcentral.com/articles/10.1186/s12916-020-01615-9.

9. Flaxman S, Mishra S, Gandy A, Unwin HJT, Mellan TA, Coupland H, et al. Estimating the effects of non-pharmaceutical interventions on COVID-19 in Europe. Nature. 2020 4;584(7820):257–261. Available from: http://www.nature.com/articles/s41586-020-2405-7.

10. Lehtinen S, Ashcroft P, Bonhoeffer S. On the relationship between serial interval, infectiousness profile and generation time. medRxiv. 2020 4;Available from: https://doi.org/10.1101/2020.09.18.20197210.

11. Ferretti L, Wymant C, Kendall M, Zhao L, Nurtay A, Abeler-Dörner L, et al. Quantifying SARS-CoV-2 transmission suggests epidemic control with digital contact tracing. Science. 2020 4;368(6491):eabb6936. Available from: https://www.sciencemag.org/lookup/doi/10.1126/science.abb6936.

12. Ganyani T, Kremer C, Chen D, Torneri A, Faes C, Wallinga J, et al. Estimating the generation interval for coronavirus disease (COVID-19) based on symptom onset data, March 2020. Euro surveillance : bulletin Europeen sur les maladies transmissibles = European communicable disease bulletin. 2020 4;25(17):2000257. Available from: https://pubmed.ncbi.nlm.nih.gov/32372755https://www.ncbi.nlm.nih.gov/pmc/articles/PMC7201952/.

13. Ferretti L, Ledda A, Wymant C, Zhao L, Ledda V, Abeler L, et al. The timing of COVID-19 transmission. medRxiv. 2020 4;Available from: https://www.medrxiv.org/content/10.1101/2020.09.04.20188516v2.

14. Liu Y, Gayle AA, Wilder-Smith A, Rocklöv J. The reproductive number of COVID-19 is higher compared to SARS coronavirus. Journal of Travel Medicine. 2020 4;27(2). Available from: https://academic.oup.com/jtm/article/doi/10.1093/jtm/taaa021/5735319.

15. Stan Development Team. Prior Choice Recommendations; 2020. Available from: https://github.com/stan-dev/stan/wiki/Prior-Choice-Recommendations.

16. Carpenter B, Gelman A, Hoffman MD, Lee D, Goodrich B, Betancourt M, et al. Stan : A Probabilistic Programming Language. Journal of Statistical Software. 2017;76(1). Available from: http://www.jstatsoft.org/v76/i01/.

17. Brauner JM, Mindermann S, Sharma M, Johnston D, Salvatier J, Gavenčiak T, et al. Inferring the effectiveness of government interventions against COVID-19. Science. 2020 4;p. eabd9338. Available from: https://www.sciencemag.org/lookup/doi/10.1126/science.abd9338.

18. Stan Development Team. Stan modeling language users guide (Version 2.21); 2019. Available from: https://mc-stan.org/docs/2_21/reference-manual/effective-sample-size-section.html.

19. Gelman A, Rubin DB, others. Inference from iterative simulation using multiple sequences. Statistical science. 1992;7(4):457–472.

20. Gelman A, Carlin JB, Stern HS, Dunson DB, Vehtari A, Rubin DB. Bayesian Data Analysis. Chapman & Hall; 2013.

21. Vehtari A, Gelman A, Gabry J. Practical Bayesian model evaluation using leave-one-out cross-validation and WAIC. Statistics and Computing. 2017;27(5):1413–1432.

22. Hale T, Webster S, Petherick A, Phillips T, Kira B. Oxford COVID-19 Government Response Tracker. Blavatnik School of Government. 2020;Available from: https://www.bsg.ox.ac.uk/research/research-projects/coronavirus-government-response-tracker.
