# Supplementary material for: Estimating the effects of non-pharmaceutical interventions on the number of new infections with COVID-19 during the first epidemic wave
Source: PLoS One. 2021 Jun 2;16(6):e0252827. doi: 10.1371/journal.pone.0252827 (PMC8171941; doi:10.1371/journal.pone.0252827)
Supplement: Attachment — Submitted filename: review Banholzer.pdf [file pone.0252827.s002.pdf]

This is a great paper. Here are some of the things that are good about it:

- Highly important, and still somewhat understudied, topic.
- Extremely well written, very well structured and easy to follow, very reader-friendly (also to non-experts), good flow.
- Solid modelling, with well justified parameter assumptions.
- Has a data validation process (unlike many other papers in this field)
- Key assumptions are made very transparent.
- The authors put a lot of effort into making this paper useful for the community, and making it easy for others to build on and interact with their research. Examples:
    - The thorough comparison with Brauner et al. This type of comparison is not usually done in related papers but is obviously crucial for the health of the field.
    - Code and data are easily available. The code is, for academic standards, exceptionally clean and well-documented.
- The authors take into account the share of the population affected by an NPI. This is not usually done for cross-country analyses, as most other papers rely on the OXGCRT dataset, which only has national data. (This also means that the "effective dataset size" of this study is larger than 20 countries)

It's always easy to come up with many ways to improve any modelling study. So we could now spend months in the revision process, implementing these. But I'm pretty sure that the policy-relevant conclusions would barely change. This paper is clearly already one of the better papers on this topics, and rather than requesting changes to satisfy my maybe idiosyncratic tastes, I want this to be published soon to raise the sanity waterline of the field.

Basically, I'm happy to recommend acceptance of the manuscript as is. I group my suggestions into three groups:

- Priority 1: These are easy to address and they do improve the paper, so I recommend you implement them. **If the "priority 1" suggestions are addressed, then I'll definitely recommend acceptance. The suggestions of priority 2 and 3 are strictly optional.**
- Priority 2: These points are a bit more work to address. Only address them if you think my point is reasonable and worth the effort. Please don't make any changes if you're not convinced of it.
- Priority 3: These points are to some degree a matter of taste. Again, only address them if you think my point is reasonable and worth the effort. Please don't make any changes if you're not convinced of it.

Priority 1:

- NPI definitions and names - Table 1:
    - For interpreting this study, it crucial that readers can understand the NPIs studied. I suggest that you describe NPI definitions in more detail. For example, the "event ban" definition refers to cancellation of gatherings of 50 people or more, but the supplement reveals that the NPI was recorded even is only events of >5000 were banned. Similarly, the definition for "venue closure" is quite vague. Was this NPI recorded if any of these things were closed? There are many recreational venues, so did the closure of night clubs (but nothing else) suffice to trigger this NPI? What was the limit on gathering size for a "gathering ban" to be recorded?
    - I am aware that it is hard to define these NPIs so that they carve reality well. Any definition will still require many judgement calls from the data collectors. Other papers get around this by simply using public datasets (e.g. OXCGRT), but, of course, these datasets have the exact same problems. The authors should be commended for collecting their own data and putting effort into validating it. I simply recommend describing the NPIs a bit more, even if the working definitions for the collection were somewhat vague. E.g. what did an NPI of this type typically look like (e.g. was the limit on events typically at 5000 people or at 50)?
    - In a similar vein, you could probably make the manuscript more reader-friendly by coming up with better, more intuitive NPI names. "E.g. "ban of large gatherings" instead of "events ban". Or "closure of leisures time venues and/or gastronomy" instead of venue closure. Just spitballing here, and I don't advocate for these particular examples. But maybe you can come up with names that more intuitively hint at the definitions. Currently, one first has to read and understand Table 1 before one gets an idea what is meant by some of the NPIs.
- Communicating the hierarchy of NPIs better:
    - Taking the NPI definitions in Table 1 literally, then a "work ban" implies a "venue closure". "Closure of non-essential business activities" implies "Closure of venues for recreational activities and/or shops, bars, and restaurants". So the effect you estimate for the "work ban" is not the effect of "Closure of non-essential business activities", but rather the additional effect of closing the remaining non-essential business activities, when the effect of closing "venues" has already been accounted for. This should be explained somewhere. The same probably goes for "gathering ban" and "event ban".
- Clarify Figures 4d:
    - You write: "Regarding the magnitude of the effects, we could be at least 91% sure that three NPIs simultaneously lead to a reduction in the number of new infections of more than 10%." But the way I understood Figure 4d, it shows that you can be 91% sure that there are 3 NPIs which *each* have an effect of >10%. Please clarify.

Priority 2 (These points are a bit more work to address. Only address them if you think my point is reasonable and worth the effort.):

- Work ban:
    - I'm confused about this NPI. Does this NPI actually refer to closure of all non-essential businesses activities (including the closure of factories, offices, and so on)? If so:
        - 1. This should be explained better in the definition. From the current definition, the "work ban" NPI looks very similar to Brauner's "most businesses closed"
        - 2. I don't think any country actually did close factories, right?
    - In an alternative interpretation, this NPI refers to face-to-face, customer-facing businesses only, as in Brauner et al. If so:
        - 1. The name "work ban" is very misleading. Most work does not happen in face-to-face, customer facing businesses, but in offices, factories, and so on.
    - I tried to figure out what was meant by looking through a few sources. The "work ban" is recorded for very few countries, implying that probably the former definition is meant. However, I'm still not sure, because:
        - The source for France doesn't work, as it links to the current COVID page
        - For Ireland, the source doesn't contain info on work ban, as far as I can see. But it says that it was "Last updated on 16 September 2020"
        - For Luxembourg, it looks like only face-to-face businesses were closed.
    - If the authors also think that the definition of work ban might be a bit shaky, I suggest to consider removing this NPI. But it's also well possible that there was a consistent definition during the data collection. In either case, I suggest communicating this a bit more clearly.
- Most readers won't read the supplement and thereby don't get a feeling for how robust the results are. Maybe you can include a summary figure that aggregates the results from all sensitivity analyses in the main text.

Priority 3 (These points are even more idiosyncratic/a matter of taste. Again, only address them if you think my point is reasonable and worth the effort. Please don't make any changes if you're not convinced of it.)

- Table 2b: If your "event ban" refers to events with >50 attendants (as the definition says), then this seems more similar to Brauner's Gatherings < 100. But it doesn't matter much, as Gatherings <1000 and Gatherings<100 are highly collinear in Brauner et al.
- I don't think that it's clear that NPI effects are delayed in time. E.g., for schools closures, they are clearly not. Also, I expect this time delayed effect to just trade-off with the infection-to-case confirmation delay, which you infer from the data. However, this is mostly a matter of taste, and the sensitivity analysis shows that it doesn't matter.
- It would be mildly interesting to see Brauner et al.'s model with your data. Especially, if you want to corroborate the following claim (Discussion) some more: "The check indicated that the choice of countries and definition of NPIs has a larger influence on the

estimated effects than the detailed choices in modeling". Again, absolutely not necessary, just mildly interesting.

- Section 4.2 is probably not interesting to most people. If it was me, I'd maybe shorten it to 3 sentences that summarise the key difference, and put the full section into the appendix. The paper is becoming a bit long-winded towards the end, anyway.
- Running the model with death data seems like a relatively cheap and robust way to analyse the robustness of the results, and rule out (to some degree) a large influence of changes in the ascertainment rate.
- I find the NPI effect prior a bit un-intuitive. I think it's more natural to assume that small effects are more likely a priori than large effects.

Some minor points about the presentation:

- Page 3, line 52: I think you meant to say "ambiguous", and not "not ambiguous"
- Figure 1: "Number of new cases per 100,000 (rolling 7-day mean) until NPIs were implemented across countries." - I think it would be easier to understand if you wrote "when NPIs were first implemented"
- page 17, line 175: "The authors used similar priors for the distribution of the time from infection to reporting and for the generation time distribution, but estimated the latter explicitly as part of fitting the model." - Brauner et al estimate delay distribution and the generative interval distribution from data. I think what you mean to say is that in contrast to your paper, Brauner et al also estimates the GI from data?
- Figure 5: In the legend, observed N should not have a shaded frame around it, as this implies the credible intervals.
- Table 2: "Note that, in these analyses, we report cumulative effects for gathering bans and businesses closed." - I would add "as in Brauner et al.", to make it clear why you are doing this.

Best,
Jan Brauner
