# Supplementary material for: Estimating the effects of non-pharmaceutical interventions on the number of new infections with COVID-19 during the first epidemic wave
Source: PLoS One. 2021 Jun 2;16(6):e0252827. doi: 10.1371/journal.pone.0252827 (PMC8171941; doi:10.1371/journal.pone.0252827)
Supplement: Attachment — Submitted filename: Response to the Reviewers.pdf [file pone.0252827.s003.pdf]

Dear Dr Lolli, dear reviewers,

Thank you for the excellent feedback and suggestions we received for our previous submission to *PLOS ONE* (manuscript ID PONE-D-21-01565). It was encouraging to see that the review team found merit in our paper.

We have carefully reviewed and considered all your comments. Below you will find a summary of the most important changes. This is followed by a point-by-point response to you and the review team on how we have improved our manuscript based on your comments. We are convinced that the manuscript benefited greatly from your comments. We hope you agree.

In our paper, we studied the effects of non-pharmaceutical interventions (NPIs) during the first wave of the COVID-19 epidemic. This choice was intentional. As of now, there are mixed findings to what extent different NPIs were effective, and hence researchers are confronted with uncertainty. The reason is that most studies were looking at the overall effectiveness of NPIs, while only few studies attempted to estimate the effects of individual NPIs, thereby arriving at different conclusions. In line with this, several studies have questioned specific assumptions of the underlying models as well as the robustness of the findings [Soltesz 2020, Besançon 2021], which calls for more research in this regard, especially by taking into consideration further parameters. Our paper makes a contribution to this by applying a model which differs partially from previously used ones and, in particular, allows to account for the affected size of subpopulations.

Revision summary: Most important changes

1. <u>Originality of our work:</u> Reviewer #2 was mainly concerned that our study is no significant advancement over the study by Brauner et al. [Brauner 2020]. We are thankful for the opportunity to highlight several important differences. First, our model differs in several aspects from that used by Brauner et al., The most innovative aspect is the following: our model takes into account subnational variation in the implementation of NPIs, thereby basically 'weighting' the effect of an NPI by the share of the national population that is affected by it at any point in time. Furthermore, our sample of countries overlaps only partly with the one by Brauner et al. Therefore, we estimate the effects of NPIs based on a different study population. Taken together, we firmly believe that our work contributes significantly to the field by providing novel model extensions and analyzing a different study population. The comparison with the study by Brauner et al. is to inform about the influence of model and data choice on the estimated effectiveness of NPIs. Such comparison is extremely valuable in order to check the reproducibility of findings, which potentially have a high impact on public policy.

2. Name and definitions of NPIs: Reviewer #1 has made great comments on how to clarify the names and definitions of the NPIs in our data. We followed the suggestions closely and renamed our NPIs accordingly (see the revised Table 1 in the manuscript and the revised S1 Appendix 8.1). We are convinced that these changes improved the readability and interpretability of our work.
3. Demonstrating identifiability of the NPI effects: Reviewer #2 was concerned about the identifiability of our model. To alleviate such concerns, we followed good practice in Bayesian modeling [Gelman 2020] and conducted a simulation-based study, which confirms that our model can recover the true effects of NPIs (see the revised Section 2.3 and the new S1 Appendix 3). The results from this simulation-based study show that identifiability is ensured, thereby providing further assurance that the main findings from our study are reproducible and robust. This is important, given the profound impact of the research topic and previous uncertainties regarding estimations.

Response to Academic Editor

Dear Dr Lolli,

Thank you very much for taking the time to review our manuscript, finding reviewers, and providing us with the opportunity to revise our manuscript and address the issues and comments raised by the reviewers.

E.1: *"First of all I apologize for the delay in peer-review process, but even if COVID-19 studies are prioritized, the resources are still limited in terms of finding available reviewers due to the large number of manuscript submitted on this topic. Regarding the manuscript, please note that the two reviewers came to diverging conclusions (minor revisions vs. reject). The paper originality is questioned by Reviewer #2, i.e. no significant advancement with respect to Brauner et al. study. Moreover, also some methodological aspects should be addressed. However, as recognized by Reviewer #2, the study is interesting and for this reason I reached the "Major Revision" decision. The authors should address and respond point-by-point to the reviewer issues/comments when drafting the new version."*

Response to E.1: We understand that it is difficult to find good reviewers and thus we very much appreciate the time and effort you spend in organizing the peer-review process. We are pleased with your decision for a major revision and we think it led to great improvements of our work.

***Clear exposition of contribution*:** We would like to emphasize that we disagree with the assertion by reviewer #2 that there is no significant advancement by our study over the study by Brauner et al. [Brauner 2020]. Dr Brauner himself is a reviewer of this study and has provided a very positive summary of our work, recognizing its quality and contributions. However, we agree that the differences could be elaborated more clearly. In our response to reviewer #2 (see Response to R2.1), we now summarize our important contributions, namely, novel model extensions and a different study population. Because our model is in part different to that of Brauner et al., we also perform a comparison with their work.

***Significance*:** We feel that the decision by reviewer #2 was mostly based on 'perceived significance'. Reviewer #2 argued that the significance of our work is low as we study the first wave (rather than a second or third wave). We gently disagree with reviewer #2 in this regard. To this date, there is great uncertainty with regard to the estimated effects of NPIs, even during the first wave of the pandemic (e.g., see the works in [Brauner 2020, Soltesz 2020, Besançon 2021]). We further oppose reviewer #2 in the fact that our manuscript is evaluated based on 'perceived significance'. The discussion regarding the significance of our study may be a subjective one. Thus, we would like to note that we specifically submitted to PLOS ONE because its mission is to *"evaluate research on*

scientific validity, strong methodology, and high ethical standards – not perceived significance" (see https://journals.plos.org/plosone/static/publish). We hope that our revision can move the discussion towards the study's validity, methodology, and findings.

We followed all suggestions by the reviewers carefully and made extensive changes to our work. We are confident that these add to our study's validity, methodology, and findings. We hope that this is positively received by the reviewers.

Response to Reviewer #1

Dear Dr Brauner,

We are indebted for your excellent and specific comments that have helped us improve the manuscript.

R1.1: *"This is a great paper. Here are some of the things that are good about it:*

- *Highly important, and still somewhat understudied, topic.*
- *Extremely well written, very well structured and easy to follow, very reader-friendly (also to non-experts), good flow.*
- *Solid modelling, with well justified parameter assumptions.*
- *Has a data validation process (unlike many other papers in this field)*
- *Key assumptions are made very transparent.*
- *The authors put a lot of effort into making this paper useful for the community, and making it easy for others to build on and interact with their research. Examples:*
  - *The thorough comparison with Brauner et al. This type of comparison is not usually done in related papers but is obviously crucial for the health of the field.*
  - *Code and data are easily available. The code is, for academic standards, exceptionally clean and well-documented.*
- *The authors take into account the share of the population affected by an NPI. This is not usually done for cross-country analyses, as most other papers rely on the OXGCRT dataset, which only has national data. (This also means that the "effective dataset size" of this study is larger than 20 countries)*

It's always easy to come up with many ways to improve any modelling study. So we could now spend months in the revision process, implementing these. But I'm pretty sure that the policy-relevant conclusions would barely change. This paper is clearly already one of the better papers on this topics, and rather than requesting changes to satisfy my maybe idiosyncratic tastes, I want this to be published soon to raise the sanity waterline of the field.

Basically, I'm happy to recommend acceptance of the manuscript as is. I group my suggestions into three groups:

· *Priority 1: These are easy to address and they do improve the paper, so I recommend you implement them. If the "priority 1" suggestions are addressed, then I'll definitely recommend acceptance. The suggestions of priority 2 and 3 are strictly optional.*

· *Priority 2: These points are a bit more work to address. Only address them if you think my point is reasonable and worth the effort. Please don't make any changes if you're not convinced of it.*

· *Priority 3: These points are to some degree a matter of taste. Again, only address them if you think my point is reasonable and worth the effort. Please don't make any changes if you're not convinced of it.*

Response to R1.1: We are delighted to read such positive comments regarding our work. Indeed, we devoted a lot of attention to this study and we are very happy to see this is being recognized. We appreciate the excellent comments and helpful suggestions that you provided, and we decided to address them all regardless of their priority.

R1.2: "*Priority 1: NPI definitions and names - Table 1:For interpreting this study, it crucial that readers can understand the NPIs studied. I suggest that you describe NPI definitions in more detail. For example, the "event ban" definition refers to cancellation of gatherings of 50 people or more, but the supplement reveals that the NPI was recorded even is only events of >5000 were banned. Similarly, the definition for "venue closure" is quite vague. Was this NPI recorded if any of these things were closed? There are many recreational venues, so did the closure of night clubs (but nothing else) suffice to trigger this NPI? What was the limit on gathering size for a "gathering ban" to be recorded?*

I am aware that it is hard to define these NPIs so that they carve reality well. Any definition will still require many judgement calls from the data collectors. Other papers get around this by simply using public datasets (e.g. OXCGRT), but, of course, these datasets have the exact same problems. The authors should be commended for collecting their own data and putting effort into validating it. I simply recommend describing the NPIs a bit more, even if the working definitions for the collection were somewhat vague. E.g. what did an NPI of this type typically look like (e.g. was the limit on events typically at 5000 people or at 50)?

In a similar vein, you could probably make the manuscript more reader-friendly

by coming up with better, more intuitive NPI names. "E.g. "ban of large gatherings" instead of "events ban". Or "closure of leisures time venues and/or gastronomy" instead of venue closure. Just spitballing here, and I don't advocate for these particular

examples. But maybe you can come up with names that more intuitively hint at the definitions. Currently, one first has to read and understand Table 1 before one gets an idea what is meant by some of the NPIs."

Response to R1.2: We are very thankful for this input. We have carefully revised the namings and definitions of the NPIs. Here, we kindly refer to our revised Table 1. The main changes are the following:

<u>'event ban' → 'ban of large gatherings'</u>: This refers to a ban of large gatherings of more than 50 people. In many countries, this was communicated as a ban of events or mass events, and hence the name 'event ban' in our original submission. However, we agree that renaming this NPI will help readers. Following your suggestion, we renamed the NPI from 'event ban' to 'ban of large gatherings' and revised the definition, which now reads as follows: 'A ban of public or private gatherings involving more than 50 people'.

<u>'gathering ban' → 'ban of small gatherings'</u>: Following your suggestion, we renamed the NPI 'gathering ban' to 'ban of small gatherings' and revised the definition accordingly, which now reads as follows: 'Ban of public or private gatherings involving less than 50 people'. For instance, a ban of gatherings of more than 30 people would be considered as a 'ban of small gatherings', whereas a ban of gatherings of more than 60 people would only be considered as a 'ban of large gatherings'.

<u>'venue closure':</u> We chose to keep the name of this NPI as we think it provides a short and comprehensive summary of the measures. Nevertheless, we agree that the description could be more precise. We thus revised the definition in the manuscript, which now reads as follows: 'Full-day closure of venues (i.e., the closure of some or all walk-in non-essential businesses like bars and restaurants, shops, and recreational facilities).' We would like to add two remarks on this definition. First, note that it was required that the venue closure applied for the whole day (e.g., we did not consider the closure of bars and restaurants only during night or evening hours). Second, note that, in most cases, all non-essential businesses were closed at the same time during the first wave of COVID-19. However, there are a few exceptions where venue closures became more stringent over time. In these cases, the implementation date of the NPI referred to the earliest date when some non-essential businesses were closed. We provide two examples for this in S1 Appendix 8.2. One is Belgium, where bars and restaurants were ordered to close on April 14 (see https://www.vrt.be/vrtnws/en/2020/03/14/tensions-as-belgium-closes-bars-and-restaurants/), while other non-essential businesses followed only a few days later (https://www.reuters.com/article/health-coronavirus-belgium-lockdown-idUSS8N2K307D). The other is Austria, where shops were ordered to close on March 15, while bars and restaurants were allowed to stay open until 3pm (see https://www.bundeskanzleramt.gv.at/bundeskanzleramt/nachrichten-der-bundesregierung/2020/bundesregierung-praesentiert-aktuelle-beschluesse-zum-coronavirus.html). In

both cases, the earlier dates (April 14 and March 15) were considered as the dates when ‘venue closures’ were implemented.

‘work ban’ → ‘work-from-home order’: During data collection, we referred to a work ban as a mandatory order to work from home (i.e., home office). Indeed, this is not adequately reflected in the previous name. Hence, we renamed this NPI to ‘work-from-home order’. Accordingly, we also clarified the definition, which now reads as follows: ‘Mandatory order to work from home (i.e., mostly related to office workers) if it is not essential to continue working at the workplace (as is mostly the case for, e.g., factories, laboratories, supermarkets, and pharmacies)’. Below (see Response to R1.5), we elaborate more on the meaning of this NPI and its definition..

R1.3: *“Communicating the hierarchy of NPIs better: Taking the NPI definitions in Table 1 literally, then a "work ban" implies a "venue closure". "Closure of non-essential business activities" implies "Closure of venues for recreational activities and/or shops, bars, and restaurants". So the effect you estimate for the "work ban" is not the effect of "Closure of non-essential business activities", but rather the additional effect of closing the remaining non-essential business activities, when the effect of closing "venues" has already been accounted for. This should be explained somewhere. The same probably goes for "gathering ban" and "event ban".“*

Response to R1.3: We agree that the hierarchy of NPIs should be discussed in greater detail. The following hierarchies are implicitly following from the definitions of our NPIs:

- A ban of small gatherings always also implies a ban of large gatherings.
- A stay-at-home order always also implies a ban of small and large gatherings and venue closures.

These hierarchies are noted in the manuscript (see Section 2.1, last sentence in the second paragraph): ‘Note that stay-at-home orders always implied bans of gatherings and venue closures, and bans of large gatherings implied bans of small gatherings. That is, for instance, if a country implemented a ban of small gatherings without yet having implemented a ban of large gatherings, then the implementation date for the ban of small and the ban of large gatherings is the same.’

The following hierarchies are observed in our dataset but they do not implicitly follow from the definitions of our NPIs:

- A ban of small gatherings does not necessarily imply a venue closure, although this happened to be the case for *most* countries, except Sweden, where recreational facilities, bars, and restaurants were allowed to stay open.

- A work-from-home order does not necessarily imply a venue closure, although this happened to be the case in our data for *all* countries during the first epidemic wave. Note, however, that there can be exceptions in subsequent waves. For instance, in Switzerland, people are still ordered to work from home while venues (fitness centers, bars, and restaurants) are allowed to reopen.

These observations are added to S1 Appendix 8.1.

R1.4: *"Clarify Figures 4d: You write: "Regarding the magnitude of the effects, we could be at least 91% sure that three NPIs simultaneously lead to a reduction in the number of new infections of more than 10%." But the way I understood Figure 4d, it shows that you can be 91% sure that there are 3 NPIs which each have an effect of >10%. Please clarify."*

Response to R1.4: Well spotted. Following your suggestion, we corrected this. Analogously, we also changed the sentence before in reference to Figure 4c. The revised sentences read as follows: 'Thereby, we could be at least 97% sure that there are five NPIs, which each lead to a reduction in the number of new infections. Regarding the magnitude of the effects, we could be at least 91% sure that there are three NPIs, which each lead to a reduction in the number of new infections of more than 10%.'

R1.5: **"***Priority 2 (These points are a bit more work to address. Only address them if you think my point is reasonable and worth the effort.):*

Work ban:

· *I'm confused about this NPI. Does this NPI actually refer to closure of all non-essential businesses activities (including the closure of factories, offices, and so on)? If so:*

o *1. This should be explained better in the definition. From the current definition, the "work ban" NPI looks very similar to Brauner's "most businesses closed"*

o *2. I don't think any country actually did close factories, right?*

· *In an alternative interpretation, this NPI refers to face-to-face, customer-facing businesses only, as in Brauner et al. If so:*

o *1. The name "work ban" is very misleading. Most work does not happen in face-to-face, customer facing businesses, but in offices, factories, and so on.*

· *I tried to figure out what was meant by looking through a few sources. The "work ban" is recorded for very few countries, implying that probably the former definition is meant. However, I'm still not sure, because:*

o *The source for France doesn't work, as it links to the current COVID page*

o *For Ireland, the source doesn't contain info on work ban, as far as I can see. But it says that it was "Last updated on 16 September 2020"*

o *For Luxembourg, it looks like only face-to-face businesses were closed.*

· *If the authors also think that the definition of work ban might be a bit shaky, I suggest to consider removing this NPI. But it's also well possible that there was a consistent definition during the data collection. In either case, I suggest communicating this a bit more clearly."*

Response to R1.5: We acknowledge that our original definition of the NPI 'work ban' may have caused confusion. Yet, there was a consistent definition applied during data collection and we think that changing the name and clarifying the definition will help to communicate more clearly what is meant with this NPI. To improve upon this, we renamed the NPI from 'work ban' to 'work-from-home order', defining it as a mandatory order to do work from home if possible (see response to R1.3). As you suspected, the 'work-from-home order' typically did not include factories, but there were exceptions, e.g., California temporarily also shut down factories. However, this is the exception rather than the rule and, thus, we consider the 'work-from-home order' to be applied to office work. Furthermore, note that while a strong recommendation to do home office was communicated in most countries, there are only a handful of countries that issued mandatory orders that actually prohibited people from going to their workplace/office.

In conclusion, we refrained from removing the NPI 'work-from-home order' but instead followed your advice to clarify the name and definition that was consistently applied during data collection.

R1.6 "*Most readers won't read the supplement and thereby don't get a feeling for how robust the results are. Maybe you can include a summary figure that aggregates the results from all sensitivity analyses in the main text.*"

Response to R1.6: Thank you very much for this great suggestion. We have added a figure to our manuscript (Figure 5) that summarizes the results from all sensitivity analyses.

R1.7 "*Priority 3 (These points are even more idiosyncratic/a matter of taste. Again, only address them if you think my point is reasonable and worth the effort. Please don't make any changes if you're not convinced of it.)*

Table 2b: If your "event ban" refers to events with >50 attendants (as the definition says), then this seems more similar to Brauner's Gatherings < 100. But it doesn't matter much, as Gatherings <1000 and Gatherings<100 are highly collinear in Brauner et al."

Response to R1.7: Thank you very much for pointing this out. We updated Table 2b to compare the effect of event bans (now referred to as the 'bans on large gatherings') with the sum of the effects of Gatherings <1000 and Gatherings<100. This led to small changes in the ranking of the effects, but the overall conclusions that we drew from this comparison in the manuscript remain unchanged.

R1.8: "*I don't think that it's clear that NPI effects are delayed in time. E.g., for schools closures, they are clearly not. Also, I expect this time delayed effect to just trade-off with the infection-to-case confirmation delay, which you infer from the data. However, this is mostly a matter of taste, and the sensitivity analysis shows that it doesn't matter.*"

Response to R1.8: We agree that the delay of the effects of NPIs can vary from NPI to NPI, and that the effects of some NPIs like school closures may be immediate. However, we feel it is important to note that we are not modeling the implementation of the NPIs, but an intermediate phase until the final 'permanent' effect of the measure is achieved. This may be reasonable even for an NPI such as school closure: The families have to find out a way to handle the new situation, and it may take some time until this is settled. For some other measures, we expect the effect to be delayed because people may not immediately adhere to measures and/or authorities may not enforce the measures on the very same day they are in effect. Furthermore, we are in doubt about this delay to trade-off with the infection-to-case reporting delay. The latter has a distribution that is constant over time, and, hence, it cannot adapt to single events in time. The delayed effect reduces the effectiveness of the measures on the contagious

subjects in the first days, whereas the infection-to-case confirmation delay determines when infected subjects are confirmed.

It would of course be more realistic to model the delay such that it can vary from NPI to NPI. However, since the information on this variation will be very weak (or absent) in the data available, we hesitated to include this step. Instead we made a choice which may be a realistic guess for the average delay. This seems more convincing to us than a simple step function.

But regardless of that, our analysis shows that the results are not sensitive to the specification of the delay, and, thus, we agree that the specification is to some degree a matter of taste. We would keep our model specification with the delayed effect, as it also hints at the comparatively minor negligible influence of detailed modeling choices in the comparison with Brauner et al. [Brauner 2020].

R1.9: *"It would be mildly interesting to see Brauner et al.'s model with your data. Especially, if you want to corroborate the following claim (Discussion) some more: "The check indicated that the choice of countries and definition of NPIs has a larger influence on the estimated effects than the detailed choices in modeling". Again, absolutely not necessary, just mildly interesting."*

Response to R1.9: We agree that it makes sense to corroborate the above claim by comparing the results with our data from both models. We thus ran the model by Brauner et al. on our data. For this, we discarded the population-weighting of NPIs and, analogously to Brauner et al., encoded NPIs as binary variables that equal 1 if the NPI is implemented in more than 75% of its population, and 0 otherwise. Furthermore, we used the cases-only-model from Brauner et al. in order to avoid any influence from incorporating data on the number of new deaths.

The results from the comparison with our data using the model by Brauner et al. and our model are shown in Table 2b. In the manuscript, we summarize the results as follows (see Section 3.4): 'When applying both models to our data, the overall ranking is still similar but with small differences in the estimates for some NPIs, in particular a higher estimate for the effect of school closures and a lower estimate for the effect of bans of small gatherings as compared to the model by Brauner et al. (Tbl. 2b).'

We think that the additional comparison does not alter our conclusion that 'the choice of countries and definition of NPIs has a larger influence on the estimated effects than the detailed choices in modeling'. Although Table 2b suggests a small influence from detailed modeling, taken together with the results from Table 2a (both models on the data by Brauner et al.), the influence is still smaller as compared to the influence from the choice of countries and definition of NPIs.

R1.10: *"Section 4.2 is probably not interesting to most people. If it was me, I'd maybe shorten it to 3 sentences that summarise the key difference, and put the full section into the appendix. The paper is becoming a bit long-winded towards the end, anyway."*

Response to R1.10: Thank you for this suggestion. We moved the full section from the manuscript to S1 Appendix 2 and summarized key differences in one paragraph in Section 4.2 of the manuscript: 'For instance, Flaxman et al. [Flaxman 2020] makes explicit assumptions on the distribution of the time from infection-to-case confirmation, which is estimated from data in the study by Brauner et al. and our study. Furthermore, modeling of the NPI effects was refined by considering country-specific effects in Brauner et al. and by taking into account regional variation in the implementation of NPIs in our study. We discuss methodological aspects in comparison to Flaxman et al. and Brauner et al. in more detail in S1 Appendix 2.'

R1.11: *"Running the model with death data seems like a relatively cheap and robust way to analyse the robustness of the results, and rule out (to some degree) a large influence of changes in the ascertainment rate."*

Response to R1.11: We agree that it is easily possible to run our model on the number of new deaths. The results from this analysis (using prior distributions on the delay from infection to death from Brauner et al.) are shown in the figure below and are compared with the results when the number of new cases is the outcome. We observe similarities but also differences in the estimated effects for two NPIs, namely, venue closure and bans of small gatherings. It is not unexpected that there are differences in the estimated effects. In fact, there are two reasons for why we believe it is not a good idea to analyse deaths in this study simply as part of a robustness check.

First, analysing deaths does not really constitute a robustness check as one could expect different results for deaths as compared to cases. The reason is that cases reflect the severity of the pandemic in the entire population, whereas deaths arise mostly in a subgroup of the population (the elderly and certain risk groups). Although the number of cases and deaths correlate to some extent, we still expect the effects of NPIs to refer to different (sub-)populations, and thus we would not consider this to be part of a typical sensitivity analysis.

Second, although it is relatively cheap to run the model on the number of new deaths, we think that it would require a similarly thorough investigation to model the number of new deaths as to model the number of new cases, including model checks and sensitivity analysis. This is all beyond a simple robustness check and thus beyond the scope of this study. We acknowledge the importance of analysing deaths but we would

not feel comfortable with showing results from a quick analysis and rather make this analysis subject to future work.

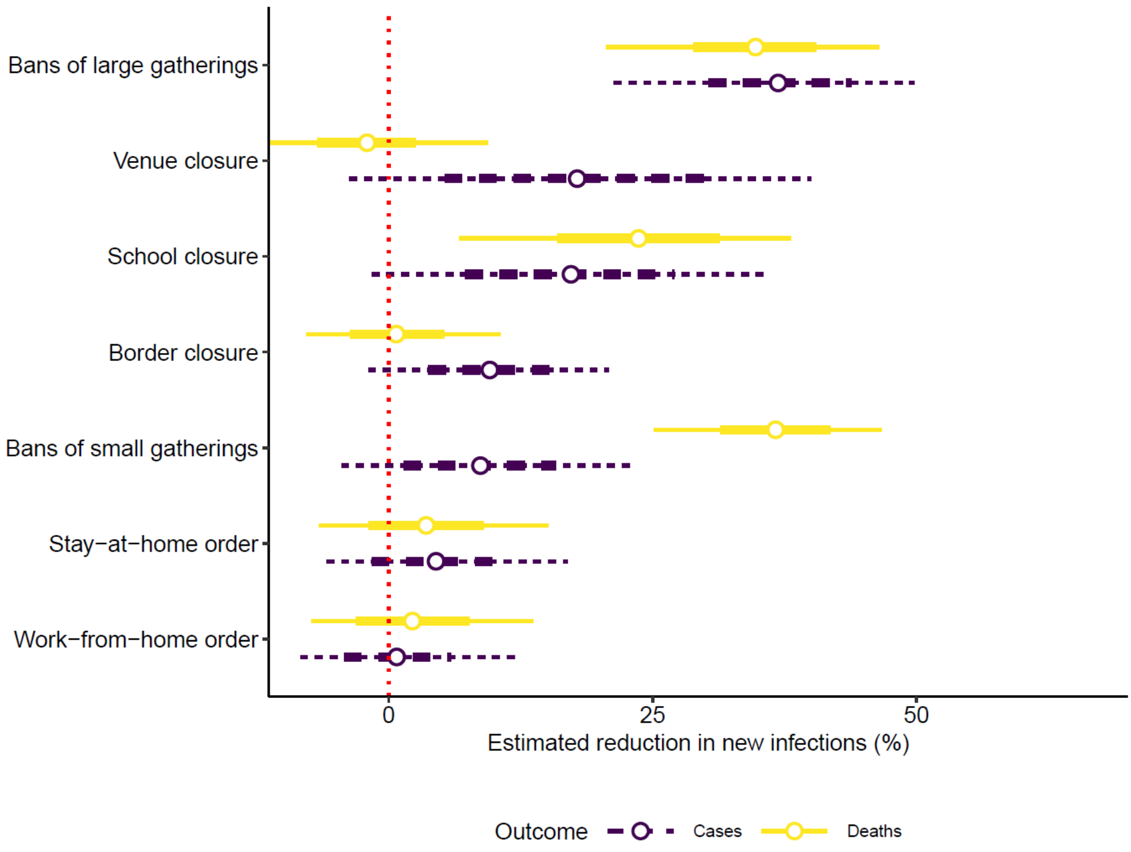

R1.12: *"I find the NPI effect prior a bit un-intuitive. I think it's more natural to assume that small effects are more likely a priori than large effects."*

Response to R1.12: We welcome the opportunity to elaborate on the choice of our prior. We specifically refrained from making the assumption that small effects are more likely than large effects, although we acknowledge that this assumption may not be unreasonable. Our choice was inspired by the thought that we would want to be a priori *un*informative about the positive effect of NPIs, so that each NPI could have a small or large effect with the same probability and the data should inform which. This is based on the intuition that it is a priori also possible that a single or just two measures were effective in reducing the number of new infections.

R1.13: *"Page 3, line 52: I think you meant to say "ambiguous", and not "not ambiguous"*

Response to R1.13: Thank you very much, we have corrected this mistake.

R1.14: *“Figure 1: "Number of new cases per 100,000 (rolling 7-day mean) until NPIs were implemented across countries." - I think it would be easier to understand if you wrote "when NPIs were first implemented”*

Response to R1.14: This is a good suggestion and we have revised the figure caption accordingly.

R1.15: *“page 17, line 175: "The authors used similar priors for the distribution of the time from infection to reporting and for the generation time distribution, but estimated the latter explicitly as part of fitting the model." - Brauner et al estimate delay distribution and the generative interval distribution from data. I think what you mean to say is that in contrast to your paper, Brauner et al also estimates the GI from data?*

Response to R1.15: Again, this is a good suggestion and we rephrased to ‘The authors used similar priors for the distribution of the time from infection to reporting and for the generation time distribution, but, in contrast to our work, Brauner et al. estimated the latter explicitly from data as part of fitting the model.’

R1.16: *“Figure 5: In the legend, observed N should not have a shaded frame around it, as this implies the credible intervals.”*

Response to A.16: We have updated the figure legend and removed the shaded frame around the observed *N*.

R1.17: *“Table 2: "Note that, in these analyses, we report cumulative effects for gathering bans and businesses closed." - I would add "as in Brauner et al.", to make it clear why you are doing this.”*

Response to R1.17: Good point, we have added ‘as in Brauner et al.’ to the note.

Response to Reviewer #2

Dear Reviewer #2,

Thank you for your critical comments.

R2.1.: *"A semi-mechanistic Bayesian hierarchical model to assess the effectiveness of seven NPIs in curtailing the number of COVID-19 cases in 20 countries using data for first wave is proposed. This is an interesting study, but in addition to the comments below, I believe that it does not provide significant advancement in the field beyond that in the study in Brauner et al. mentioned in the paper."*

Response to R2.1.: We acknowledge that our study is methodologically similar to the study by Brauner et al. [Brauner 2020], but we politely disagree with the assertion that our study *"does not provide significant advancement"*. There are multiple, important differences in modeling and data that distinguish our work from Brauner et al.:

1. Most importantly, we take into account the effect of non-pharmaceutical interventions (NPIs) at the regional (subnational) level. For instance, in the US, we consider that NPIs were implemented at different points in time across states. Regarding modeling, we take the subnational variation in the implementation of NPIs into account by 'weighting' the effects of an NPI with the share of the national population that is affected by it at any point in time. This can have a large influence on the estimated effects, particularly for countries like the US where most NPIs were not implemented at the national level.
2. We consider that the effects of NPIs can be delayed by modeling them with a first-order spline.
3. We use a more diffuse prior for the positive effects of NPIs. Hence, we refrain from formulating a prior belief about their effect sizes. This is different to Brauner et al., who incorporate their belief that small effect sizes are a priori more likely than large effect sizes.
4. Regarding data, both studies comprise a different set of countries.

All methodological differences and similarities are transparently described in Section 3.4 (the comparison with Brauner et al.) and further discussed in Section 4.2 (methodological aspects in relation to Flaxman et al. [Flaxman 2020] and Brauner et al.).

It is not known how detailed modeling choices and different data influence the estimated effect of NPIs. We find that different data exhibits a comparatively larger influence on the model estimates than detailed modeling. Note that many other studies

in the field use data from the same single source (i.e. data from the Oxford Government Response Tracker). Our conclusion implies that the results from all these analyses are subject to the same influence of subjective encoding decisions, which is not reflected in the uncertainty regarding the estimated effects of NPIs.

Finally, we think that it is extremely relevant to check the robustness of findings of interest to public policy. Of note, there is still large uncertainty around the NPI effects during the first wave. For instance, the findings from the influential and similar work by Flaxman et al. have been subject to an extensive debate in *Nature* questioning whether different modeling assumptions would still result in consistent findings [Soltez 2020]. Another well-cited study on the effectiveness of stay-at-home orders [Bendavid 2021] raised methodological concerns [Besançon 2021]. This was also accompanied by a heated public discourse in the media around the study's conclusions (see https://www.washingtonpost.com/dc-md-va/2020/12/16/john-ioannidis-coronavirus-lockdowns-fox-news/).

R2.2: "*No motivation on why using data for the first pandemic wave at a time when many countries have or are already experiencing second or third waves is appropriate has been provided. Hence, it is not clear how useful this study is in the current fight against the pandemic, especially since most of the measures considered in this study were relaxed or completely discontinued months ago in most of the study countries.*"

Response to R2.2: The findings regarding the effects of NPIs during the first wave are still inconclusive (see Introduction, second paragraph). Thus, it still is subject to debate to what extent different measures were effective in the onset of a pandemic. We add to this by contributing new findings from a model with novel extensions for estimating the NPI effects.

Note that we specifically avoided any claims on immediate relevance to public health policy and positioned our study as a retrospective analysis of the first epidemic wave. The relevance for public health polity has been strengthened with the following addition to the conclusion (see Section 4.6): 'Our analysis makes a contribution to the emerging evidence about the effectiveness of different NPIs in the first epidemic wave. Ideally, such studies could inform public health policy in the onset of future epidemics, as well as modeling efforts related to later waves.'

R2.3: "*Use of the phrase NPIs in this manuscript is misleading. NPIs are broadly defined to include social-distancing, lockdowns, wearing of masks in public, contact-tracing, quarantine, isolation, hand-washing, etc. I believe the seven measures in the study fall under one or two broad kinds of NPIs, e.g., social-distancing and*

lockdowns, especially since most of them overlap. Strangely, the manuscript is silent on how these overlaps between the selected control measures were handled or eliminated."

Response to R2.3: The term *"NPI"* is commonly used in related work (e.g., Brauner et al. and Flaxman et al.) to describe a broad set of measures involving social distancing measures. Our set of measures is quite similar to the one in Brauner et al., which also facilitates our comparison with their study. We agree that our NPIs could broadly be summarized under social distancing and lockdown measures. Yet, we would refrain from summarizing other measures into NPIs. For instance, one could argue that wearing masks and hand-washing is rather a pharmaceutical than non-pharmaceutical intervention and one could also question whether contact-tracing should be regarded as an intervention.

We would also kindly like to note that we are currently conducting a systematic review of the NPI literature, thereby observing high variability in the terminology used to describe similar sets of measures. Often distinctive terms are used interchangeably and often the measures themselves are not adequately defined. In contrast to that, we followed the terminology in work most related to ours (e.g., Brauner et al and Flaxman et al.) and carefully defined our set of measures. Thereby, we specifically avoided the term *"lockdown"* as it is often used in different contexts, sometimes referring to a single measure (e.g., stay-at-home order) or multiple measures, and can easily be misunderstood (we made this experience with media journalists after publishing our first preprint on the topic where we used the term 'lockdown' in place of 'stay-at-home order'; and Flaxman et al. were also confronted more than once with their broad definition of a 'lockdown' in their NPI study).

As for the overlap between measures, this was also noted by reviewer #1, and we carefully revised our manuscript. First, following suggestions from reviewer #1, we revised the name of some of our NPIs ('event ban' → 'ban of small gatherings', 'gathering ban' → 'ban of large gatherings', and 'work ban' → 'work-from-home order'). Second, we refined the definitions of some NPIs (see revised definitions in Table 1 for ban of small gatherings, venue closure, ban of large gatherings, and work-from-home order) and provide further examples that illustrate how the definitions were applied during data collection (see S1 Appendix 8.2). Third, we describe the overlap between NPIs or what reviewer #1 referred to as the hierarchy of NPIs (see our revisions at the end of the second paragraph in Section 2.1 of our manuscript and S1 Appendix 8.1): 'Note that stay-at-home orders always implied bans of gatherings and venue closures, and bans of large gatherings implied bans of small gatherings. That is, for instance, if a country implemented a ban of small gatherings without yet having implemented a ban of large gatherings, then the implementation date for the ban of small and the ban of large gatherings is the same. In contrast to this, a ban of small gatherings alone does not necessarily imply a venue closure (see for example Sweden where recreational facilities, bars, and restaurants were allowed to stay open despite a

ban of small gatherings). Similarly, a work-from-home order does not necessarily imply a venue closure, although this happened to be the case for all countries in our data during the first epidemic wave.').

R2.4: "*The authors say, "On the one hand, the national strategies consisted of similar NPIs, which we can expect to work in a similar manner, despite cultural and organisational differences between countries." This is not true, since not all the countries, e.g., the US, implemented the identified strategies nationally. Different states in the US implemented different strategies with different stringency levels. I also think it is not reasonable not to consider some of the countries that have been able to manage the pandemic successfully through the use of NPIs in this study."*

Response to R2.4: We agree with the reviewer that not all countries implemented the identified strategies nationally. However, this is taken explicitly into account in our modeling approach – in contrast to most (not all, see Hsiang et al.) other studies. Our raw data consists of measures implemented at the state-level in the US, which is considered during modeling by 'weighting' the effects of NPIs with the share of the national population that is affected by an NPI at any point in time. The fact that we take into account subnational implementation of NPIs in all countries is also recognized by reviewer #1, who notes that this also implies that the "'*effective dataset size' of this study is larger than 20 countries"*. Hence, we regard this to be a strength and not a problem of our approach.

We believe that it may have been confusing to speak only of "national strategies" at the start of the third paragraph in Section 2.1. We added "national (and subnational) strategies" to make it clear that we take into account subnational strategies. The whole paragraph now reads as follows: 'On the one hand, the national (and subnational) strategies consisted of similar NPIs, which we can expect to work in a similar manner, despite cultural and organisational differences between countries. On the other hand, the national (and subnational) strategies differed in the choice, timing, and sequencing of NPIs. Taken together, the setting of this study resembles that of a natural experiment, which allows us to learn about the effects of different NPIs. However, in countries with a federal structure, the timing of NPIs may differ between regions (e.g., states or territories). In countries with such regional variation, NPI data was collected at the regional level and, similar to Hsiang et al., we took into account the cumulative share of the country's population that is affected by an NPI.'

We also agree with the reviewer that it would be of interest to use all successful countries in order to obtain information about the effect of NPIs. However, learning from many countries about the effect of an NPI implies that we have to assume that NPIs act in a similar way in different countries. We are much in doubt whether this

assumption is justified with respect to non-Western countries, in particular the countries from East Asia. There are cultural differences and differences with respect to experiences from previous pandemics. To ensure sufficient homogeneity, we decided to exclude these countries.

R2.5: "*I am not sure I understand what is going on from Figure 2, especially since the flow branch 1 has no direction.*"

Response to R2.5: We have added the direction to flow branch 1 in Figure 2 and hope it aids understanding of the figure, which summarizes the model structure. We think it provides a good high-level summary of the mechanistic modeling process, similar to the visual summaries by Flaxman et al. and Brauner et al. But of course, we welcome any further suggestions on how to make this figure more comprehensible.

R2.6: "*It is strange that the new infections as modeled in Eq. 1 are generated only from the transmission rate, and the number of contagious subjects, but not also the uninfected.*"

Response to R2.6: We acknowledge that one should eventually take into account the number of susceptible people in the population. However, we think that one can safely neglect this adjustment in the first epidemic wave, as it has been shown that even in countries with a severe first epidemic wave, seroprevalence was still very low. Brauner et al. do also not take into account the decrease in the susceptible population but report results from a different model that does, showing similar effectiveness for the NPIs (see Brauner 2020, Supplementary Material E.2).

We revised our manuscript and added an additional item to our limitations (see Section 4.4): 'Fifth, it is not considered in our analysis that the number of susceptible people in the population decreases as the number of people that were already infected increases over time. However, we think this limitation is not of particular concern during the first epidemic wave. A study in Spain, which experienced a severe first epidemic wave, showed that prevalence was only around five percent in the population, indicating that the large majority of the Spanish population was still susceptible after the first epidemic wave [Pollán 2020].'

R2.7: "*Also, introducing NPIs as in this equations leads to unidentifiability, where different inputs can lead to the same output. How was this issue handled?*"

Response to R2.7: Thank you for raising this important question and thus giving us the opportunity to elaborate how identifiability in our work was ensured. Specifically, we followed recommendations for Bayesian modeling [Gelman 2020] and performed a simulation study to check whether our model can recover the true effects of NPIs from fake data. We refer to this in the manuscript (see new Subsection 2.3 Simulation-based study): 'Highly parametrized models may raise concerns regarding the identifiability of individual model parameters. It is thus recommended to check if the true parameters can be recovered from the model using fake data simulation [Gelman 2020]. We performed such a simulation-based study, thereby demonstrating that it is possible to recover the true effect of NPIs within the uncertainty implied by the fitted posterior distribution of our model (see S1 Appendix 3).'

References

Brauner JM, Mindermann S, Sharma M, Johnston D, Salvatier J, Gavenčiak T, et al. Inferring the effectiveness of government interventions against COVID-19. Science. 2020 4;p. Eabd9338. Available from: https://www.sciencemag.org/lookup/doi/10.1126/science.abd9338

Soltesz K, Gustafsson F, Timpka T, Jaldén J, Jidling C, Heimerson A, et al. The effect of interventions on COVID-19. Nature. 2020 4;588(7839):E26–E28. Available from: http://www.nature.com/articles/s41586-020-3025-y.

Besançon L, Meyerowitz‐Katz G, Flahault A. Sample size, timing, and other confounding factors: towards a fair assessment of stay‐at-home orders. European Journal of Clinical Investigation. 2021 Feb 12. Available from: https://doi.org/10.1111/eci.13518

Gelman A, Vehtari A, Simpson D, Margossian CC, Carpenter B, Yao Y, Kennedy L, Gabry J, Bürkner PC, Modrák M. Bayesian workflow. arXiv preprint arXiv:2011.01808. 2020 Nov 3. Available from: https://arxiv.org/abs/2011.01808

Flaxman S, Mishra S, Gandy A, Unwin HJT, Mellan TA, Coupland H, et al. Estimating the effects of non-pharmaceutical interventions on COVID-19 in Europe. Nature. 2020 4;584(7820):257–261. Available from: http://www.nature.com/articles/s41586-020-2405-7

Bendavid E, Oh C, Bhattacharya J, Ioannidis JP. Assessing mandatory stay‐at‐home and business closure effects on the spread of COVID‐19. European journal of clinical investigation. 2021 Apr;51(4):e13484. Available from: https://doi.org/10.1111/eci.13484

Sharma M, Mindermann S, Rogers-Smith C, Leech G, Snodin B, Ahuja J, et al. Understanding the effectiveness of government interventions in Europe's second wave of COVID-19. medRxiv. 2021; Available from: https://doi.org/10.1101/2021.03.25.21254330.

Pollán M, Pérez-Gómez B, Pastor-Barriuso R, Oteo J, Hernán MA, Pérez-Olmeda M, et al. Prevalence of SARS-CoV-2 in Spain (ENE-COVID): a nationwide, population-based seroepidemiological study. The Lancet. 2020 8;396(10250):535–544. Available from: https://doi.org/10.1016/S0140-6736(20)31483-5.
